# Differences in maternal and early child nutritional status by offspring sex in lowland Nepal

**DOI:** 10.1002/ajhb.23637

**Published:** 2021-07-06

**Authors:** Naomi M. Saville, Helen Harris‐Fry, Akanksha Marphatia, Alice Reid, Mario Cortina‐Borja, Dharma S. Manandhar, Jonathan C. Wells

**Affiliations:** ^1^ Institute for Global Health (IGH) University College London (UCL) London UK; ^2^ Department of Population Health London School of Hygiene and Tropical Medicine London UK; ^3^ Department of Geography University of Cambridge Cambridge UK; ^4^ Section of Clinical Epidemiology, Nutrition and Biostatistics, Population Policy and Practice Research and Teaching Department, Great Ormond Street Institute of Child Health (ICH) University College London (UCL) London UK; ^5^ Mother and Infant Research Activities (MIRA) Kathmandu Nepal

## Abstract

**Objective:**

On average, boys grow faster than girls in early life but appear more susceptible to undernutrition. We investigated sex differences in early child growth, and whether maternal nutritional status and diet differed by offspring sex during and after pregnancy in an undernourished population.

**Methods:**

We analyzed longitudinal data from a cluster‐randomized trial from plains Nepal, stratifying results by child or gestational age. Children's outcomes (0–20 months) were weight, length, and head circumference and their *z*‐scores relative to WHO reference data in 2‐monthly intervals (n range: 24837 to 25 946). Maternal outcomes were mid‐upper arm circumference (MUAC), and body mass index (BMI) during pregnancy (12–40 weeks) (*n* = 5550 and n = 5519) and postpartum (n = 15 710 and n = 15 356), and diet in pregnancy. We fitted unadjusted and adjusted mixed‐effects linear and logistic regression models comparing boys with girls.

**Results:**

Boys were larger than girls, however relative to their sex‐specific reference they had lower length and head circumference *z*‐scores from birth to 12 months, but higher weight‐for‐length *z*‐scores from 0 to 6 months. Mothers of sons had higher MUAC and BMI around 36 weeks gestation but no other differences in pregnancy diets or pregnancy/postpartum maternal anthropometry were detected. Larger sex differences in children's size in the food supplementation study arm suggest that food restriction in pregnancy may limit fetal growth of boys more than girls.

**Conclusions:**

Generally, mothers' anthropometry and dietary intake do not differ according to offspring sex. As boys are consistently larger, we expect that poor maternal nutritional status may compromise their growth more than girls. Copyright © 2021 John Wiley & Sons, Ltd.

## 1 INTRODUCTION

In high‐income settings, boys grow faster on average than girls during fetal life and early infancy (Freeman et al., 1995; Kuczmarski et al., 2000; Rosario et al., 2011). Similarly, WHO global reference charts (Rosario et al., 2011) show that healthy boys consistently weigh more than healthy girls over the first year of life, gaining 10.4% more weight in the first 6 months (World Health Organization, Food and Agriculture Organization, & United Nations University, 2004). These differences are largely due to greater accretion of fat‐free mass (FFM) in boys compared to girls, whereas levels of fat mass (FM) are relatively similar between the sexes (Fomon et al., 1982; Wells et al., 2020). Averaging on a daily basis, Fomon and colleagues found that the cost of growth in the first 6 months was 9.8% greater in boys than girls (Fomon et al., 1982).

These differences in size and growth rate may contribute to greater total energy requirements of boys compared to girls. In a US study of breast‐fed infants, boys' energy requirements were 3–14% greater than girls over the first 2 years, depending on age (Butte et al., 2000). A similar pattern was evident in a compilation of data from the UK, the Netherlands, Chile, and China (World Health Organization et al., 2004). However, these data have been obtained in settings where conditions are close to optimal for health. In low‐income settings prone to undernutrition, sex differences in growth may be attenuated.

In a birth cohort from Jimma, Ethiopia, the difference between boys' and girls' tissue masses between birth and 6 months was smaller than in the US for FFM, but slightly larger for FM (Andersen et al., 2013; Fomon et al., 1982). Since fat contains more stored energy than protein, the net effect of these growth patterns was that the average daily cost of growth in the first 6 months was 2% greater in girls than boys, even though the median girl had 3% lower weight at 6 months. This may indicate a greater degree of growth restraint in boys, compared to the high‐income populations discussed above.

### 1.1 Maternal nutritional status by child sex

During pregnancy and exclusive breastfeeding, the entire costs of offspring growth must be met through maternal energy transfer. Greater growth rates in boys are expected to increase the energy costs of nourishing children, unless other components of energy expenditure decrease in compensation. Any greater energy transfer to sons would require their mothers either to consume greater dietary intake, or to oxidize greater levels of energy reserves, compared to mothers of daughters.

Indeed, in high‐income countries, higher intakes have been observed among mothers of sons compared with daughters, both preconceptually and during pregnancy (Mathews et al., 2008; Tamimi et al., 2003). In Chile, fetal growth of boys was affected by maternal nutritional status whereas that of girls was not. This suggests that males are more likely than girls to be growth‐limited due to maternal nutritional restrictions (Lampl et al., 2010).

Sex differences in infant growth could also impact the energy transferred during lactation. In a pooled analysis of 1115 measurements of breast‐milk intake in 12 countries, boys' milk intake was 5% greater than girls' (da Costa et al., 2010). In other species, however, sex differences in maternal milk quantity may be antagonistic to those in milk quality. In rhesus macaques, for example, a study found that mothers produced richer milk for sons but greater volumes of milk for daughters, resulting in similar total milk energy transfer to the two sexes (Hinde, 2009). Nevertheless, sons still grew faster than daughters, especially among the offspring of first‐time mothers (Hinde, 2009). This suggests that greater growth costs in sons were offset by reductions in other components of energy expenditure, but we lack similar evidence in humans.

Among under‐nourished populations, any limitation of mothers to meet greater growth costs of sons compared to daughters may lead to increased levels of under‐nutrition among boys and/or more depletion of mothers. Growth reference data are by convention expressed as *z*‐scores, allowing expected variability by age and sex to be taken into account. Using this approach, numerous recent studies have shown higher rates of boys being categorized as wasted (low weight‐for‐height) or stunted (low height‐for‐age) (Goncalves et al., 2019; Schoenbuchner et al., 2019; Thurstans et al., 2020), though differences of lower magnitude have been reported in India (Mukhopadhyay, 2016). A recent review of 76 studies in children aged 0 to 59 months found that stunting, wasting, and underweight were more common in boys than in girls; these differences tended to be lower in South Asian populations, which might indicate cultural preferences favoring sons (Thurstans et al., 2020). However, we found no studies that have tracked how sex differences manifest in mothers and children over time–from pregnancy, birth, and early childhood.

The objective of this study was to explore whether limitations in maternal nutrition might account for sex differences in growth in low‐income settings. We analyzed data from a cluster‐randomized controlled trial in lowland Nepal from a population of poor women who tend to marry and bear children early but also vary in relative wealth and education.

This study addresses the following hypotheses:

H_0_1. Boys have larger absolute weight, length, and head circumference but relatively low length‐for‐age (LAZ), weight‐for‐length (WLZ), and head‐circumference‐for‐age (HCAZ) *z*‐scores compared with girls.
H_0_2. Sons have a greater risk of stunting and wasting than daughters.
H_0_3. Differences between sons and daughters will be larger when mothers are primigravida or when the mother is undernourished (low stature or low BMI).
H_0_4. Compared with mothers of daughters, mothers of sons have higher mid‐upper arm circumference (MUAC) and body‐mass index (BMI) in pregnancy and lower MUAC and BMI during postpartum.
H_0_5. Mothers of sons consume more energy and micronutrients during pregnancy than mothers of daughters.
H_0_6. Provision of additional food to the mother during pregnancy (through cluster RCT interventions) increases the difference between the sexes in body size in the first 8 days of life.

Using observational analyses, we first compared the growth and nutritional status of boys versus girls from birth to 20 months, both in absolute terms and by expressing the data as *z*‐scores relative to WHO 2006 reference data. We then assessed the nutritional status and dietary intake of mothers of sons versus daughters through pregnancy and early infancy. Finally, we tested whether the impact of the randomized trial varied by offspring sex.

## 2 METHODS

We used the Low Birth Weight South Asia Trial (LBWSAT) study cohort for our analyses. This trial compared the effect of a participatory learning and action (PLA) women's group intervention versus standard care, with or without the addition of food or cash transfers to pregnant women (Saville et al., 2016; Saville et al., 2018). The trial showed a 78 g significant increase in birth weight within 72 h in the “PLA plus food” arm but increases of 50.5 and 28.9 g in the PLA plus cash and PLA only arms were not statistically significant (Saville et al., 2018).

### 2.1 Setting

Our Maithili‐speaking study population resides in Dhanusha and Mahottari districts in Province 2 of southern (plains) Nepal, near the border with Bihar state of India. Women in this context often suffer limited (Morrison et al., 2018) and inequitable (Harris‐Fry, Paudel, Shrestha, et al., 2018) access to food during pregnancy and the postpartum period and 40% were thin during late pregnancy, defined as MUAC <23.5 cm (Harris‐Fry, Paudel, Shrestha, et al., 2018).

Methods used in LBWSAT have been reported in detail elsewhere (Saville et al., 2016; Style et al., 2017). Briefly, 80 population clusters that were defined as Village Development Committees (VDCs) were randomized into four study arms using block randomization, stratified on the basis of how many subclusters were accessible to trucks during the monsoon and on cluster size (<6400 vs. ≥6400 population). The four strata, each comprising 20 clusters, were small inaccessible, large inaccessible, small accessible, and large accessible. Between December 2013 and March 2015, the study enrolled 25 090 women and prospectively followed some of them during “early” pregnancy before 31 weeks' gestation (*n* = 3860) and “late” pregnancy ≥31 weeks' gestation (*n* = 2872), soon after delivery (*n* = 6286), and 2–3 months after delivery (*n* = 2814). At the study endpoint (June to October 2015) as many mothers and their children as could be found (*n* = 14 085 mothers and *n* = 17 311 children respectively) were measured, when the index children were between 0 and 22 months in age. At the same time, triplicate quantitative 24‐h dietary recalls were collected on a subsample of still enrolled pregnant women between 25 and 42 weeks' gestation (*n* = 470). Gestational age was estimated from maternal recall of last menstrual period.

Informed written consent was taken from all participants in this study, and their guardians if they were aged <18 years. Research ethics approval was obtained from the Nepal Health Research Council (NHRC) (108/2012) and the University College London (UCL) Ethical Review Committee (4198/001) for primary data collection, and from the NHRC (292/2018) and the UCL Ethical Review Committee (0326/015) for the secondary analyses presented here.

### 2.2 Outcomes

**FIGURE 1:**
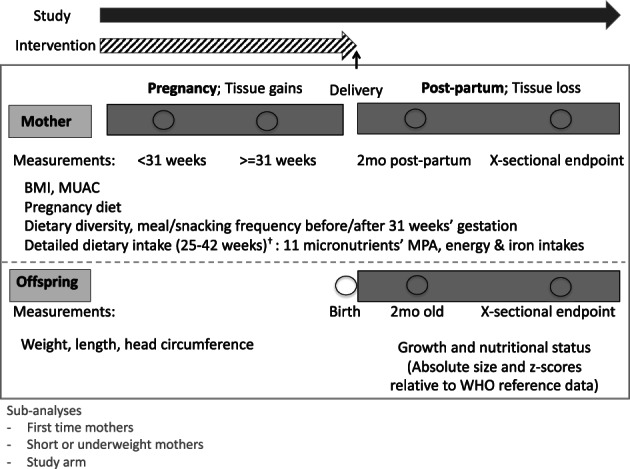
Conceptual diagram of study design to evaluate sex differences in offspring^†^ and mother's nutritional status^‡^ and mother's dietary intakes in pregnancy^§^. ^†^Offspring weight, length and head circumference measured at birth, 2 months and cross‐sectionally at endpoint combined into a mixed longitudinal dataset for analysis. ^‡^Mothers' weight, height, body mass index (BMI) and Mid‐upper Arm Circumference (MUAC) measured at 4 timepoints: before and after 31 weeks' gestation, 2 months postpartum and postpartum cross‐sectionally at endpoint combined into a mixed longitudinal dataset for analysis. ^§^Dietary diversity and meal or snack frequency between 12 and 39.9 weeks pregnancy. Detailed dietary intake in terms of energy intake and adequacy, iron intakes and Mean Probability of Adequacy (MPA) of 11 micronutrients (calcium, vitamin C, vitamin A, vitamins B1, B2, B3, B6, B9, B12, iron and zinc) estimated between 25 and 42 weeks pregnancy (average gestational age 36 weeks)

We measured mothers' MUAC and weight on up to four occasions per woman as shown in Figure 1. We measured mothers' heights in early pregnancy where possible but at other time points otherwise. Lordosis during pregnancy may account for small errors in maternal height estimation, but since the proportion of mothers of boys and girls that were measured at different timepoints are similar, we do not expect this to affect our results. Offspring length, weight, and head circumference (HC) were taken on up to three occasions per child as in Figure 1. At the endpoint, children were 0 to 22 months old. The children's data were converted to *z*‐scores for length‐for‐age (LAZ), weight‐for‐length (WLZ), and head circumference‐for‐age (HCAZ), using the WHO 2006 growth standard and the Stata command who2006, and cleaned using standard WHO criteria for plausible values (Crowe et al., 2014).

In the interviews at pre‐ and post‐31 weeks' gestation, we asked the women to recall the number of eating occasions of main meals and snacks, and the food groups consumed, in the preceding 24 h. We categorized foods consumed into 10 food groups and calculated the 24‐h Women's Dietary Diversity Score (WDDS) (FAO & FHI 360, 2016). This has been demonstrated to be a reasonable proxy for micronutrient adequacy of the diet of pregnant women (Nguyen et al., 2018).

In addition to the data collection before and after 31 weeks' gestation, detailed 24‐h dietary recall data were collected on three nonconsecutive days (between June and September) for a subsample of 470 enrolled pregnant women using a validated food atlas to aid portion size estimation (Harris‐Fry et al., 2016). We calculated energy intakes (kcal/d), usual iron intakes (mg/d), and the mean probability of adequacy (MPA) of 11 micronutrients (calcium, vitamin C, vitamin A, vitamins B1, B2, B3, B6, B9, B12, iron and zinc) (Harris‐Fry, Paudel, Shrestha, et al., 2018). We used Best Linear Unbiased Predictors (which account for within‐person variance in the three dietary recalls) to predict usual nutrient intakes (Institute of Medicine, 2003), then calculated the probability of nutritional adequacy based on known requirement distributions (FAO & Organization, 2004; Institute of Medicine, 2011). The MPA was the average of these 11 probabilities. In all trial intervention arms, there were higher odds of pregnant women consuming iron‐folic acid supplements and dietary diversity was higher in the cash arm (Harris‐Fry, Paudel, Harrisson, et al., 2018). Given these differences, we adjust analyses for the study arm.

### 2.3 Data preparation

Maternal and child data were collected across a range of gestational and child ages. Hence, we combined all measures into two mixed longitudinal cohort datasets, one for children 0 to 20 months and one for mothers who embraced pregnancy (12 to 40 weeks' gestation) and the post‐partum period (0 to 20 months). There were 18 698 pregnancies associated with 18 661 women, as 37 had two pregnancies.

We separated pregnancy measures into seven 4‐week gestational age categories between 12 and 40 weeks, and children and mothers' postpartum measures into ten 2‐month age/time categories from 0 to 20 months. We used intervals such as 12 to 15.9 weeks' gestation and 0 to 1.9 months postpartum but summarize these as 0–2 or 12–16 and so on in the text below. Anthropometric measures of 18 172 children (born of 18 147 mothers) and of 14 024 mothers and 14 030 pregnancies amounted to 25 946 child lengths, 25 531 weights, and 25 928 head circumferences. Mothers' measures amounted to 5550 pregnant and 15 710 postpartum MUAC and 5519 pregnant and 15 356 postpartum BMI measures. In terms of replicates per individual, 11 933 children had one measurement, 4546 had two, and 3386 had three measures. Similarly, 9937 women had one, 3783 had two, 1061 had three, and 159 had four measures each.

### 2.4 Statistical methods

We fitted unadjusted and adjusted multilevel mixed‐effects linear and logistic regression models for continuous and binary outcomes respectively. In both adjusted and unadjusted models, a random effect term was included in the intercept to account for clustering due to the study design. Nutritional status or maternal diet were the dependent variables, and child sex was the independent variable.

To describe the sample, we tested for differences in mothers' characteristics by child sex using Pearson's Chi‐squared test for categorical variables and *t*‐tests for continuous, normally distributed variables (Table 1 and Table S1). In preliminary analysis, we found that mothers of boys and girls did not differ with respect to age of marriage or first pregnancy, landholding size, religion, overseas labor migration from their household, study arm, randomization strata, or season of measurements. However, mothers of boys and their husbands were more educated and were more affluent. Mothers of boys were also slightly older, had higher parity, and more likely to have had four or more pregnancies previously (Table 1). We therefore adjusted using fixed effects for parity, mother's education and wealth as well as either child age (after birth) (covariate set 1) or gestational age of the mother during pregnancy (covariate set 2). We additionally adjusted for study arm and randomization strata in adjusted regression analyses as fixed effects in all models (covariates sets 1 and 2), to account for any effects of trial interventions upon outcomes and to adjust for study design. We did not adjust for maternal age in the models as the mean difference between mothers of sons and daughters was only 36.5 days, and to avoid collinearity as maternal age is highly correlated with parity.

**TABLE 1:** Respondent characteristics by sex of the offspring for women enrolled in LBWSAT for whom at least one mother or child anthropometric measure and sex of the offspring is available

Characteristic of respondent (categorical variables)	*n* ^a^	Female		Male		Total		*p*
Sex of child	18 698							<.001^b^
Female		8830	100%	0	0%	8830	47%	
Male		0	0%	9868	100%	9868	53%	
Study arm woman enrolled in	18 698							.273^c^
Control		1961	22%	2186	22%	4147	22%	
Women's group		1969	22%	2304	23%	4273	23%	
Cash		2464	28%	2752	28%	5216	28%	
Food		2436	28%	2626	27%	5062	27%	
Randomization stratum	18 698							.481^c^
Small, inaccessible		1823	21%	2104	21%	3927	21%	
Small, accessible		1908	22%	2171	22%	4079	22%	
Large, inaccessible		2551	29%	2822	29%	5373	29%	
Large, accessible		2548	29%	2771	28%	5319	28%	
Gravida (previous pregnancies)	18 650							.010^c^
0		3104	35%	3316	34%	6420	34%	
1		2358	27%	2558	26%	4916	26%	
2		1728	20%	2003	20%	3731	20%	
3		944	11%	1130	11%	2074	11%	
4 or more		668	8%	841	9%	1509	8%	
Age category of Mother	18 698							.046^c^
11–19 years		3597	41%	3843	39%	7440	40%	
20–24 years		3296	37%	3722	38%	7018	38%	
25–29 years		1456	16%	1734	18%	3190	17%	
30–55 years		481	5%	569	6%	1050	6%	
Mother's education	18 643							.004^c^
Never went to school		5814	66%	6306	64%	12 120	65%	
Primary to lower secondary		1747	20%	2009	20%	3756	20%	
Secondary and above		1235	14%	1532	16%	2767	15%	
Husband education	18 643							<.001^c^
Never went to school		4362	50%	4697	48%	9059	49%	
Primary to lower secondary		2426	28%	2661	27%	5087	27%	
Secondary and above		2008	23%	2489	25%	4497	24%	
Water source	18 641							.003^c^
Public/neighbors		2430	28%	2503	25%	4933	26%	
Own yard		2120	24%	2410	24%	4530	24%	
Inside/deep borehole		4246	48%	4932	50%	9178	49%	
Religion	18 697							.240^c^
Muslim or other		1421	16%	1526	15%	2947	16%	
Hindu		7409	84%	8341	85%	15 750	84%	
Caste	18 698							.006^c^
Dalit/Muslim‐ disadvantaged		3213	36%	3390	34%	6603	35%	
Janjati/ Terai castes ‐ middle		3746	42%	4248	43%	7994	43%	
Yadav/Brahmin ‐ advantaged		1871	21%	2230	23%	4101	22%	
Total		8830		9868		18 698		

Characteristic of respondent (continuous variables)	*n* ^a^	Female		Male		Total		*p*
Parity^d^, mean (*SD*)	18 650	1.26	*1.35*	1.34	*1.40*	1.34	*1.38*	<.001^e^
Parity^d^, median (iqr)	18 650	1	*2*	1	*2*	1	*2*	
Mother's age, mean (*SD*)	18 698	21.6	*4.6*	21.8	*4.6*	21.7	*4.6*	.005^e^
Mother's age at marriage, mean (*SD*)	16 114	15.2	*2.0*	15.3	*2.1*	15.2	*2.1*	.118^e^
Mother's age at first pregnancy, mean (*SD*)	18 261	17.5	*2.6*	17.5	*2.7*	17.5	*2.6*	.776^e^
Asset score, mean (*SD*)	18 446	−0.07	*1.87*	0.03	*1.90*	−0.02	*1.89*	<.001^e^
Landholding ha, mean (*SD*)	16 861	0.46	*0.96*	0.47	*0.95*	0.47	*0.96*	0.277^e^

Characteristics of children with one or more measures each	*n* ^a^	Female		Male		Total		*p*
Age of child at measurement in months, mean (*SD*)	26 104	6.53	*6.00*	6.73	*6.02*	6.63	*6.01*	.007^e^
Total child measures		12 373		13 731		26 104		

Characteristics of mothers with one or more measures each	*n* ^a^	Female		Male		Total		*p*
Gestational age at pregnancy measurement, mean (*SD*)	5551	27.9	*7.6*	27.7	*7.5*	27.8	*7.5*	.340^e^
Months since birth at postpartum measurement, mean (*SD*)	15 807	8.3	*6.0*	8.5	*6.0*	8.4	*6.0*	.060^e^
Total number of maternal measures		10 024		11 298		21 322		

a *n* is for child &/or mother measures; *p* represents the *p‐*value for the difference between mothers of boys and girls ascertained by:

b One‐sample test of proportion for proportions of boys and girls.

c Pearson's chi squared test for categorical variables.

d Parity = total previous live and still births.

e *t*‐tests for continuous variables.

In a study on Rhesus macaques, Hinde found that differences between sons and daughters were larger for primiparous females and suggested that this may be due to fewer bodily resources to fuel lactation (Hinde, 2009). We therefore conducted sensitivity analyses for the anthropometric outcomes, using primigravida women only, to see if differences by child sex were larger in this group.

Since we expected that the nutritional deficit in boys compared to girls would be larger when the mother is undernourished, we conducted sensitivity analyses by maternal nutritional status, for anthropometric *z*‐scores (LAZ, WLZ, and HCAZ) at 0 to 8 days. We limited these analyses to the first 8 days of life since the nutritional status of the newborn is almost entirely attributable to the nutrients gained in utero at this time, whereas nutritional status at older ages is also influenced by other factors. We fitted separate models for children of (a) all mothers and of mothers who were (b) short stature (<145 cm), (c) ≥145 cm height, (d) underweight (<18.5 kg/m^2^), and (e) not underweight ( ≥18.5 kg/m^2^ BMI). To test for differential effects of sex by maternal nutritional status, we also fitted models for LAZ, WLZ, and HCAZ using all available cases from 0 to 8 days with interaction terms [child sex * maternal underweight (BMI <18.5 kg/m^2^)] or [child sex * short stature (height <145 cm)].

To test whether the difference between boys and girls increases with increased access to food, we fitted multilevel mixed‐effects linear regression models adjusted for covariate set 1 in each study arm separately, restricting these analyses to anthropometry in the first 8 days after birth when infant size is most readily attributable to nutritional status of the mother. To test whether pregnancy energy intake increased in the supplementation arm in mothers of boys more than girls, we similarly fitted separate regression models with maternal energy intake in late pregnancy as the dependent variable and child sex as the exposure (adjusted for covariate set 2).

All analyses were undertaken using Stata version 16.1 (StataCorp. 2017. Stata Statistical Software: Release 16. College Station, TX: StataCorp LLC).

## 3 RESULTS

Respondent characteristics are summarized by child sex in Table 1 and further details are provided in Table S1. A participant flow diagram is provided in Figure S1. Our study population was largely Hindu, with 16% Muslims. Subsistence farming of rice, wheat, lentils, and other pulses made up the livelihood of most of the population. Sources of staple food included own production (63%), sharecropping (24%), labor exchange (being paid for labor in food) (31%), and purchase (79%). People reside in densely populated villages in with poor sanitation (75% of households had no toilet) and high levels of indoor pollution from burning dung and agricultural residues for cooking (95% of households). Two‐thirds lived in houses constructed from timber, sticks, and mud plaster, mostly with roofs made of tiles (70%) or thatch (10%). Twenty‐nine percent lived in brick and cement structures, with cement roofs (20%). Most households had access to electricity (92%).

Two‐thirds of mothers and half the husbands had no education, while only 14% of mothers and 24% of husbands reached secondary education. Overseas labor migration was common (45% of households). On average, mothers had married at 15 years, well below the legal age of 18 years in Nepal. On average, they also had their first pregnancy early, at 17.5 years. The sex of children born of the trial was skewed toward boys (53%). Seasons of pregnancy postpartum and child measurements, and gestational age at measurement of mother did not differ by child sex (Table S1).

All sex differences below are reported as the coefficient for males, relative to females. Plots of analyses are presented in the main results section while descriptive data and regression outputs are tabulated in the Supporting Information.

Hypothesis 1 Sex differences in children's absolute anthropometry and *z*‐scores.

**FIGURE 2:**
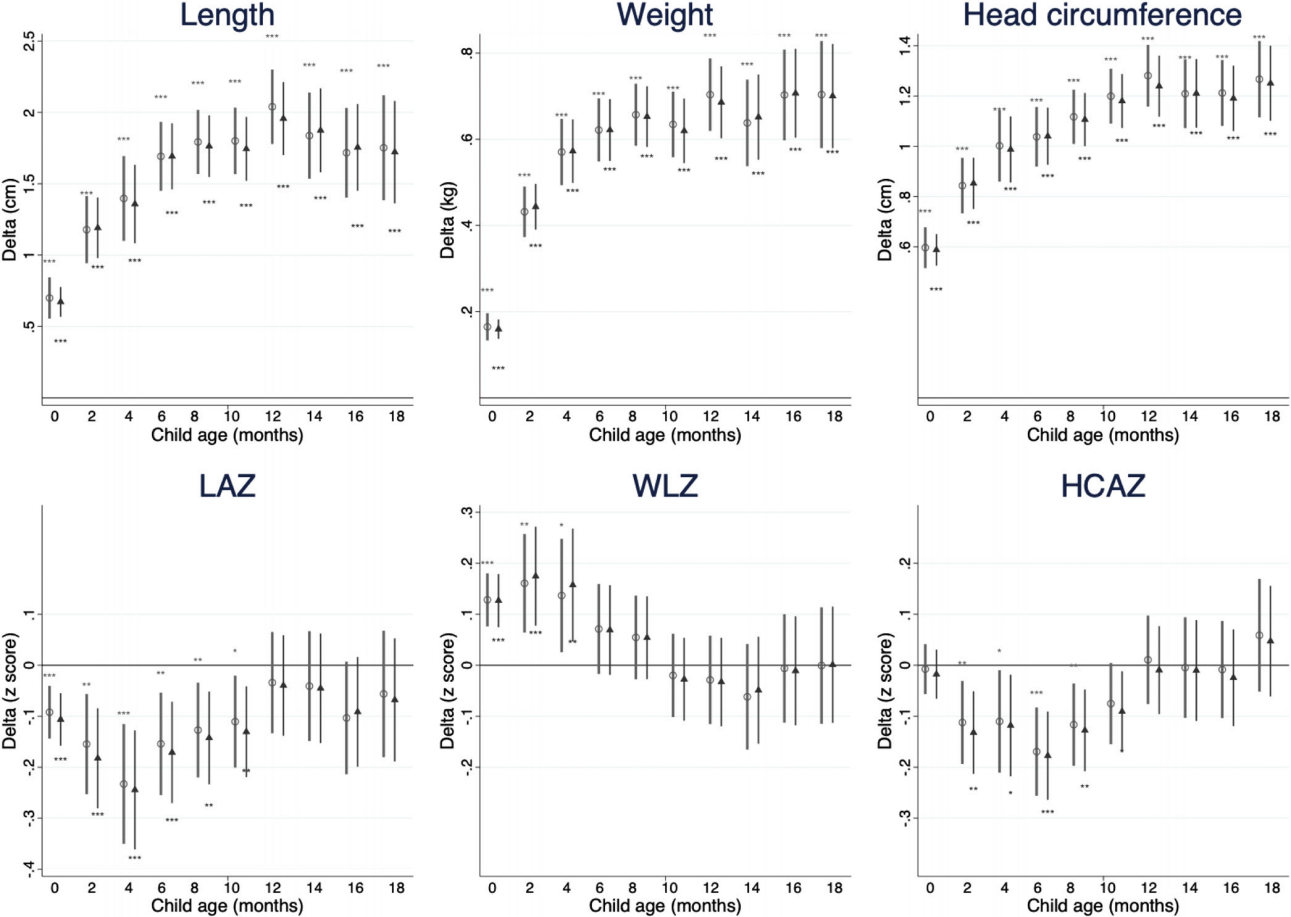
Sex differences in anthropometry and anthropometric *z*‐sores in children 0–20 months in 2 monthly‐categories. Delta values are coefficient for males relative to females as the reference category, obtained from mixed‐effects linear regression models adjusted for study cluster as a random effect. HCAZ, head circumference‐for‐age *z‐*score; LAZ, length‐for‐age *z‐* score; WLZ, weight‐for‐length *z‐*score. Circles represent unadjusted coefficients, triangles represent coefficients adjusted for age of child, mother's parity, education and asset quintile, study arm of trial and randomization strata. Age categories: 0–1.9, 2–3.9, 4–5.9, 6–7.9, 8–9.9, 10–11.9, 12–13.9, 14–15.9, 16–17.9, 18–19.9 months. Asterisks shown above unadjusted and below adjusted coefficient plots indicate: *p < .05; **p < .01; ***p < .001. A table of regression results including sample sizes (*n*) is provided in Table S2

Unadjusted and adjusted multi‐level mixed‐effects regression coefficients for male anthropometric outcomes and *z*‐scores in 2‐monthly age categories are presented in Figure 2 and Table S2 and, for children of primigravidae, in Figure S2 and Table S4. As stated in H_0_1, boys had larger absolute anthropometric values (length, weight and head circumference) than girls in all 2‐month age intervals. The difference between the sexes increased over the first year and then slightly decreased or stayed similar between 12 and 20 months.

As stated in H_0_1, boys had lower LAZ and HCAZ than girls throughout infancy up to 12 months. Adjusted analyses showed that in early life <2 months, boys had −0.11 (95% CI ‐0.16, −0.05) lower LAZ, but this difference increased to −0.24 (95% CI −0.36, −0.13) by 4 to 6 months. From 6 months, boys caught up such that there was no sex difference from 12 months onwards. HCAZ showed a similar pattern except that boys were not different from girls at 0 to 2 months. Adjusted analyses showed that boys' HCAZ reached a maximum difference of −0.18 (95% CI −0.26, −0.09) at 6 to 8 months, but thereafter they began to catch up such that there was no difference between 12 and 18 months. WLZ showed an inverse pattern to that of LAZ, failing to support the hypothesis that boys have lower WLZ. Boys had higher WLZ from birth to 6 months with a maximum adjusted difference of 0.17 (95% CI 0.78, 0.27) at 2 to 4 months. This difference gradually faded so that the boys had similar WLZ from 10 months onwards.

Hypothesis 2 Sex differences in stunting and wasting.

**FIGURE 3:**
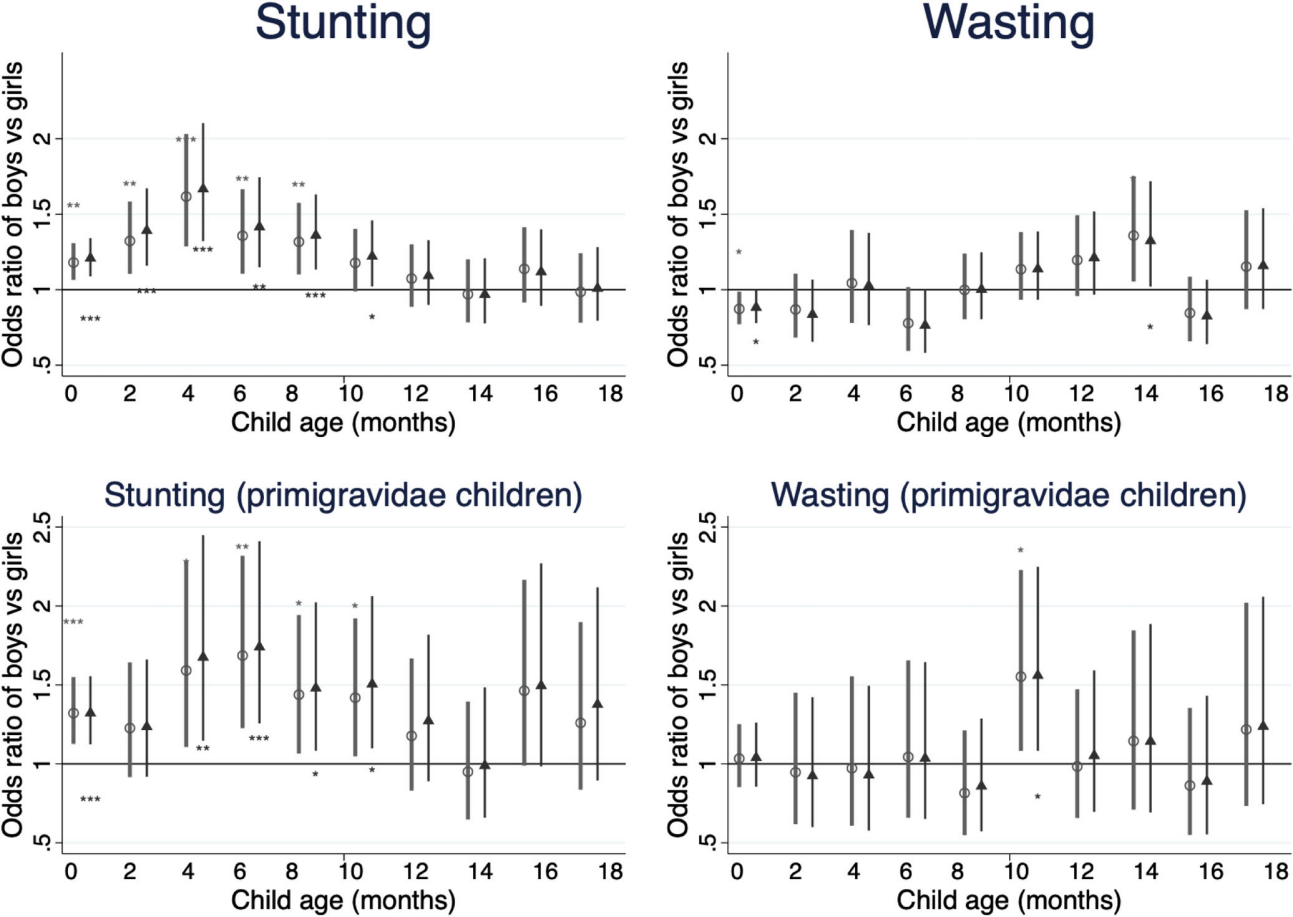
Odds ratio for stunting and wasting in boys relative to girls in 2‐month age categories in children 0 to 20 months of age of all mothers (top) and of primigravidae mothers only (bottom). All coefficients come from mixed‐effects logistic regression models adjusted for study cluster as a random effect. Circles represent unadjusted coefficients, triangles represent coefficients adjusted for age of child, mother's education and asset quintile, study arm of trial and randomization strata. In regressions with all cases adjusted analyses are also adjusted for maternal parity. Asterisks shown above unadjusted and below adjusted coefficient plots indicate: *p < .05; **p < .01; ***p < .001. Age categories 0–1.9, 2–3.9, 4–5.9, 6–7.9, 8–9.9, 10–11.9, 12–13.9, 14–15.9, 16–17.9, 18–19.9 months. A table of regression results including sample sizes (*n*) is provided in Table S3

Odds ratios for stunting and wasting in boys relative to girls by age are shown in Figure 3 and Table S3, for all cases and for children of primigravidae only. Results support H_0_2, in showing that sons are at greater risk of stunting in the first year of life, but did not show that risk of wasting is consistently higher in sons. The odds of stunting in boys relative to girls started 21% higher in boys at 0–2 months (adjusted OR [aOR] 1.209, 95% CI 1.089, 1.342) and increased to a 67% higher at 4 to 6 months (aOR 1.667 [95% CI 1.321, 2.103]). Between 6 and 12 months the higher odds for boys gradually fell such that they did not differ from girls between 12 and 20 months. Wasting showed a less clear pattern but, analyzing all cases, boys had 12% lower odds at birth (aOR 0.882 (95% CI 0.779, 0.999) and higher odds at 14 to 16 months (aOR 1.324 (95% CI 1.021, 1.719). There was no difference in wasting between boys and girls at any other time point.

On average, from birth to 4 months children of primigravidae (Table S4) were slightly shorter (42.5 cm vs. 42.9 cm) with lower weight (3.23 vs. 3.30 kg) and head circumference (35.97 vs. 36.18 cm) compared to children in the whole sample (Table S2). From 4 months onwards, children of primigravidae grew more rapidly catching up to be slightly larger or similar to all children by 12 to 13.9 months (length 73.38 vs. 73.26 cm, and weight 8.33 vs. 8.24 kg in children of primigravidae vs. all children). Sex differences in the odds of stunting in the first 20 months were broadly similar in first‐born children but the confidence intervals were wider due to the smaller sample sizes (Figure 3 and Table S3). At birth to 2 months though, where a large sample (*n* = 3135) was available, sons of primigravidae were had 32% higher odds of stunting than daughters (aOR 1.322 (95% CI 1.124, .1555), suggesting that, as stated in H_0_3, boys' growth deficit in utero is greater in primigravidae. However, wasting did not show a clear pattern over time in firstborns. The odds of wasting were higher in boys at 10 to 12 months (aOR 1.560 (95% CI 1.082, 2.249) but there were no differences at any other timepoints in the first 20 months.

Hypothesis 3 Sex differences in neonatal size when mothers are undernourished (low stature or low BMI) or not.

**FIGURE 4:**
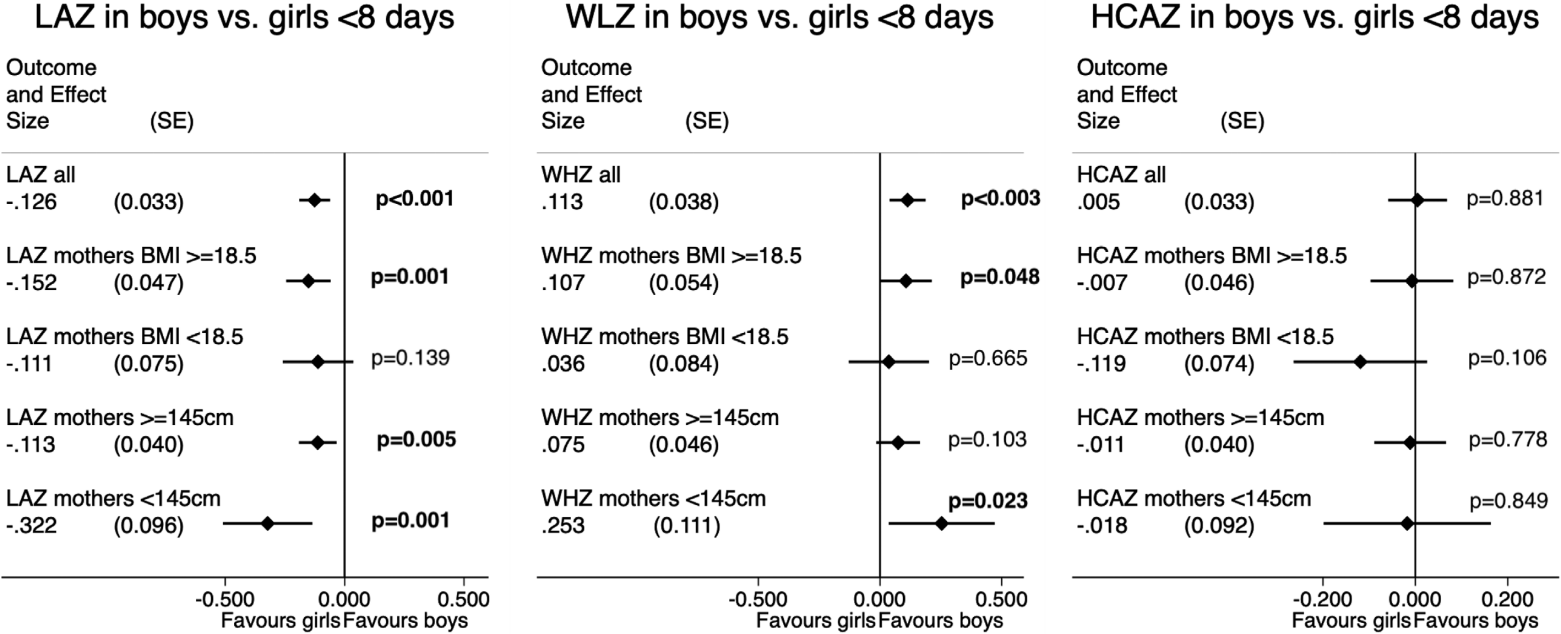
Adjusted coefficients for anthropometric *z‐*scores at 0–8 days in boys relative to girls born to all mothers, with the results further stratified by groups of maternal BMI (underweight vs. not) and stature (short vs. not). All coefficients come from separate mixed‐effects logistic regression models adjusted for study cluster as a random effect and for age of child, mother's parity, education and asset quintile, study arm of trial and randomization strata as fixed effects. HCAZ, head circumference‐for‐age; LAZ, length‐for‐age; WLZ, weight‐for‐length. Maternal underweight defined as BMI < 18.5 kg/m^2^; short stature as <145 cm. *p*‐values are indicated near the plotted coefficient. A table of regression results including sample sizes (*n*) is provided in Table S5

Figure 4 provides adjusted *z*‐score coefficients of boys relative to girls in the first 8 days from five separate regression models including children of a) all mothers, mothers who were b) underweight, c) not underweight, d) short stature and e) not short stature. Boys had lower adjusted LAZ than girls in all subgroups. The difference was −0.15 in not underweight and −0.11 in underweight mothers. As stated in H_0_3, the coefficient for LAZ of boys relative to girls born to short women (<145 cm) was more negative (−0.32) than in children of not short women (−0.11) (Table S5).

Adjusted analyses showed that in the first 8 days of life, boys had 0.11 (95% CI 0.04, 0.19) greater WLZ in the whole sample, 0.11 (95% CI 0.00, 0.21) greater WLZ in not‐underweight mothers, and 0.25 (95% CI 0.04, 0.471) greater WLZ in short mothers, providing mixed support for H_0_3 that differences are larger in children of undernourished mothers. Other findings on WLZ and HCAZ did not confirm H_0_3. Boys' WLZ did not differ from girls in underweight and not‐short mothers. The higher WLZ in sons than daughters of short mothers is explained by their lower LAZ values. HCAZ at 0 to 8 days did not differ between boys and girls in any of the maternal nutrition subgroups. Interaction terms for the effect of sex by maternal underweight (BMI <18.5) or short stature (height < 145 cm) upon WLZ and HCAZ were not statistically significant. However, for LAZ, significant interaction terms for male X maternal short stature in adjusted and unadjusted models indicated that the deficit in LAZ in boys compared with girls was greater in children of mothers that were themselves stunted compared with children of mothers >=145 cm tall (Figure S4 and Table S5).

Hypothesis 4 Maternal pregnancy and postpartum nutritional status by offspring sex.

**FIGURE 5:**
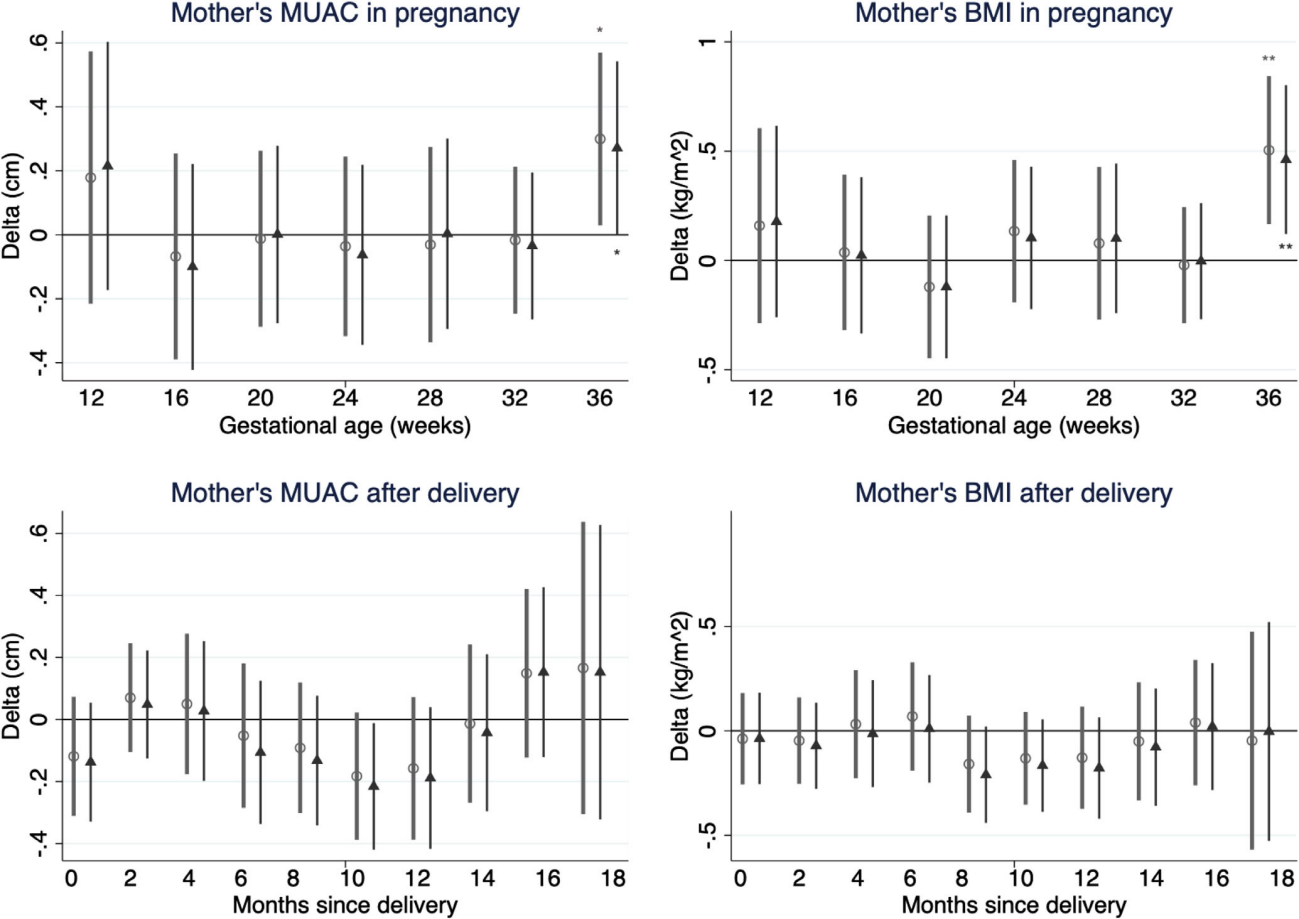
Coefficients comparing mothers of boys with mothers of girls (as the reference category) in terms of mid‐upper arm circumference (MUAC) and body mass index (BMI) in 4‐weekly intervals from 12 to 40 weeks' gestation of pregnancy and in 2‐monthly intervals from 0 to 20 months after delivery. All coefficients come from mixed‐effects linear regression models adjusted for study cluster as a random effect. Circles represent unadjusted coefficients, triangles represent coefficients adjusted for gestational age (in pregnancy models) or age of child (in postpartum models), mother's parity, education and asset quintile, study arm of trial and randomization strata. Gestational age categories 12–15.9, 16–19.9, 20–23.9, 24–27.9, 28–31.9, 32–35.9, 36–39.9 weeks. Postpartum time since delivery categories 0–1.9, 2–3.9, 4–5.9, 6–7.9, 8–9.9, 10–11.9, 12–13.9, 14–15.9, 16–17.9, 18–19.9 months. Asterisks shown above unadjusted and below adjusted coefficient plots indicate: *p < .05; ** *p* < .01. Coefficients at 36–39.9 weeks. *p*‐values were ≥ .05 for all other coefficients plotted. A table of regression results including sample sizes (*n*) is provided in Table S6

Overall, women in this population had a mean MUAC of 23.5 cm in pregnancy and BMI of 20 kg/m^2^ and MUAC of 23.6 cm at 0–20 months postpartum. Differences in maternal anthropometry by child sex are presented in Figure 5, with numerical values in Table S6.

Maternal anthropometry (BMI and MUAC) did not differ by child sex between 12 and 36 weeks' gestation nor at any of the postpartum time periods. However, adjusted analyses showed that in the last month of pregnancy (36 to 40 weeks), mothers of sons had 0.27 (95% CI 0.00, 0.54) cm higher MUAC and 0.46 (95% CI 0.12 0.80) kg/m^2^ higher BMI than mothers of daughters. This provides weak evidence to support H_0_4 in very late pregnancy only, but no evidence that mothers of sons are larger earlier in pregnancy or thinner during the postpartum period.

When primigravidae mothers only were analyzed (Figure S3 and Table S7) the patterns were similar to those found in all mothers (Figure 5). Only in a few cases were the magnitudes of the differences larger among primigravidae than in models with all women, providing weak evidence in support of H_0_3.

Hypothesis 5 Maternal energy and micronutrient consumption in pregnancy by offspring sex.

Mothers' dietary diversity was on average low, at 4.4 food groups out of 10, with only 46% consuming 5 or more food groups in the preceding 24 h. On average, women consumed 1.7 main meals and 1.7 snacks per day. Energy and micronutrient intakes were below requirements and mean probability of adequacy (MPA) was only 45% (SD 20%) (Table 2).

**TABLE 2:** Unadjusted and adjusted coefficients comparing mothers dietary intake of mothers of boys with mothers of girls in terms of energy and iron intake and mean probability of adequacy of 11 micronutrients

	Energy intake kcal/d^a^ ^,^ ^b^	Iron intake mg/d^a^ ^,^ ^b^	Mean probability of adequacy (MPA)^c^ (proportion)
Female mean	2150	14.8	0.458
*SD*	523	3.6	0.202
*n*	212	212	212
Male mean	2167	14.6	0.449
*SD*	557	3.6	0.204
*n*	258	258	258
Total mean	2159	14.7	0.453
*SD*	541	3.6	0.203
*n*	470	470	470
Unadjusted coeff.	5.5	−0.164	−0.009
*95% CI*	−88.0, 99.1	−0.787, 0.460	−0.044, 0.026
*p*‐value	.908	.607	.617
*n*	470	470	470
Adjusted coeff.	23.498	−0.021	−0.004
95% CI	−66.3, 113.3	−0.625, 0.583	−0.037, 0.030
*p*‐value	.608	.945	.826
*n*	457	457	457

a Adjusted for gestational age, mother's parity, education and asset quintile, study arm of trial and randomization strata as fixed‐effects and study cluster as a random effect.

b Analyses using data from 3 days of 24‐h quantitative dietary recall in pregnant women 25 to 42 weeks' gestation.

c MPA averages the probability of adequacy for 11 micronutrients including calcium, vitamin C, vitamin A, vitamins B1, B2, B3, B6, B9, B12, iron and zinc.

**FIGURE 6:**
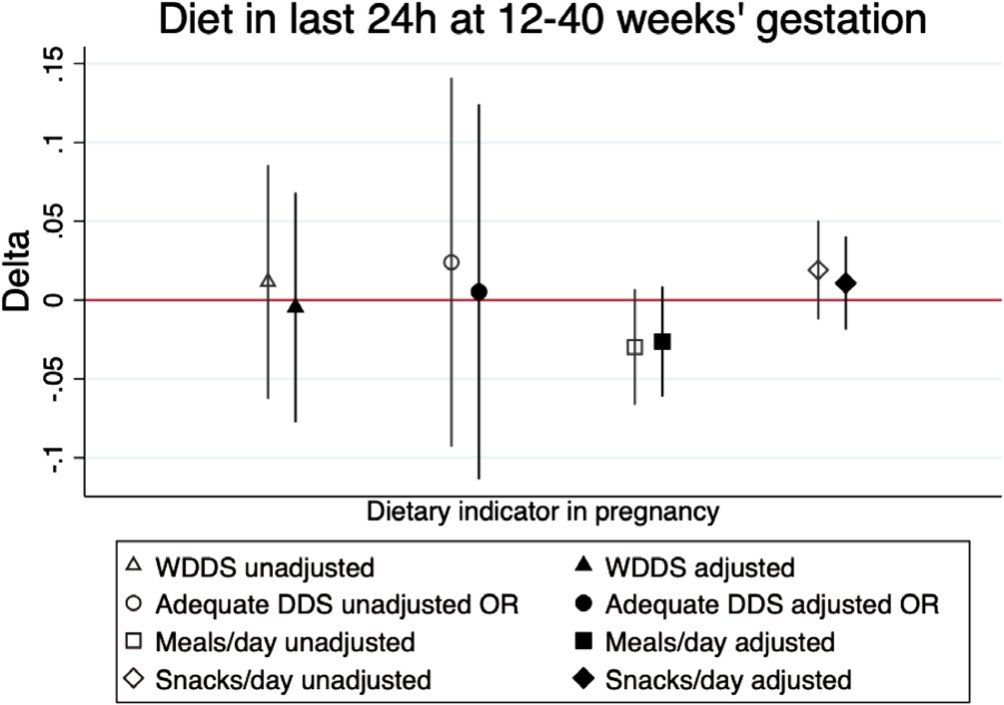
Coefficients comparing diet of mothers in the preceding 24 h in terms of women's dietary diversity score (WDDS), adequate dietary diversity (WDDS >=5), main meals/day and snacks/day (at 12 to 40 weeks gestational age). All coefficients come from mixed‐effects linear regression models adjusted for study cluster as a random effect. Hollow shapes represent unadjusted coefficients, filled shapes represent coefficients adjusted for gestational age, mother's parity, education and asset quintile, study arm of trial and randomization strata. *p*‐values were ≥ .05 for all coefficients plotted. A table of regression results, including coefficients and sample sizes from separate regressions in different gestational age groupings, is provided Table S8

Contrary to H_0_5, that mothers of sons would consume more energy and micronutrients, we found that diets of mothers of boys during pregnancy did not differ from mothers of girls, whether measured as meals or snacks per day, dietary diversity score, odds of adequate dietary diversity (Figure 6 and Table S8), energy intakes, dietary iron intakes, or probability of micronutrient adequacy (Table 2). This was true whether all available data were analyzed together and when 4‐weekly gestational age intervals were analyzed.

Hypothesis 6 Sex‐differences in neonatal body size by trial study arm.

**FIGURE 7:**
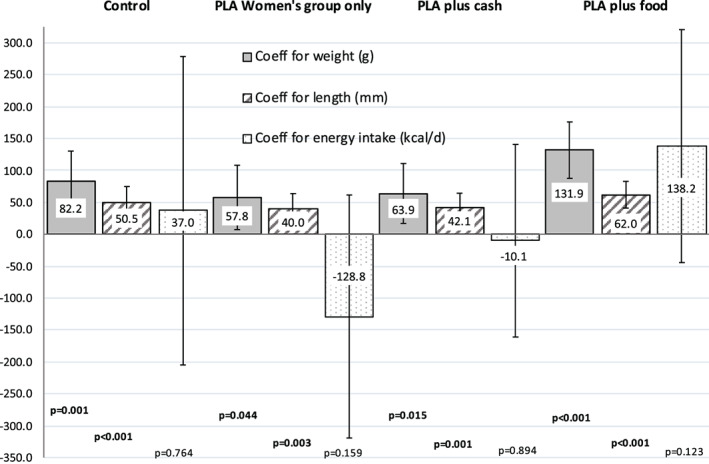
Adjusted regression^†^ coefficients from separate regression analyses in each of four study arms for boys versus girls for newborn weight (g), length (mm) at 0–7 days, and energy intake (kcal/day) of mothers of sons relative to mothers of daughters in pregnancy^§^. ^†^Multilevel mixed‐effects linear regression models adjusted for infant age, parity, education, wealth, strata as fixed effects and with cluster as a random effect. Coefficients comparing boys with girls (or mothers of sons with mothers of daughters) with female as the reference group. Error bars indicate 95% confidence intervals for adjusted coefficients and p values are provided below each coefficient plotted. ^§^Mothers' usual energy intakes taken from three 24‐h quantitative dietary recalls during the 2015 monsoon in late pregnancy (mean 36.3, range 25–42 weeks). A table of regression results including sample sizes is provided Table S9

Figure 7 plots the adjusted coefficients for boys' relative to girls' neonatal weight and length, and third‐trimester energy intakes for mothers of boys relative to mothers of girls, from multilevel mixed‐effects regression models fitted separately for each study arm. Data are provided in Table S9. The difference in length of boys versus girls was in the range of 0.40 to 0.51 cm in the control, PLA only and PLA plus cash arms, and was 0.62 (95% CI 0.40, 0.84) cm in the PLA plus food arm. The greater weight of boys relative to girls was possibly enhanced in the food arm (131.9 g, 95% CI 85.9, 177.9) compared to the other arms, where the sex difference ranged from 58 to 82 g. This provides weak evidence to support H_0_6 that increased food intake in pregnancy is associated with larger sex differences in infant body size. However, in the small sample available, mothers' energy intakes in pregnancy generally did not differ between boys and girls in any of the study arms.

## 4 DISCUSSION

Over the first year of life in a rural, low‐income setting, we found sons to be persistently larger than daughters in absolute length, weight, and head circumference. This means that sons impose higher “growth costs” on their mothers through pregnancy and during breastfeeding. In theory, it is possible that sons might compensate for their higher costs of growth by reducing other components of energy expenditure, but evidence for this is currently lacking. Studies of physical activity in high‐ or middle‐income settings have reported either similar (Haisma et al., 2006) or higher activity and energy expenditure in male versus female infants (Campbell & Eaton, 1999) and measurements of total energy expenditure are greater in boys (Butte et al., 2000; World Health Organization et al., 2004). Despite their greater growth potential, boys' had higher odds of stunting during infancy, peaking at 4 to 6 months. Similarly, average LAZ and HCAZ were lower in boys than girls throughout infancy. Relative to WHO reference data, which incorporates the expectation of larger average size in boys in optimal conditions, boys therefore showed greater constraint of growth in length and HC. Although boys were less thin (higher WLZ) in the first 6 months, their lower average length‐for‐age z score indicates that boys maintain relative weight at the expense of linear growth in early infancy.

Sex differences in undernutrition have been widely reported in low and middle‐income countries (Andersen et al., 2013; Martorell & Zongrone, 2012; Schoenbuchner et al., 2019; Thurstans et al., 2020; Victora et al., 2008; Wamani et al., 2007; Young et al., 2018). Examination of the 2016 (Ministry of Health Nepal, New ERA, & ICF, 2017) Nepal DHS survey shows that, as in our study, stunting prevalence was generally higher in boys than girls. In our sample, the prevalence of wasting was higher in girls than boys at 0–2 months which is consistent with the 2016 Nepal DHS survey. However, in general across the world boys have higher prevalence of wasting (Thurstans et al., 2020). The heterogeneous findings across studies may reflect complex associations between stunting and wasting, different age ranges addressed by individual studies, or variable cultural factors relevant to the feeding of boys and girls (Thurstans et al., 2020). The tendency of wasted children to resolve their thinness by subsequently slowing their linear growth was recently reported in a large longitudinal analysis of Gambian infants (Schoenbuchner et al., 2019).

Given the greater growth potential of males, we expected that mothers of sons would have greater somatic nutritional status (BMI or MUAC), or increased nutritional intake, relative to mothers of daughters. Our findings did not support these hypotheses. The one exception to this pattern was that we observed greater maternal MUAC and BMI in mothers of sons in the last month of pregnancy. Some of this increase in BMI might be explained by the greater weight of the fetus and associated placental and amniotic tissues, however MUAC should be less confounded by this issue. Greater affluence of mothers of sons than daughters might explain some of this effect, however the effect remained when analyses were adjusted for wealth and education. Sampling variability might also have contributed, as the mothers measured in this time interval might randomly have comprised a heavier group, but this seems unlikely given the large numbers of mothers investigated at every time point. Hence, it is possible that mothers of sons do indeed gain more weight at the very end of pregnancy, and further research is needed to confirm this finding. Overall, there was little evidence of mothers having greater nutritional resources to provision sons during pregnancy and lactation, and this is consistent with the poorer growth of the sons compared to daughters. Our findings may therefore help explain the widespread greater susceptibility of males to undernutrition in early life, including in this population with its high prevalence of maternal undernutrition.

The sex difference in stunting peaked with 67% higher odds in boys than girls at 4–6 months and was largely resolved by 12 months. This indicates that boys catch up in length during the complementary feeding period and provides additional support for our interpretation that poorer growth among boys is a product of constrained maternal nutrition: once boys are no longer solely reliant upon their mothers they begin to make up their growth deficit.

The underlying reason for boys' relatively poor early growth in our setting may therefore lie in the fact that mothers of sons do not consume more food in pregnancy than mothers of daughters. In this setting, women are generally undernourished, as reflected by their low mean BMI value. The nutrient adequacy of their diet is lower than their male household head (Harris‐Fry, Paudel, Shrestha, et al., 2018) despite some improvements in equity of intake associated with trial interventions (Harris‐Fry, Paudel, Harrisson, et al., 2018). In contrast to observations of greater intake by mothers of sons in high‐income settings (Mathews et al., 2008; Tamimi et al., 2003), diets were similar in mothers of sons and daughters in our sample. The increases in BMI and MUAC in the final month of pregnancy suggest that there may have been higher energy intakes in late pregnancy in the main study sample, however, we were not able to detect any difference in energy intake, perhaps due to the small number of still‐pregnant women enrolled in the trial at study endpoint.

In our sample, mothers of sons were likely to be more affluent and have more children than mothers of daughters, as found elsewhere in Nepal (Pradhan et al., 2019). One possible explanation for this could be prevalence of female foeticide in this population. Some women who already have daughters and can afford it, may pay for sex determination and sex‐selective abortion of female fetuses (Pradhan et al., 2019). This may have contributed to the skewed birth sex ratio, with birth of sons being associated with women who are older, more affluent and educated and with more previous pregnancies and more children (Frost et al., 2013). However, another possible mechanism for the skewed sex ratio might be that offered by Trivers and Willard (1973), who proposed that natural selection favors mothers in better condition to bear sons (Trivers & Willard, 1973). At present we cannot distinguish between these explanations, and both might contribute. Regardless, our sex differences in growth persisted after adjustment for maternal wealth and education.

The overall undernourished status of mothers in this population is a marker of their lower status in the family, and indicative of inequitable household food allocation (Harris‐Fry, Paudel, Shrestha, et al., 2018). Of relevance here, we found that first‐time mothers had the lowest nutritional status, were younger and would likely have experienced greater inequity in the family hierarchy. As expected, we found that the sex‐differences in child growth were larger among these primigravidae, despite their children generally being smaller in absolute terms in the first months of life than those of multiparous women. Our findings therefore show that boys' growth is even more suppressed in this subgroup than in the wider population. Since adolescents may still be growing during pregnancy and lactation (Rah et al., 2008) the larger deficit in boys of primigravidae may be partially explained by the competition for energy and nutrients between mothers and the babies.

When we compared sex differences in the growth of boys and girls born to mothers of different nutritional status, we found a larger LAZ deficit of sons compared to daughters of short women (<145 cm). Conversely, we found that the birth weight increment of boys relative to girls may have been slightly greater in the food supplementation arm of the study, which implies that the sex difference increases when maternal nutritional supply is increased. These results are consistent with Stinson's (1985) finding that males seem to show a greater response to nutritional supplementation in pregnancy and support her theory that males are less buffered than females against environmental stresses and stimuli during growth and development (Stinson, 1985).

Given that the sex difference in stunting evident in early infancy had resolved by 12 months of age, it is important to consider the implications of these growth patterns for short‐ and long‐term health outcomes. In the short‐term, males are well‐established to have greater mortality risk, and although wasting is the strongest predictor of mortality, stunting is also associated with substantially elevated mortality risk (Black et al., 2008) especially when concurrent with wasting (Myatt et al., 2018). Therefore, any inability of mothers to meet the higher growth costs of sons may reduce their short‐term survival chances. Indeed, given widespread reports of greater morbidity of male versus female infants, it is possible that growth is not the only biological function affected, and that immune defense is also compromised. These two responses may also be related, since both fat and lean mass contribute to immune function (Bartz et al., 2014; Bresnahan & Tanumihardjo, 2014). In the long‐term, it is well‐established that growth patterns in early life predict adult noncommunicable disease (NCD) risk, and development in the first 1000 days of life is particularly important (Barker et al., 1989; Barker et al., 1990; Hales et al., 1991). Moreover, catch‐up growth following early faltering has also been shown to shape NCD risk (Eriksson et al., 1999; Singhal, 2017). Therefore, poorer linear growth in late fetal life and early infancy may contribute to adult NCD risk, both by depleting homeostatic metabolic capacity and increasing metabolic load (Wells, 2018). In this context, the greater susceptibility of males to early undernutrition might contribute to overall shorter male life expectancy that is widely reported (Luy & Gast, 2014). Further work is needed to address this.

Our study has several major strengths. We analyzed a large cohort with detailed data on both mother and children throughout pregnancy and the first 20 months of postnatal life which enabled us to assess pregnancy accretion in mothers and growth trajectory in children, as well as diet and somatic status in mothers. Limitations include the variable levels of follow‐up, so that some mothers and children had more measures over time than others. Detailed 24‐h dietary recall data were only available for a small subsample of still‐pregnant women captured at the study endpoint. Although tests of multiple hypotheses increase the chance of Type I error, corrections for multiple comparisons are not needed, as we are not conducting pairwise comparisons. Also, our conclusions are based upon patterns of differences over time between boys and girls and mothers of boys versus mothers of girls, rather than based upon strict use of *p* < .05 as cut‐off. While we can detect some clear patterns from our data, we cannot infer causality for the higher levels of stunting in boys than girls.

## 5 CONCLUSION

Despite boys requiring more energy for growth due to being larger, mothers of sons show very little evidence of acquiring greater energy reserves during pregnancy and lactation compared to mothers of daughters, nor do they consume different diets. The only difference we detected that favored mothers of sons were a transient increment in MUAC and BMI in late pregnancy. We conclude that in populations prone to undernutrition, mothers of sons appear unable to satisfy the elevated energy costs of their offspring associated with their greater growth drive. This contributes to the penalty of under‐nutrition ultimately being paid more by sons than daughters.

## CONFLICT OF INTEREST

The authors declare no competing interests.

## AUTHOR CONTRIBUTIONS

JW and NS conceived the paper and the analyses. NS led data collection on the ground, conducted all the analyses, and wrote the first draft of the paper for publication. HHF designed and led the substudy on detailed dietary intake on the ground and analyzed all data from it. DSM managed the field data collection team in Nepal. All authors reviewed the manuscript, provided input, and approved the paper for publication.

## Supporting information


**Table S1.** Additional respondent characteristics of mothers of girls and boys and of girls and boys.

**Table S2.** Absolute means of length, weight, head circumference, LAZ, WLZ and HCAZ in girls and boys, and unadjusted and adjusted coefficients, 95% CIs and *p*‐values of differences between boys versus girls for these.

**Table S3.** Absolute percentage prevalence of stunting and wasting girls and boys, and unadjusted and adjusted Odds Ratios, 95% CIs and *p*‐values of differences between boys versus girls for these outcomes.

**Table S4.** Absolute means of length, weight, head circumference, LAZ, WLZ, and HCAZ in girls and boys born to primigravidae, and unadjusted and adjusted coefficients, 95% CIs and *p*‐values of differences between boys versus girls of primigravidae for these outcomes.

**Table S5.** Absolute means of LAZ, WLZ, and HCAZ in the first 7.9 days of life in girls and boys, and unadjusted and adjusted coefficients, 95% CIs and p values of differences between boys versus girls for these outcomes in children of all mothers, not underweight, underweight, not short and short mothers.

**Table S6.** Absolute means of MUAC, BMI, and weight in mothers of girls and boys in pregnancy and postpartum, and unadjusted and adjusted coefficients, 95% CIs, and *p*‐values of differences between mothers of boys versus girls for these outcomes.

**Table S7.** Absolute means of MUAC, BMI, and weight in primigravidae mothers of girls and boys in pregnancy and postpartum, and unadjusted and adjusted coefficients, 95% CIs, and *p*‐values of differences between mothers of boys versus girls for these outcomes.

**Table S8.** Absolute estimates of dietary diversity, adequate DDS, Main meals/d, and snacks in mothers of boys and girls and the coefficients/odds ratios for differences in these outcomes by child sex.

**Table S9.** Coefficients for boys weight and length at birth <=72 h relative to girls, and third‐trimester energy intakes for mothers of boys relative to girls, from separate multilevel mixed regression models by study arm.

**Figure S1.** Participant flow in the Low Birth Weight South Asia Trial showing how mixed longitudinal cohorts were prepared from available cases at different follow‐up timepoints.

**Figure S2.** Coefficients comparing boy with girl children of primigravidae mothers only in terms of length, weight, head circumference, and *z*‐scores of length‐for‐age, weight‐for‐length, and head circumference‐for‐age in children 0 to 20 months of age in 2‐monthly age categories.

**Figure S3.** Coefficients comparing primigravidae mothers of boys with primigravidae mothers of girls as the reference category in terms of mid‐upper arm circumference (MUAC) and Body Mass Index (BMI) in 4‐weekly intervals from 12 to 40 weeks' gestation of pregnancy and in 2‐monthly intervals from 0 to 20 months after delivery.

**Figure S4** Different effect of child being male relative to female (blue line) upon length‐for‐age z score in the first 8 days of life by whether the mother was >=145 cm (black line 1–1) or <145 cm (red line 2–2).

## Data Availability

The data that support the findings of this study are available from the corresponding author upon reasonable request.
