# Supplementary material for: Differences in maternal and early child nutritional status by offspring sex in lowland Nepal
Source: Am J Hum Biol. 2021 Jul 6;34(3):e23637. doi: 10.1002/ajhb.23637 (PMC12086752; doi:10.1002/ajhb.23637)
Supplement: Supplementary file 1 — Table S1. Additional respondent characteristics of mothers of girls and boys and of girls and boys. [file AJHB-34-e23637-s010.docx]

**Supplemental Table 1. Additional respondent characteristics of mothers of girls and boys and of girls and boys**

| **Characteristic of respondent** | ***n***^†^ | **Female** | | **Male** | | **Total** | | ***p*** |
| --- | --- | --- | --- | --- | --- | --- | --- | --- |
| **Family member migrated** | 16113 |  |  |  |  |  |  | 0.684^‡^ |
| Not migrated |  | 4214 | 55% | 4658 | 55% | 8872 | 55% |  |
| Yes, husband or other migrated for work/study |  | 3416 | 45% | 3825 | 45% | 7241 | 45% |  |
| **Access to electricity** | 18641 |  |  |  |  |  |  | 0.312^‡^ |
| No access to electricity |  | 694 | 8% | 719 | 7% | 1413 | 8% |  |
| Access electricity by ‘hooking’ (without paying) |  | 1197 | 14% | 1359 | 14% | 2556 | 14% |  |
| Accesses electricity from government supply or solar |  | 6905 | 79% | 7767 | 79% | 14672 | 79% |  |
| **Wall type of residence** | 18641 |  |  |  |  |  |  | 0.262‡ |
| Mud & stems, straw, wooden planks or metal sheets |  | 5801 | 66% | 6432 | 65% | 12233 | 66% |  |
| Cement & stems, mud & bricks or stones, or other |  | 441 | 5% | 545 | 6% | 986 | 5% |  |
| Cement and brick |  | 2554 | 29% | 2868 | 29% | 5422 | 29% |  |
| **Roof type of residence** | 18641 |  |  |  |  |  |  | 0.119‡ |
| Thatch |  | 930 | 11% | 978 | 10% | 1908 | 10% |  |
| Traditional tiles (kapada) |  | 3868 | 44% | 4292 | 44% | 8160 | 44% |  |
| Modern tiles/Asbestos/Metal/other |  | 2288 | 26% | 2536 | 26% | 4824 | 26% |  |
| Cement |  | 1710 | 19% | 2039 | 21% | 3749 | 20% |  |
| **Toilet facilities** | 18641 |  |  |  |  |  |  | **0.003**^‡^ |
| Not deprived |  | 2180 | 25% | 2626 | 27% | 4806 | 26% |  |
| Deprived of toilet |  | 6616 | 75% | 7219 | 73% | 13835 | 74% |  |
| **Own production is a main source of food** | 15948 |  |  |  |  |  |  | **0.014**^‡^ |
| Not a farmer |  | 2879 | 38% | 3049 | 36% | 5928 | 37% |  |
| Produces own food |  | 4664 | 62% | 5356 | 64% | 10020 | 63% |  |
| **Sharecropping is a main source of food** | 15948 |  |  |  |  |  |  | 0.070^‡^ |
| Not a sharecropper |  | 5808 | 77% | 6369 | 76% | 12177 | 76% |  |
| Sharecropper |  | 1735 | 23% | 2036 | 24% | 3771 | 24% |  |
| **Labour exchange is a main source of food** | 15948 |  |  |  |  |  |  | **0.004**^‡^ |
| Does not do labour exchange |  | 5100 | 68% | 5861 | 70% | 10961 | 69% |  |
| Earns food in labour exchange |  | 2443 | 32% | 2544 | 30% | 4987 | 31% |  |
| **Food purchase is a main source of food** | 15947 |  |  |  |  |  |  | **0.045**^‡^ |
| Does not purchase staple food |  | 1554 | 21% | 1841 | 22% | 3395 | 21% |  |
| Purchases at least some staple food |  | 5989 | 79% | 6563 | 78% | 12552 | 79% |  |
| **Fuel use** | 18625 |  |  |  |  |  |  | **0.007**^‡^ |
| Biomass (dung, firewood, agricultural residues) burner |  | 8391 | 96% | 9314 | 95% | 17705 | 95% |  |
| Gas or non-biomass burner |  | 394 | 4% | 526 | 5% | 920 | 5% |  |
| **Characteristics of children in the long dataset** | ***n***^†^ | **Female** | | **Male** | | **Total** | | ***p*** |
| **Child age at measurement** | 26104 |  |  |  |  |  |  | 0.126^‡^ |
| 0-9 months |  | 4360 | 35% | 4633 | 34% | 8993 | 34% |  |
| 2-9 months |  | 1374 | 11% | 1494 | 11% | 2868 | 11% |  |
| 4-9 months |  | 874 | 7% | 965 | 7% | 1839 | 7% |  |
| 6-9 months |  | 937 | 8% | 1102 | 8% | 2039 | 8% |  |
| 8-9 months |  | 1111 | 9% | 1185 | 9% | 2296 | 9% |  |
| 10-19 months |  | 1042 | 8% | 1247 | 9% | 2289 | 9% |  |
| 12-19 months |  | 823 | 7% | 972 | 7% | 1795 | 7% |  |
| 14-19 months |  | 637 | 5% | 751 | 5% | 1388 | 5% |  |
| 16-19 months |  | 653 | 5% | 733 | 5% | 1386 | 5% |  |
| 18-19 months |  | 562 | 5% | 649 | 5% | 1211 | 5% |  |
| **Total child measures** |  | **12373** |  | **13731** |  | **26104** |  |  |
| **Season**^#^ **of child's measurement** | 25927 |  |  |  |  |  |  | 0.323^‡^ |
| Winter: mid-Dec to mid-Mar |  | 1965 | 16% | 2114 | 16% | 4079 | 16% |  |
| Spring: mid-Mar to mid-Jun |  | 659 | 5% | 742 | 5% | 1401 | 5% |  |
| Monsoon: mid-Jun to mid-Sep |  | 6031 | 49% | 6822 | 50% | 12853 | 50% |  |
| Autumn: mid-Sep to mid-Nov |  | 3649 | 30% | 3945 | 29% | 7594 | 29% |  |
| **Characteristics of mothers in the long dataset** | ***n***^†^ | **Female** | | **Male** | | **Total** | | ***p*** |
| **Gestational age at measurement in 4 week categories** | 5551 |  |  |  |  |  |  | 0.093^‡^ |
| weeks 12 to 19 |  | 207 | 8% | 232 | 8% | 439 | 8% |  |
| weeks 16 to 19 |  | 312 | 12% | 304 | 11% | 616 | 11% |  |
| weeks 20 to 29 |  | 336 | 13% | 405 | 14% | 741 | 13% |  |
| weeks 24 to 29 |  | 422 | 16% | 476 | 16% | 898 | 16% |  |
| weeks 28 to 39 |  | 323 | 12% | 368 | 13% | 691 | 12% |  |
| weeks 32 to 39 |  | 589 | 22% | 675 | 23% | 1264 | 23% |  |
| weeks 36 to 39 |  | 467 | 18% | 435 | 15% | 902 | 16% |  |
| **Season**^#^ **of mother's measurement in pregnancy** | 5551 |  |  |  |  |  |  | 0.509^‡^ |
| Winter: mid-Dec to mid-Mar |  | 740 | 28% | 818 | 28% | 1558 | 28% |  |
| Spring: mid-Mar to mid-Jun |  | 314 | 12% | 376 | 13% | 690 | 12% |  |
| Monsoon: mid-Jun to mid-Sep |  | 872 | 33% | 914 | 32% | 1786 | 32% |  |
| Autumn: mid-Sep to mid-Nov |  | 730 | 27% | 787 | 27% | 1517 | 27% |  |
| **Season**^#^ **of mother's measurement during postpartum** | 15681 |  |  |  |  |  |  | 0.588^‡^ |
| Winter: mid-Dec to mid-Mar |  | 727 | 10% | 789 | 9% | 1516 | 10% |  |
| Spring: mid-Mar to mid-Jun |  | 133 | 2% | 156 | 2% | 289 | 2% |  |
| Monsoon: mid-Jun to mid-Sep |  | 4268 | 58% | 4944 | 59% | 9212 | 59% |  |
| Autumn: mid-Sep to mid-Nov |  | 2200 | 30% | 2464 | 29% | 4664 | 30% |  |
| **Time since delivery at time of mother’s measurement** | 15807 |  |  |  |  |  |  | 0.367^‡^ |
| 0-9 months |  | 945 | 13% | 1016 | 12% | 1961 | 12% |  |
| 2-9 months |  | 1149 | 16% | 1283 | 15% | 2432 | 15% |  |
| 4-9 months |  | 705 | 10% | 801 | 10% | 1506 | 10% |  |
| 6-9 months |  | 757 | 10% | 914 | 11% | 1671 | 11% |  |
| 8-9 months |  | 953 | 13% | 1005 | 12% | 1958 | 12% |  |
| 10-19 months |  | 911 | 12% | 1073 | 13% | 1984 | 13% |  |
| 12-19 months |  | 729 | 10% | 872 | 10% | 1601 | 10% |  |
| 14-19 months |  | 565 | 8% | 688 | 8% | 1253 | 8% |  |
| 16-19 months |  | 487 | 7% | 557 | 7% | 1044 | 7% |  |
| 18-19 months |  | 177 | 2% | 220 | 3% | 397 | 3% |  |
| **Total number of maternal measures** |  | **10024** |  | **11298** |  | **21322** |  |  |
