# Supplementary material for: Differences in maternal and early child nutritional status by offspring sex in lowland Nepal
Source: Am J Hum Biol. 2021 Jul 6;34(3):e23637. doi: 10.1002/ajhb.23637 (PMC12086752; doi:10.1002/ajhb.23637)
Supplement: Supplementary file 2 — Table S2. Absolute means of length, weight, head circumference, LAZ, WLZ and HCAZ in girls and boys, and unadjusted and adjusted coefficients, 95% CIs and p‐values of differences between boys versus girls for these. [file AJHB-34-e23637-s009.docx]

**Supplemental Table 2. Absolute means of length, weight, head circumference, LAZ, WLZ and HCAZ in girls and boys, and unadjusted and adjusted coefficients, 95% CIs and p values of differences between boys versus girls for these**

| **Raw measures †** | **Length (cm)** | | | | | | **Weight (kg)** | | | | | | **Head circumference (cm)** | | | | | |
| --- | --- | --- | --- | --- | --- | --- | --- | --- | --- | --- | --- | --- | --- | --- | --- | --- | --- | --- |
|  | **Female** | | | **Male** | | | **Female** | | | **Male** | | | **Female** | | | **Male** | | |
| Child age grouping | Mean | *SD* | *n* | Mean | *SD* | *n* | Mean | *SD* | *n* | Mean | SD | *n* | Mean | *SD* | n | Mean | *SD* | *n* |
| 0 to 1.9 months | 49.53 | *3.42* |  | 50.22 | *3.64* | 4,579 | 3.127 | *0.719* | 4,354 | 3.292 | 0.822 | 4,624 | 34.10 | *1.95* | 4,329 | 34.70 | *2.03* | 4,600 |
| 2 to 3.9 months | 56.86 | *3.20* | 1,369 | 58.05 | *3.47* | 1,480 | 4.827 | *0.751* | 1,374 | 5.258 | 0.855 | 1,493 | 37.53 | *1.51* | 1,370 | 38.38 | *1.64* | 1,486 |
| 4 to 5.9 months | 61.51 | *3.29* | 874 | 62.85 | *3.48* | 965 | 5.871 | *0.787* | 872 | 6.431 | 0.901 | 964 | 39.53 | *1.52* | 872 | 40.51 | *1.66* | 961 |
| 6 to 7.9 months | 64.41 | *2.70* | 932 | 66.09 | *2.84* | 1,099 | 6.423 | *0.84* | 934 | 7.043 | 0.838 | 1,097 | 41.00 | *1.37* | 931 | 42.05 | *1.38* | 1,096 |
| 8 to 10.9 months | 66.66 | *2.68* | 1,109 | 68.46 | *2.79* | 1,179 | 6.745 | *0.848* | 1,104 | 7.409 | 0.903 | 1,178 | 41.91 | *1.32* | 1,104 | 43.04 | *1.32* | 1,177 |
| 10 to 11.9 months | 68.69 | *2.75* | 1,038 | 70.50 | *2.91* | 1,241 | 7.032 | *0.853* | 1,019 | 7.671 | 0.97 | 1,216 | 42.48 | *1.30* | 1,033 | 43.68 | *1.34* | 1,240 |
| 12 to 13.9 months | 70.54 | *2.85* | 822 | 72.60 | *2.78* | 972 | 7.308 | *0.854* | 791 | 8.015 | 0.92 | 934 | 42.91 | *1.26* | 817 | 44.19 | *1.37* | 967 |
| 14 to 15.9 months | 72.33 | *2.96* | 636 | 74.18 | *2.78* | 749 | 7.644 | *0.911* | 594 | 8.288 | 0.925 | 684 | 43.36 | *1.28* | 630 | 44.57 | *1.31* | 746 |
| 16 to 17.9 months | 73.96 | *3.00* | 649 | 75.69 | *2.96* | 729 | 7.887 | *0.958* | 585 | 8.594 | 0.93 | 648 | 43.65 | *1.23* | 644 | 44.86 | *1.25* | 727 |
| 18 to 19.9 months | 75.55 | *3.25* | 557 | 77.31 | *3.25* | 645 | 8.232 | *1.038* | 492 | 8.938 | 1.038 | 574 | 44.08 | *1.27* | 555 | 45.35 | *1.40* | 643 |
| **All children 0 to 19.9 m** | **60.53** | ***9.88*** | **12,308** | **62.22** | ***10.38*** | **13,638** | **5.360** | ***2.034*** | **12,119** | **5.883** | **2.264** | **13,412** | **38.81** | ***4.19*** | **12,285** | **39.89** | ***4.46*** | **13,643** |
| **Unadjusted coefficients^#^** | **Length (cm)** | | | | | | **Weight (kg)** | | | | | | **Head circumference (cm)** | | | | | |
| Child age grouping | Unadjusted Coeff | *95% CI upper* | *95% CI lower* | *p* | *n* |  | Unadjusted Coeff | *95% CI upper* | *95% CI lower* | *p* | *n* |  | Unadjusted Coeff | *95% CI upper* | *95% CI lower* | *p* | *n* |  |
| 0 to 1.9 months | 0.70 | *0.56* | *0.84* | **<0.001** | 8,901 |  | 0.17 | *0.13* | *0.20* | **<0.001** | 8,978 |  | 0.60 | *0.52* | *0.68* | **<0.001** | 8,929 |  |
| 2 to 3.9 months | 1.18 | *0.94* | *1.41* | **<0.001** | 2,849 |  | 0.43 | *0.37* | *0.49* | **<0.001** | 2,867 |  | 0.84 | *0.73* | *0.95* | **<0.001** | 2,856 |  |
| 4 to 5.9 months | 1.40 | *1.10* | *1.69* | **<0.001** | 1,839 |  | 0.57 | *0.49* | *0.65* | **<0.001** | 1,836 |  | 1.00 | *0.86* | *1.15* | **<0.001** | 1,833 |  |
| 6 to 7.9 months | 1.69 | *1.45* | *1.93* | **<0.001** | 2,031 |  | 0.62 | *0.55* | *0.70* | **<0.001** | 2,031 |  | 1.04 | *0.92* | *1.16* | **<0.001** | 2,027 |  |
| 8 to 10.9 months | 1.79 | *1.57* | *2.02* | **<0.001** | 2,288 |  | 0.66 | *0.59* | *0.73* | **<0.001** | 2,282 |  | 1.12 | *1.01* | *1.23* | **<0.001** | 2,281 |  |
| 10 to 11.9 months | 1.80 | *1.57* | *2.03* | **<0.001** | 2,279 |  | 0.64 | *0.56* | *0.71* | **<0.001** | 2,235 |  | 1.20 | *1.09* | *1.31* | **<0.001** | 2,273 |  |
| 12 to 13.9 months | 2.04 | *1.78* | *2.30* | **<0.001** | 1,794 |  | 0.70 | *0.62* | *0.79* | **<0.001** | 1,725 |  | 1.28 | *1.16* | *1.40* | **<0.001** | 1,784 |  |
| 14 to 15.9 months | 1.84 | *1.54* | *2.14* | **<0.001** | 1,385 |  | 0.64 | *0.54* | *0.74* | **<0.001** | 1,278 |  | 1.21 | *1.07* | *1.35* | **<0.001** | 1,376 |  |
| 16 to 17.9 months | 1.72 | *1.40* | *2.03* | **<0.001** | 1,378 |  | 0.70 | *0.60* | *0.81* | **<0.001** | 1,233 |  | 1.21 | *1.08* | *1.34* | **<0.001** | 1,371 |  |
| 18 to 19.9 months | 1.75 | *1.39* | *2.12* | **<0.001** | 1,202 |  | 0.70 | *0.58* | *0.83* | **<0.001** | 1,066 |  | 1.27 | *1.12* | *1.42* | **<0.001** | 1,198 |  |
| **Adjusted coefficients** ^#^ | **Length (cm)** | | | | | | **Weight (kg)** | | | | | | **Head circumference (cm)** | | | | | |
| Child age grouping | Adjusted Coeff | *95% CI upper* | *95% CI lower* | *p* | *n* |  | Adjusted Coeff | *95% CI upper* | *95% CI lower* | *p* | *n* |  | Adjusted Coeff | *95% CI upper* | *95% CI lower* | *p* | *n* |  |
| 0 to 1.9 months | 0.67 | *0.57* | *0.78* | **<0.001** | 8,815 |  | 0.16 | *0.14* | *0.18* | **<0.001** | 8,891 |  | 0.59 | *0.53* | *0.65* | **<0.001** | 8,843 |  |
| 2 to 3.9 months | 1.19 | *0.98* | *1.40* | **<0.001** | 2,819 |  | 0.44 | *0.39* | *0.50* | **<0.001** | 2,837 |  | 0.85 | *0.75* | *0.96* | **<0.001** | 2,826 |  |
| 4 to 5.9 months | 1.36 | *1.08* | *1.63* | **<0.001** | 1,827 |  | 0.57 | *0.50* | *0.65* | **<0.001** | 1,824 |  | 0.99 | *0.86* | *1.12* | **<0.001** | 1,821 |  |
| 6 to 7.9 months | 1.69 | *1.46* | *1.92* | **<0.001** | 2,017 |  | 0.62 | *0.55* | *0.69* | **<0.001** | 2,017 |  | 1.04 | *0.93* | *1.15* | **<0.001** | 2,013 |  |
| 8 to 10.9 months | 1.76 | *1.55* | *1.98* | **<0.001** | 2,277 |  | 0.65 | *0.58* | *0.72* | **<0.001** | 2,270 |  | 1.11 | *1.00* | *1.21* | **<0.001** | 2,269 |  |
| 10 to 11.9 months | 1.74 | *1.52* | *1.97* | **<0.001** | 2,270 |  | 0.62 | *0.55* | *0.69* | **<0.001** | 2,226 |  | 1.18 | *1.07* | *1.29* | **<0.001** | 2,264 |  |
| 12 to 13.9 months | 1.96 | *1.70* | *2.21* | **<0.001** | 1,771 |  | 0.69 | *0.60* | *0.77* | **<0.001** | 1,703 |  | 1.24 | *1.12* | *1.36* | **<0.001** | 1,762 |  |
| 14 to 15.9 months | 1.87 | *1.58* | *2.17* | **<0.001** | 1,340 |  | 0.65 | *0.55* | *0.75* | **<0.001** | 1,234 |  | 1.21 | *1.07* | *1.35* | **<0.001** | 1,331 |  |
| 16 to 17.9 months | 1.76 | *1.45* | *2.06* | **<0.001** | 1,360 |  | 0.71 | *0.60* | *0.81* | **<0.001** | 1,216 |  | 1.19 | *1.06* | *1.32* | **<0.001** | 1,353 |  |
| 18 to 19.9 months | 1.72 | *1.36* | *2.08* | **<0.001** | 1,181 |  | 0.70 | *0.58* | *0.82* | **<0.001** | 1,048 |  | 1.25 | *1.10* | *1.40* | **<0.001** | 1,177 |  |
| **Raw measures †** | **Length-for-age *z* score (LAZ)** | | | | | | **Weight-for-length z score (WLZ)** | | | | | | **Head Circumference-for-age z score (HCAZ)** | | | | | |
|  | **Female** | | | **Male** | | | **Female** | | | **Male** | | | **Female** | | | **Male** | | |
| Child age grouping | Mean | *SD* | *n* | Mean | *SD* | *n* | Mean | *SD* | *n* | Mean | *SD* | *n* | Mean | *SD* | *n* | Mean | *SD* | *n* |
| 0 to 1.9 months | -1.11 | *1.22* | 4,301 | -1.21 | *1.31* | 4,565 | -0.79 | *1.21* | 4,059 | -0.66 | *1.26* | 4,343 | -1.02 | *1.20* | 4,289 | -1.03 | *1.22* | 4,551 |
| 2 to 3.9 months | -1.17 | *1.29* | 1,361 | -1.31 | *1.47* | 1,466 | -0.55 | *1.36* | 1,359 | -0.39 | *1.37* | 1,465 | -1.40 | *1.07* | 1,358 | -1.50 | *1.22* | 1,464 |
| 4 to 5.9 months | -1.07 | *1.22* | 863 | -1.31 | *1.35* | 946 | -0.70 | *1.17* | 867 | -0.57 | *1.28* | 956 | -1.48 | *1.08* | 870 | -1.59 | *1.12* | 938 |
| 6 to 7.9 months | -1.24 | *1.11* | 930 | -1.40 | *1.19* | 1,094 | -0.91 | *1.01* | 927 | -0.84 | *1.01* | 1,093 | -1.38 | *0.97* | 927 | -1.55 | *1.04* | 1,092 |
| 8 to 10.9 months | -1.45 | *1.05* | 1,107 | -1.57 | *1.21* | 1,177 | -1.15 | *0.97* | 1,099 | -1.10 | *1.02* | 1,169 | -1.44 | *0.97* | 1,103 | -1.54 | *1.00* | 1,172 |
| 10 to 11.9 months | -1.60 | *1.01* | 1,037 | -1.70 | *1.16* | 1,236 | -1.34 | *0.92* | 1,015 | -1.36 | *1.03* | 1,211 | -1.53 | *0.92* | 1,031 | -1.60 | *1.01* | 1,237 |
| 12 to 13.9 months | -1.73 | *1.04* | 820 | -1.76 | *1.10* | 970 | -1.43 | *0.87* | 789 | -1.45 | *0.95* | 931 | -1.63 | *0.89* | 815 | -1.62 | *0.97* | 960 |
| 14 to 15.9 months | -1.87 | *1.03* | 635 | -1.91 | *1.01* | 746 | -1.41 | *0.90* | 593 | -1.47 | *0.98* | 680 | -1.67 | *0.92* | 630 | -1.67 | *0.94* | 741 |
| 16 to 17.9 months | -1.98 | *1.02* | 647 | -2.08 | *1.07* | 727 | -1.46 | *0.94* | 581 | -1.46 | *0.96* | 643 | -1.74 | *0.88* | 643 | -1.75 | *0.92* | 724 |
| 18 to 19.9 months | -2.06 | *1.09* | 556 | -2.11 | *1.10* | 643 | -1.37 | *0.92* | 487 | -1.37 | *0.97* | 570 | -1.68 | *0.92* | 555 | -1.62 | *1.02* | 641 |
| **All children 0 to 19.9 m** | **-1.37** | ***1.20*** | **12,257** | **-1.49** | ***1.28*** | **13,570** | **-0.98** | ***1.15*** | **11,776** | **-0.91** | ***1.22*** | **13,061** | **-1.34** | ***1.09*** | **12,221** | **-1.40** | ***1.14*** | **13,520** |
| **Unadjusted coefficients**^#^ | **Length-for-age z score (LAZ)** | | | | | | **Weight-for-length z score (WLZ)** | | | | | | **Head Circumference-for-age z score (HCAZ)** | | | | | |
| Child age grouping | Unadjusted Coeff | *95% CI upper* | *95% CI lower* | *p* | *n* |  | Unadjusted Coeff | *95% CI upper* | *95% CI lower* | *p* | *n* |  | Unadjusted Coeff | *95% CI upper* | *95% CI lower* | *p* | *n* |  |
| 0 to 1.9 months | -0.092 | *-0.144* | *-0.040* | **0.001** | 8,866 |  | 0.128 | *0.076* | *0.180* | **<0.001** | 8,402 |  | -0.007 | *-0.056* | *0.041* | 0.764 | 8,840 |  |
| 2 to 3.9 months | -0.155 | *-0.253* | *-0.056* | **0.002** | 2,827 |  | 0.161 | *0.064* | *0.258* | **0.001** | 2,824 |  | -0.112 | *-0.194* | *-0.031* | **0.007** | 2,822 |  |
| 4 to 5.9 months | -0.233 | *-0.350* | *-0.115* | **<0.001** | 1,809 |  | 0.137 | *0.026* | *0.248* | **0.016** | 1,823 |  | -0.110 | *-0.211* | *-0.010* | **0.032** | 1,808 |  |
| 6 to 7.9 months | -0.154 | *-0.255* | *-0.054* | **0.003** | 2,024 |  | 0.071 | *-0.017* | *0.159* | 0.114 | 2,020 |  | -0.169 | *-0.256* | *-0.083* | **<0.001** | 2,019 |  |
| 8 to 10.9 months | -0.127 | *-0.220* | *-0.034* | **0.007** | 2,284 |  | 0.055 | *-0.027* | *0.137* | 0.192 | 2,268 |  | -0.116 | *-0.197* | *-0.036* | **0.005** | 2,275 |  |
| 10 to 11.9 months | -0.111 | *-0.201* | *-0.020* | **0.016** | 2,273 |  | -0.020 | *-0.102* | *0.062* | 0.633 | 2,226 |  | -0.075 | *-0.155* | *0.005* | 0.065 | 2,268 |  |
| 12 to 13.9 months | -0.034 | *-0.133* | *0.065* | 0.502 | 1,790 |  | -0.029 | *-0.115* | *0.058* | 0.516 | 1,720 |  | 0.011 | *-0.076* | *0.098* | 0.809 | 1,775 |  |
| 14 to 15.9 months | -0.041 | *-0.149* | *0.067* | 0.457 | 1,381 |  | -0.062 | *-0.165* | *0.042* | 0.242 | 1,273 |  | -0.005 | *-0.103* | *0.094* | 0.927 | 1,371 |  |
| 16 to 17.9 months | -0.103 | *-0.214* | *0.007* | 0.067 | 1,374 |  | -0.006 | *-0.112* | *0.100* | 0.910 | 1,224 |  | -0.008 | *-0.104* | *0.087* | 0.862 | 1,367 |  |
| 18 to 19.9 months | -0.056 | *-0.180* | *0.068* | 0.375 | 1,199 |  | -0.001 | *-0.115* | *0.114* | 0.992 | 1,057 |  | 0.059 | *-0.052* | *0.169* | 0.297 | 1,196 |  |
| **Adjusted coefficients** ^#^ | **Length-for-age z score (LAZ)** | | | | | | **Weight-for-length z score (WLZ)** | | | | | | **Head Circumference-for-age z score (HCAZ)** | | | | | |
| Child age grouping | Adjusted Coeff | *95% CI upper* | *95% CI lower* | *p* | *n* |  | Adjusted Coeff | *95% CI upper* | *95% CI lower* | *p* | *n* |  | Adjusted Coeff | *95% CI upper* | *95% CI lower* | *p* | *n* |  |
| 0 to 1.9 months | -0.106 | *-0.158* | *-0.055* | **<0.001** | 8,780 |  | 0.127 | *0.075* | *0.179* | **<0.001** | 8,317 |  | -0.018 | *-0.066* | *0.031* | 0.478 | 8,754 |  |
| 2 to 3.9 months | -0.182 | *-0.280* | *-0.084* | **<0.001** | 2,797 |  | 0.175 | *0.078* | *0.272* | **<0.001** | 2,794 |  | -0.132 | *-0.213* | *-0.051* | **0.001** | 2,794 |  |
| 4 to 5.9 months | -0.244 | *-0.361* | *-0.128* | **<0.001** | 1,797 |  | 0.157 | *0.047* | *0.268* | **0.005** | 1,811 |  | -0.118 | *-0.218* | *-0.019* | **0.020** | 1,796 |  |
| 6 to 7.9 months | -0.171 | *-0.270* | *-0.072* | **0.001** | 2,011 |  | 0.069 | *-0.019* | *0.157* | 0.123 | 2,007 |  | -0.177 | *-0.264* | *-0.091* | **<0.001** | 2,006 |  |
| 8 to 10.9 months | -0.142 | *-0.233* | *-0.051* | **0.002** | 2,273 |  | 0.054 | *-0.027* | *0.135* | 0.192 | 2,257 |  | -0.128 | *-0.208* | *-0.048* | **0.002** | 2,263 |  |
| 10 to 11.9 months | -0.130 | *-0.220* | *-0.041* | **0.004** | 2,264 |  | -0.027 | *-0.109* | *0.054* | 0.508 | 2,217 |  | -0.091 | *-0.170* | *-0.012* | **0.024** | 2,259 |  |
| 12 to 13.9 months | -0.040 | *-0.138* | *0.059* | 0.430 | 1,767 |  | -0.033 | *-0.120* | *0.054* | 0.458 | 1,698 |  | -0.010 | *-0.096* | *0.077* | 0.829 | 1,753 |  |
| 14 to 15.9 months | -0.045 | *-0.153* | *0.062* | 0.410 | 1,336 |  | -0.049 | *-0.154* | *0.056* | 0.360 | 1,229 |  | -0.010 | *-0.109* | *0.089* | 0.838 | 1,326 |  |
| 16 to 17.9 months | -0.091 | *-0.199* | *0.016* | 0.096 | 1,356 |  | -0.011 | *-0.118* | *0.096* | 0.843 | 1,207 |  | -0.024 | *-0.119* | *0.070* | 0.614 | 1,349 |  |
| 18 to 19.9 months | -0.068 | *-0.189* | *0.053* | 0.270 | 1,178 |  | 0.001 | *-0.113* | *0.115* | 0.987 | 1,039 |  | 0.047 | *-0.061* | *0.156* | 0.392 | 1,175 |  |

† for all available cases regardless of availability of covariates; ^#^ comparing boys with girls
