# Supplementary material for: Differences in maternal and early child nutritional status by offspring sex in lowland Nepal
Source: Am J Hum Biol. 2021 Jul 6;34(3):e23637. doi: 10.1002/ajhb.23637 (PMC12086752; doi:10.1002/ajhb.23637)
Supplement: Supplementary file 3 — Table S3. Absolute percentage prevalence of stunting and wasting girls and boys, and unadjusted and adjusted Odds Ratios, 95% CIs and p‐values of differences between boys versus girls for these outcomes. [file AJHB-34-e23637-s001.docx]

**Supplemental Table 3. Absolute percentage prevalence of stunting and wasting girls and boys, and unadjusted and adjusted Odds Ratios, 95% CIs and p values of differences between boys versus girls for these outcomes**

| **Raw measures for all available cases†** | **Stunting in all children** | | | | | | **Wasting in all children** | | | | | |
| --- | --- | --- | --- | --- | --- | --- | --- | --- | --- | --- | --- | --- |
|  | **Female** | | | **Male** | | | **Female** | | | **Male** | | |
| Child age grouping | Mean proportion | SD proportion | *n* | Mean proportion | SD proportion | *n* | Mean proportion | SD proportion | *n* | Mean proportion | SD proportion | *n* |
| 0 to 1.9 months | 0.204 | 0.403 | 4,301 | 0.233 | 0.423 | 4,565 | 0.151 | 0.358 | 4,079 | 0.133 | 0.340 | 4,377 |
| 2 to 3.9 months | 0.216 | 0.412 | 1,361 | 0.263 | 0.440 | 1,466 | 0.113 | 0.317 | 1,368 | 0.099 | 0.299 | 1,478 |
| 4 to 5.9 months | 0.188 | 0.391 | 863 | 0.271 | 0.445 | 946 | 0.114 | 0.317 | 872 | 0.117 | 0.322 | 964 |
| 6 to 7.9 months | 0.220 | 0.415 | 930 | 0.278 | 0.448 | 1,094 | 0.135 | 0.341 | 929 | 0.108 | 0.310 | 1,094 |
| 8 to 10.9 months | 0.284 | 0.451 | 1,107 | 0.342 | 0.474 | 1,177 | 0.175 | 0.380 | 1,102 | 0.175 | 0.380 | 1,172 |
| 10 to 11.9 months | 0.338 | 0.473 | 1,037 | 0.373 | 0.484 | 1,236 | 0.231 | 0.422 | 1,016 | 0.254 | 0.436 | 1,212 |
| 12 to 13.9 months | 0.405 | 0.491 | 820 | 0.421 | 0.494 | 970 | 0.232 | 0.422 | 790 | 0.264 | 0.441 | 934 |
| 14 to 15.9 months | 0.450 | 0.498 | 635 | 0.442 | 0.497 | 746 | 0.228 | 0.420 | 593 | 0.286 | 0.452 | 682 |
| 16 to 17.9 months | 0.490 | 0.500 | 647 | 0.517 | 0.500 | 727 | 0.299 | 0.458 | 581 | 0.266 | 0.442 | 644 |
| 18 to 19.9 months | 0.505 | 0.500 | 556 | 0.501 | 0.500 | 643 | 0.236 | 0.425 | 487 | 0.263 | 0.440 | 571 |
| **All children 0 to 19.9 months** | **0.279** | **0.448** | **12,257** | **0.317** | **0.466** | **13,570** | **0.172** | **0.377** | **11,817** | **0.170** | **0.376** | **13,128** |
| **Unadjusted Odds Ratios**^#^ | **Stunting in all children** | | | | | | **Wasting in all children** | | | | | |
| Child age grouping | Unadjusted Odds Ratio | *95% CI upper* | *95% CI lower* | *p* | *n* |  | Unadjusted Odds Ratio | *95% CI upper* | *95% CI lower* | *p* | *n* |  |
| 0 to 1.9 months | 1.180 | *1.065* | *1.308* | ***0.002*** | 8,866 |  | 1.033 | *0.853* | *1.252* | *0.738* | 8,456 |  |
| 2 to 3.9 months | 1.323 | *1.105* | *1.584* | ***0.002*** | 2,827 |  | 0.947 | *0.618* | *1.451* | *0.802* | 2,846 |  |
| 4 to 5.9 months | 1.617 | *1.287* | *2.031* | ***0.000*** | 1,809 |  | 0.972 | *0.608* | *1.555* | *0.906* | 1,836 |  |
| 6 to 7.9 months | 1.357 | *1.106* | *1.666* | ***0.004*** | 2,024 |  | 1.044 | *0.658* | *1.656* | *0.855* | 2,023 |  |
| 8 to 10.9 months | 1.317 | *1.101* | *1.576* | ***0.003*** | 2,284 |  | 0.815 | *0.548* | *1.212* | *0.313* | 2,274 |  |
| 10 to 11.9 months | 1.178 | *0.988* | *1.403* | *0.067* | 2,273 |  | 1.552 | *1.082* | *2.228* | ***0.017*** | 2,228 |  |
| 12 to 13.9 months | 1.074 | *0.886* | *1.300* | *0.467* | 1,790 |  | 0.983 | *0.657* | *1.472* | *0.935* | 1,724 |  |
| 14 to 15.9 months | 0.970 | *0.783* | *1.201* | *0.780* | 1,381 |  | 1.145 | *0.710* | *1.845* | *0.579* | 1,275 |  |
| 16 to 17.9 months | 1.138 | *0.915* | *1.414* | *0.245* | 1,374 |  | 0.863 | *0.550* | *1.354* | *0.521* | 1,225 |  |
| 18 to 19.9 months | 0.985 | *0.781* | *1.242* | *0.899* | 1,199 |  | 1.218 | *0.734* | *2.021* | *0.446* | 1,058 |  |
| **Adjusted Odds Ratios** ^#^ | **Stunting in all children** | | | | | | **Wasting in all children** | | | | | |
| Child age grouping | Adjusted Odds Ratio | *95% CI upper* | *95% CI lower* | *p* | *n* |  | Adjusted Odds Ratio | *95% CI upper* | *95% CI lower* | *p* | *n* |  |
| 0 to 1.9 months | 1.209 | *1.089* | *1.342* | ***0.000*** | 8,780 |  | 0.882 | *0.779* | *0.999* | ***0.049*** | 8,370 |  |
| 2 to 3.9 months | 1.392 | *1.159* | *1.671* | ***0.000*** | 2,797 |  | 0.836 | *0.655* | *1.067* | *0.150* | 2,816 |  |
| 4 to 5.9 months | 1.667 | *1.321* | *2.103* | ***0.000*** | 1,797 |  | 1.026 | *0.765* | *1.376* | *0.863* | 1,824 |  |
| 6 to 7.9 months | 1.416 | *1.149* | *1.745* | ***0.001*** | 2,011 |  | 0.763 | *0.582* | *1.002* | *0.051* | 2,010 |  |
| 8 to 10.9 months | 1.360 | *1.134* | *1.631* | ***0.001*** | 2,273 |  | 1.002 | *0.805* | *1.248* | *0.985* | 2,263 |  |
| 10 to 11.9 months | 1.221 | *1.021* | *1.459* | ***0.028*** | 2,264 |  | 1.137 | *0.934* | *1.386* | *0.201* | 2,219 |  |
| 12 to 13.9 months | 1.093 | *0.899* | *1.328* | *0.374* | 1,767 |  | 1.212 | *0.967* | *1.518* | *0.095* | 1,702 |  |
| 14 to 15.9 months | 0.969 | *0.777* | *1.208* | *0.778* | 1,336 |  | 1.324 | *1.021* | *1.719* | ***0.035*** | 1,231 |  |
| 16 to 17.9 months | 1.118 | *0.893* | *1.401* | *0.330* | 1,356 |  | 0.826 | *0.640* | *1.066* | *0.141* | 1,208 |  |
| 18 to 19.9 months | 1.009 | *0.795* | *1.282* | *0.940* | 1,178 |  | 1.159 | *0.872* | *1.540* | *0.311* | 1,040 |  |
| **Raw measures for primigravidae†** | **Stunting in children of primigravidae** | | | | | | **Wasting in children of primigravidae** | | | | | |
|  | **Female** | | | **Male** | | | **Female** | | | **Male** | | |
| Child age grouping | Mean proportion | SD proportion | *n* | Mean proportion | SD proportion | *n* | Mean proportion | SD proportion | *n* | Mean proportion | SD proportion | *n* |
| 0 to 1.9 months | 0.255 | 0.436 | 1,578 | 0.306 | 0.461 | 1,587 | 0.170 | 0.375 | 1,462 | 0.180 | 0.380 | 1,497 |
| 2 to 3.9 months | 0.301 | 0.459 | 492 | 0.320 | 0.467 | 493 | 0.099 | 0.299 | 495 | 0.090 | 0.290 | 496 |
| 4 to 5.9 months | 0.208 | 0.406 | 318 | 0.293 | 0.456 | 335 | 0.130 | 0.337 | 322 | 0.120 | 0.330 | 340 |
| 6 to 7.9 months | 0.216 | 0.412 | 380 | 0.317 | 0.466 | 426 | 0.103 | 0.304 | 380 | 0.110 | 0.310 | 427 |
| 8 to 10.9 months | 0.286 | 0.453 | 381 | 0.365 | 0.482 | 403 | 0.164 | 0.370 | 379 | 0.140 | 0.340 | 401 |
| 10 to 11.9 months | 0.313 | 0.465 | 351 | 0.390 | 0.488 | 423 | 0.174 | 0.380 | 339 | 0.250 | 0.430 | 410 |
| 12 to 13.9 months | 0.383 | 0.487 | 274 | 0.417 | 0.494 | 302 | 0.227 | 0.420 | 260 | 0.220 | 0.420 | 291 |
| 14 to 15.9 months | 0.435 | 0.497 | 209 | 0.423 | 0.495 | 220 | 0.209 | 0.408 | 191 | 0.230 | 0.420 | 202 |
| 16 to 17.9 months | 0.427 | 0.496 | 227 | 0.516 | 0.501 | 225 | 0.276 | 0.448 | 203 | 0.250 | 0.430 | 190 |
| 18 to 19.9 months | 0.460 | 0.500 | 200 | 0.509 | 0.501 | 212 | 0.253 | 0.436 | 174 | 0.280 | 0.450 | 189 |
| **All children 0 to 19.9 months** | **0.295** | **0.456** | **4,410** | **0.353** | **0.478** | **4,626** | **0.166** | **0.372** | **4,205** | **0.172** | **0.377** | **4,443** |
| **Unadjusted Odds Ratios** ^#^ | **Stunting in children of primigravidae** | | | | | | **Wasting in children of primigravidae** | | | | | |
| Child age grouping | Unadjusted Odds Ratio | *95% CI upper* | *95% CI lower* | *p* | *n* |  | Unadjusted Odds Ratio | *95% CI upper* | *95% CI lower* | *p* | *n* |  |
| 0 to 1.9 months | 1.321 | *1.127* | *1.549* | ***0.001*** | 3,209 |  | 1.033 | *0.853* | *1.252* | *0.738* | 3,003 |  |
| 2 to 3.9 months | 1.227 | *0.916* | *1.643* | *0.171* | 991 |  | 0.947 | *0.618* | *1.451* | *0.802* | 997 |  |
| 4 to 5.9 months | 1.591 | *1.106* | *2.289* | ***0.012*** | 660 |  | 0.972 | *0.608* | *1.555* | *0.906* | 669 |  |
| 6 to 7.9 months | 1.686 | *1.226* | *2.318* | ***0.001*** | 806 |  | 1.044 | *0.658* | *1.656* | *0.855* | 807 |  |
| 8 to 10.9 months | 1.438 | *1.065* | *1.943* | ***0.018*** | 785 |  | 0.815 | *0.548* | *1.212* | *0.313* | 781 |  |
| 10 to 11.9 months | 1.419 | *1.048* | *1.921* | ***0.024*** | 774 |  | 1.552 | *1.082* | *2.228* | ***0.017*** | 749 |  |
| 12 to 13.9 months | 1.177 | *0.831* | *1.668* | *0.359* | 576 |  | 0.983 | *0.657* | *1.472* | *0.935* | 551 |  |
| 14 to 15.9 months | 0.950 | *0.647* | *1.394* | *0.793* | 429 |  | 1.145 | *0.710* | *1.845* | *0.579* | 393 |  |
| 16 to 17.9 months | 1.463 | *0.989* | *2.166* | *0.057* | 452 |  | 0.863 | *0.550* | *1.354* | *0.521* | 393 |  |
| 18 to 19.9 months | 1.259 | *0.836* | *1.898* | *0.270* | 412 |  | 1.218 | *0.734* | *2.021* | *0.446* | 363 |  |
| **Adjusted Odds Ratios** ^#^ | **Stunting in children of primigravidae** | | | | | | **Wasting in children of primigravidae** | | | | | |
| Child age grouping | Adjusted Odds Ratio | *95% CI upper* | *95% CI lower* | *p* | *n* |  | Adjusted Odds Ratio | *95% CI upper* | *95% CI lower* | *p* | *n* |  |
| 0 to 1.9 months | 1.322 | *1.124* | *1.555* | ***0.001*** | 3,135 |  | 1.039 | *0.856* | *1.262* | *0.698* | 2,929 |  |
| 2 to 3.9 months | 1.235 | *0.918* | *1.661* | *0.163* | 972 |  | 0.923 | *0.600* | *1.422* | *0.718* | 978 |  |
| 4 to 5.9 months | 1.675 | *1.146* | *2.448* | ***0.008*** | 651 |  | 0.929 | *0.577* | *1.495* | *0.762* | 660 |  |
| 6 to 7.9 months | 1.740 | *1.256* | *2.410* | ***0.001*** | 801 |  | 1.034 | *0.651* | *1.644* | *0.886* | 801 |  |
| 8 to 10.9 months | 1.480 | *1.083* | *2.023* | ***0.014*** | 778 |  | 0.859 | *0.573* | *1.288* | *0.461* | 774 |  |
| 10 to 11.9 months | 1.505 | *1.099* | *2.062* | ***0.011*** | 768 |  | 1.560 | *1.082* | *2.249* | ***0.017*** | 743 |  |
| 12 to 13.9 months | 1.272 | *0.889* | *1.818* | *0.188* | 568 |  | 1.053 | *0.696* | *1.592* | *0.808* | 544 |  |
| 14 to 15.9 months | 0.989 | *0.659* | *1.484* | *0.957* | 415 |  | 1.143 | *0.693* | *1.886* | *0.601* | 379 |  |
| 16 to 17.9 months | 1.495 | *0.984* | *2.270* | *0.060* | 445 |  | 0.889 | *0.553* | *1.431* | *0.629* | 387 |  |
| 18 to 19.9 months | 1.376 | *0.895* | *2.118* | *0.146* | 412 |  | 1.237 | *0.744* | *2.059* | *0.412* | 356 |  |

† regardless of availability of covariates; ^#^ comparing boys with girls
