# Supplementary material for: Differences in maternal and early child nutritional status by offspring sex in lowland Nepal
Source: Am J Hum Biol. 2021 Jul 6;34(3):e23637. doi: 10.1002/ajhb.23637 (PMC12086752; doi:10.1002/ajhb.23637)
Supplement: Supplementary file 4 — Table S4. Absolute means of length, weight, head circumference, LAZ, WLZ, and HCAZ in girls and boys born to primigravidae, and unadjusted and adjusted coefficients, 95% CIs and p‐values of differences between boys versus girls of primigravidae for these outcomes. [file AJHB-34-e23637-s006.docx]

**Supplemental Table 4. Absolute means of length, weight, head circumference, LAZ, WLZ and HCAZ in girls and boys born to primigravidae, and unadjusted and adjusted coefficients, 95% CIs and p values of differences between boys versus girls of primigravidae for these outcomes**

| **Raw measures for children of primigravidae†** | **Length (cm)** | | | | | | **Weight (kg)** | | | | | | | **Head circumference (cm)** | | | | | | |
| --- | --- | --- | --- | --- | --- | --- | --- | --- | --- | --- | --- | --- | --- | --- | --- | --- | --- | --- | --- | --- |
|  | **Female** | | | **Male** | | | **Female** | | | | **Male** | | | **Female** | | | | **Male** | | |
| Age grouping | Mean | *SD* | *n* | Mean | *SD* | *n* | Mean | *SD* | *n* | Mean | | SD | *n* | Mean | *SD* | n | Mean | | *SD* | *n* |
| 0 to 1.9 months | 49.27 | *3.57* | 1,587 | 49.78 | *3.56* | 1,594 | 3.078 | *0.752* | 1,598 | 3.171 | | 0.799 | 1,613 | 34.01 | *1.98* | 1,590 | 34.47 | | *2.06* | 1,608 |
| 2 to 3.9 months | 56.15 | *3.30* | 495 | 57.51 | *3.57* | 496 | 4.744 | *0.76* | 496 | 5.135 | | 0.84 | 501 | 37.28 | *1.61* | 495 | 38.10 | | *1.67* | 499 |
| 4 to 5.9 months | 61.39 | *3.47* | 323 | 62.78 | *3.46* | 340 | 5.847 | *0.849* | 322 | 6.458 | | 0.914 | 340 | 39.49 | *1.62* | 322 | 40.52 | | *1.65* | 339 |
| 6 to 7.9 months | 64.56 | *2.78* | 380 | 65.76 | *2.68* | 428 | 6.519 | *0.861* | 383 | 7.016 | | 0.851 | 427 | 41.04 | *1.46* | 380 | 41.99 | | *1.48* | 427 |
| 8 to 10.9 months | 66.81 | *2.48* | 381 | 68.37 | *2.74* | 403 | 6.798 | *0.853* | 380 | 7.485 | | 0.889 | 404 | 41.88 | *1.37* | 379 | 43.06 | | *1.30* | 406 |
| 10 to 11.9 months | 68.65 | *2.97* | 352 | 70.43 | *2.85* | 425 | 7.106 | *0.865* | 340 | 7.677 | | 0.956 | 410 | 42.45 | *1.31* | 352 | 43.66 | | *1.38* | 426 |
| 12 to 13.9 months | 70.78 | *2.45* | 274 | 72.68 | *2.87* | 303 | 7.341 | *0.79* | 260 | 8.085 | | 0.963 | 291 | 42.88 | *1.24* | 272 | 44.19 | | *1.42* | 302 |
| 14 to 15.9 months | 72.38 | *2.79* | 209 | 74.35 | *2.69* | 221 | 7.686 | *0.91* | 191 | 8.405 | | 0.93 | 203 | 43.32 | *1.21* | 209 | 44.62 | | *1.18* | 219 |
| 16 to 17.9 months | 74.45 | *2.68* | 227 | 75.88 | *2.78* | 225 | 8.028 | *0.925* | 203 | 8.624 | | 0.923 | 191 | 43.65 | *1.22* | 222 | 44.83 | | *1.26* | 225 |
| 18 to 19.9 months | 75.78 | *3.28* | 200 | 77.42 | *3.11* | 212 | 8.311 | *1.076* | 175 | 9.023 | | 1.112 | 190 | 44.11 | *1.32* | 199 | 45.26 | | *1.47* | 211 |
| **All children 0 to 19.9 months** | **60.20** | ***10.06*** | **4,428** | **61.72** | ***10.48*** | **4,647** | **5.315** | ***2.083*** | **4,348** | **5.792** | | **2.322** | **4,570** | **38.65** | ***4.24*** | **4,420** | **39.67** | | ***4.54*** | **4,662** |
| **Unadjusted Coefficients^#^** | **Length (cm)** | | | | | | **Weight (kg)** | | | | | | | **Head circumference (cm)** | | | | | | |
| Age grouping | Unadjusted Coeff | *95% CI upper* | *95% CI lower* | *p* | *n* |  | Unadjusted Coeff | *95% CI upper* | *95% CI lower* | *p* | | *n* |  | Unadjusted Coeff | *95% CI upper* | *95% CI lower* | *p* | | *n* |  |
| 0 to 1.9 months | 0.49 | *0.24* | *0.73* | **0.000** | 3,225 |  | 0.09 | *0.04* | *0.15* | **0.001** | | 3,256 |  | 0.45 | *0.31* | *0.59* | **<0.001** | | 3,242 |  |
| 2 to 3.9 months | 1.19 | *0.78* | *1.59* | **<0.001** | 997 |  | 0.37 | *0.28* | *0.47* | **<0.001** | | 1,003 |  | 0.71 | *0.52* | *0.90* | **<0.001** | | 1,000 |  |
| 4 to 5.9 months | 1.44 | *0.93* | *1.95* | **<0.001** | 670 |  | 0.61 | *0.47* | *0.74* | **<0.001** | | 669 |  | 1.05 | *0.80* | *1.29* | **<0.001** | | 668 |  |
| 6 to 7.9 months | 1.21 | *0.83* | *1.58* | **<0.001** | 808 |  | 0.50 | *0.38* | *0.62* | **<0.001** | | 810 |  | 0.94 | *0.74* | *1.14* | **<0.001** | | 807 |  |
| 8 to 10.9 months | 1.56 | *1.20* | *1.93* | **<0.001** | 785 |  | 0.69 | *0.56* | *0.81* | **<0.001** | | 785 |  | 1.18 | *1.00* | *1.37* | **<0.001** | | 786 |  |
| 10 to 11.9 months | 1.77 | *1.37* | *2.18* | **<0.001** | 777 |  | 0.57 | *0.44* | *0.70* | **<0.001** | | 750 |  | 1.19 | *1.00* | *1.38* | **<0.001** | | 778 |  |
| 12 to 13.9 months | 1.89 | *1.46* | *2.33* | **<0.001** | 577 |  | 0.73 | *0.58* | *0.88* | **<0.001** | | 551 |  | 1.30 | *1.08* | *1.52* | **<0.001** | | 574 |  |
| 14 to 15.9 months | 1.97 | *1.46* | *2.49* | **<0.001** | 430 |  | 0.71 | *0.53* | *0.89* | **<0.001** | | 394 |  | 1.30 | *1.08* | *1.53* | **<0.001** | | 428 |  |
| 16 to 17.9 months | 1.43 | *0.93* | *1.93* | **<0.001** | 452 |  | 0.60 | *0.41* | *0.78* | **<0.001** | | 394 |  | 1.18 | *0.95* | *1.41* | **<0.001** | | 447 |  |
| 18 to 19.9 months | 1.62 | *1.00* | *2.23* | **<0.001** | 412 |  | 0.68 | *0.45* | *0.90* | **<0.001** | | 365 |  | 1.14 | *0.87* | *1.41* | **<0.001** | | 410 |  |
| **Adjusted Coefficients^#^** | **Length (cm)** | | | | | | **Weight (kg)** | | | | | | | **Head circumference (cm)** | | | | | | |
| Age grouping | Adjusted Coeff | *95% CI upper* | *95% CI lower* | *p* | *n* |  | Adjusted Coeff | *95% CI upper* | *95% CI lower* | *p* | | *n* |  | Adjusted Coeff | *95% CI upper* | *95% CI lower* | *p* | | *n* |  |
| 0 to 1.9 months | 0.47 | *0.29* | *0.65* | **<0.001** | 3,151 |  | 0.09 | *0.05* | *0.13* | **<0.001** | | 3,181 |  | 0.45 | *0.34* | *0.56* | **<0.001** | | 3,168 |  |
| 2 to 3.9 months | 1.29 | *0.92* | *1.67* | **<0.001** | 978 |  | 0.41 | *0.32* | *0.50* | **<0.001** | | 984 |  | 0.76 | *0.58* | *0.94* | **<0.001** | | 981 |  |
| 4 to 5.9 months | 1.37 | *0.90* | *1.84* | **<0.001** | 661 |  | 0.60 | *0.47* | *0.72* | **<0.001** | | 660 |  | 1.01 | *0.79* | *1.24* | **<0.001** | | 659 |  |
| 6 to 7.9 months | 1.29 | *0.93* | *1.65* | **<0.001** | 802 |  | 0.51 | *0.40* | *0.63* | **<0.001** | | 803 |  | 0.97 | *0.78* | *1.16* | **<0.001** | | 801 |  |
| 8 to 10.9 months | 1.50 | *1.14* | *1.86* | **<0.001** | 778 |  | 0.66 | *0.53* | *0.78* | **<0.001** | | 777 |  | 1.14 | *0.95* | *1.32* | **<0.001** | | 778 |  |
| 10 to 11.9 months | 1.71 | *1.31* | *2.10* | **<0.001** | 771 |  | 0.55 | *0.42* | *0.68* | **<0.001** | | 744 |  | 1.17 | *0.98* | *1.36* | **<0.001** | | 772 |  |
| 12 to 13.9 months | 1.81 | *1.38* | *2.23* | **<0.001** | 569 |  | 0.70 | *0.55* | *0.84* | **<0.001** | | 544 |  | 1.24 | *1.03* | *1.46* | **<0.001** | | 567 |  |
| 14 to 15.9 months | 1.95 | *1.46* | *2.44* | **<0.001** | 416 |  | 0.71 | *0.54* | *0.89* | **<0.001** | | 380 |  | 1.30 | *1.08* | *1.51* | **<0.001** | | 414 |  |
| 16 to 17.9 months | 1.51 | *1.03* | *2.00* | **<0.001** | 445 |  | 0.59 | *0.41* | *0.77* | **<0.001** | | 388 |  | 1.18 | *0.95* | *1.41* | **<0.001** | | 440 |  |
| 18 to 19.9 months | 1.50 | *0.91* | *2.09* | **<0.001** | 404 |  | 0.64 | *0.43* | *0.85* | **<0.001** | | 358 |  | 1.12 | *0.86* | *1.38* | **<0.001** | | 402 |  |
| **Raw measures for children of primigravidae†** | **Length-for-age *z* score (LAZ)** | | | | | | **Weight-for-length z score (WLZ)** | | | | | | | **Head Circumference-for-age z score (HCAZ)** | | | | | | |
|  | **Female** | | | **Male** | | | **Female** | | | | **Male** | | | **Female** | | | | **Male** | | |
| Age grouping | Mean | *SD* | *n* | Mean | *SD* | *n* | Mean | *SD* | *n* | Mean | | *SD* | *n* | Mean | *SD* | *n* | Mean | | *SD* | *n* |
| 0 to 1.9 months | -1.29 | *1.23* | 1,578 | -1.48 | *1.27* | 1,587 | -0.86 | *1.25* | 1,455 | -0.78 | | *1.31* | 1,482 | -1.14 | *1.22* | 1,574 | -1.23 | | *1.24* | 1,582 |
| 2 to 3.9 months | -1.44 | *1.41* | 492 | -1.51 | *1.55* | 493 | -0.35 | *1.52* | 491 | -0.35 | | *1.37* | 491 | -1.55 | *1.12* | 488 | -1.66 | | *1.23* | 489 |
| 4 to 5.9 months | -1.18 | *1.23* | 318 | -1.42 | *1.39* | 335 | -0.71 | *1.12* | 319 | -0.46 | | *1.35* | 338 | -1.55 | *1.15* | 322 | -1.62 | | *1.16* | 331 |
| 6 to 7.9 months | -1.18 | *1.17* | 380 | -1.54 | *1.15* | 426 | -0.79 | *0.99* | 380 | -0.77 | | *0.99* | 427 | -1.35 | *0.98* | 377 | -1.58 | | *1.10* | 425 |
| 8 to 10.9 months | -1.39 | *1.02* | 381 | -1.61 | *1.21* | 403 | -1.11 | *0.96* | 378 | -0.96 | | *1.01* | 400 | -1.46 | *1.02* | 379 | -1.52 | | *0.98* | 404 |
| 10 to 11.9 months | -1.60 | *1.02* | 351 | -1.73 | *1.12* | 423 | -1.22 | *0.89* | 338 | -1.33 | | *1.03* | 410 | -1.55 | *0.90* | 351 | -1.61 | | *1.00* | 424 |
| 12 to 13.9 months | -1.66 | *0.92* | 274 | -1.71 | *1.07* | 302 | -1.43 | *0.86* | 259 | -1.35 | | *0.96* | 289 | -1.65 | *0.88* | 271 | -1.63 | | *1.02* | 301 |
| 14 to 15.9 months | -1.85 | *1.01* | 209 | -1.84 | *0.97* | 220 | -1.37 | *0.92* | 191 | -1.36 | | *1.02* | 202 | -1.69 | *0.88* | 209 | -1.66 | | *0.89* | 219 |
| 16 to 17.9 months | -1.83 | *0.94* | 227 | -2.01 | *1.05* | 225 | -1.39 | *0.96* | 203 | -1.47 | | *0.89* | 190 | -1.75 | *0.89* | 222 | -1.75 | | *0.90* | 223 |
| 18 to 19.9 months | -1.99 | *1.12* | 200 | -2.09 | *1.13* | 212 | -1.32 | *0.95* | 174 | -1.30 | | *1.05* | 189 | -1.66 | *0.96* | 199 | -1.68 | | *1.08* | 210 |
| **All children 0 to 19.9 months** | **-1.43** | ***1.19*** | **4,410** | **-1.60** | ***1.26*** | **4,626** | **-0.94** | ***1.19*** | **4,188** | **-0.89** | | ***1.23*** | **4,418** | **-1.40** | ***1.10*** | **4,392** | **-1.49** | | ***1.14*** | **4,608** |
| **Unadjusted Coefficients^#^** | **Length-for-age z score (LAZ)** | | | | | | **Weight-for-length z score (WLZ)** | | | | | | | **Head Circumference-for-age z score (HCAZ)** | | | | | | |
| Age grouping | Unadjusted Coeff | *95% CI upper* | *95% CI lower* | *p* | *n* |  | Unadjusted Coeff | *95% CI upper* | *95% CI lower* | *p* | | *n* |  | Unadjusted Coeff | *95% CI upper* | *95% CI lower* | *p* | | *n* |  |
| 0 to 1.9 months | -0.206 | *-0.291* | *-0.121* | **<0.001** | 3,209 |  | 0.084 | *-0.006* | *0.174* | 0.067 | | 2,981 |  | -0.099 | *-0.182* | *-0.016* | **0.020** | | 3,200 |  |
| 2 to 3.9 months | -0.144 | *-0.322* | *0.033* | 0.111 | 991 |  | 0.054 | *-0.118* | *0.226* | 0.540 | | 988 |  | -0.203 | *-0.342* | *-0.064* | **0.004** | | 981 |  |
| 4 to 5.9 months | -0.229 | *-0.428* | *-0.031* | **0.024** | 660 |  | 0.224 | *0.037* | *0.412* | **0.019** | | 664 |  | -0.075 | *-0.251* | *0.102* | 0.408 | | 660 |  |
| 6 to 7.9 months | -0.352 | *-0.512* | *-0.192* | **<0.001** | 806 |  | 0.022 | *-0.115* | *0.158* | 0.757 | | 807 |  | -0.243 | *-0.387* | *-0.098* | **0.001** | | 802 |  |
| 8 to 10.9 months | -0.218 | *-0.375* | *-0.061* | **0.007** | 785 |  | 0.150 | *0.013* | *0.288* | **0.033** | | 779 |  | -0.062 | *-0.202* | *0.077* | 0.379 | | 784 |  |
| 10 to 11.9 months | -0.139 | *-0.291* | *0.012* | 0.072 | 774 |  | -0.111 | *-0.249* | *0.027* | 0.115 | | 748 |  | -0.078 | *-0.212* | *0.055* | 0.250 | | 775 |  |
| 12 to 13.9 months | -0.069 | *-0.230* | *0.093* | 0.405 | 576 |  | 0.069 | *-0.083* | *0.221* | 0.374 | | 548 |  | 0.007 | *-0.147* | *0.162* | 0.926 | | 572 |  |
| 14 to 15.9 months | 0.013 | *-0.173* | *0.200* | 0.887 | 429 |  | 0.015 | *-0.177* | *0.206* | 0.880 | | 393 |  | 0.034 | *-0.134* | *0.201* | 0.693 | | 428 |  |
| 16 to 17.9 months | -0.179 | *-0.362* | *0.003* | 0.055 | 452 |  | -0.080 | *-0.262* | *0.103* | 0.393 | | 393 |  | -0.003 | *-0.168* | *0.163* | 0.975 | | 445 |  |
| 18 to 19.9 months | -0.111 | *-0.328* | *0.105* | 0.315 | 412 |  | -0.028 | *-0.231* | *0.174* | 0.784 | | 363 |  | -0.030 | *-0.227* | *0.167* | 0.762 | | 409 |  |
| **Adjusted Coefficients^#^** | **Length-for-age z score (LAZ)** | | | | | | **Weight-for-length z score (WLZ)** | | | | | | | **Head Circumference-for-age z score (HCAZ)** | | | | | | |
| Age grouping | Adjusted Coeff | *95% CI upper* | *95% CI lower* | *p* | *n* |  | Adjusted Coeff | *95% CI upper* | *95% CI lower* | *p* | | *n* |  | Adjusted Coeff | *95% CI upper* | *95% CI lower* | *p* | | *n* |  |
| 0 to 1.9 months | -0.209 | *-0.295* | *-0.124* | **<0.001** | 3,135 |  | 0.086 | *-0.005* | *0.177* | 0.064 | | 2,908 |  | -0.098 | *-0.181* | *-0.016* | **0.020** | | 3,126 |  |
| 2 to 3.9 months | -0.151 | *-0.330* | *0.028* | 0.098 | 972 |  | 0.048 | *-0.126* | *0.222* | 0.589 | | 969 |  | -0.204 | *-0.343* | *-0.064* | **0.004** | | 964 |  |
| 4 to 5.9 months | -0.239 | *-0.435* | *-0.043* | **0.017** | 651 |  | 0.248 | *0.060* | *0.436* | **0.010** | | 655 |  | -0.084 | *-0.257* | *0.089* | 0.341 | | 651 |  |
| 6 to 7.9 months | -0.339 | *-0.498* | *-0.180* | **<0.001** | 801 |  | 0.016 | *-0.121* | *0.152* | 0.821 | | 801 |  | -0.233 | *-0.378* | *-0.089* | **0.002** | | 797 |  |
| 8 to 10.9 months | -0.251 | *-0.407* | *-0.094* | **0.002** | 778 |  | 0.114 | *-0.024* | *0.253* | 0.105 | | 772 |  | -0.099 | *-0.240* | *0.042* | 0.167 | | 776 |  |
| 10 to 11.9 months | -0.160 | *-0.309* | *-0.011* | **0.035** | 768 |  | -0.111 | *-0.248* | *0.027* | 0.114 | | 742 |  | -0.100 | *-0.233* | *0.033* | 0.142 | | 769 |  |
| 12 to 13.9 months | -0.083 | *-0.244* | *0.078* | 0.311 | 568 |  | 0.047 | *-0.106* | *0.200* | 0.550 | | 541 |  | -0.026 | *-0.178* | *0.126* | 0.739 | | 565 |  |
| 14 to 15.9 months | -0.005 | *-0.185* | *0.175* | 0.959 | 415 |  | 0.015 | *-0.178* | *0.208* | 0.878 | | 379 |  | 0.027 | *-0.135* | *0.190* | 0.744 | | 414 |  |
| 16 to 17.9 months | -0.170 | *-0.348* | *0.008* | 0.061 | 445 |  | -0.103 | *-0.286* | *0.081* | 0.272 | | 387 |  | -0.012 | *-0.177* | *0.152* | 0.882 | | 438 |  |
| 18 to 19.9 months | -0.156 | *-0.363* | *0.050* | 0.137 | 404 |  | -0.038 | *-0.238* | *0.162* | 0.709 | | 356 |  | -0.046 | *-0.236* | *0.143* | 0.633 | | 401 |  |

† for all available cases of children of primigravidae regardless of availability of covariates; ^#^ comparing boys of primigravidae with girls of primigravidae mothers
