# Supplementary material for: Differences in maternal and early child nutritional status by offspring sex in lowland Nepal
Source: Am J Hum Biol. 2021 Jul 6;34(3):e23637. doi: 10.1002/ajhb.23637 (PMC12086752; doi:10.1002/ajhb.23637)
Supplement: Supplementary file 5 — Table S5. Absolute means of LAZ, WLZ, and HCAZ in the first 7.9 days of life in girls and boys, and unadjusted and adjusted coefficients, 95% CIs and p values of differences between boys versus girls for these outcomes in children of all mothers, not underweight, underweight, not short and short mothers. [file AJHB-34-e23637-s004.docx]

**Supplemental Table 5. Absolute means of LAZ, WLZ and HCAZ in the first 7.9 days of life in girls and boys, and unadjusted and adjusted coefficients, 95% CIs and p values of differences between boys versus girls for these outcomes in children of all mothers, not underweight, underweight, not short and short mothers.**

| **Raw measures for all available cases†** | **Length-for-age *z* score (LAZ)** | | | | | | **Weight-for-length z score (WLZ)** | | | | | | **Head Circumference-for-age z score (HCAZ)** | | | | | |
| --- | --- | --- | --- | --- | --- | --- | --- | --- | --- | --- | --- | --- | --- | --- | --- | --- | --- | --- |
|  | **Female** | | | **Male** | | | **Female** | | | **Male** | | | **Female** | | | **Male** | | |
| Child age 0 to 7.9 days | Mean | *SD* | *n* | Mean | *SD* | *n* | Mean | *SD* | *n* | Mean | *SD* | *n* | Mean | *SD* | *n* | Mean | *SD* | *n* |
| All mothers | -1.02 | 1.10 | 2,023 | -1.14 | 1.14 | 2,212 | -0.77 | 1.21 | 1,838 | -0.65 | 1.22 | 2,045 | -0.81 | 1.14 | 2,016 | -0.80 | 1.09 | 2,211 |
| Not underweight mothers >=18.5 kg/m^2^ | -0.93 | 1.09 | 1,007 | -1.08 | 1.16 | 1,096 | -0.73 | 1.24 | 928 | -0.60 | 1.21 | 1,011 | -0.75 | 1.15 | 1,006 | -0.74 | 1.06 | 1,091 |
| Underweight mothers <18.5 kg/m^2^ | -1.14 | 1.11 | 395 | -1.23 | 1.13 | 491 | -0.87 | 1.18 | 355 | -0.84 | 1.19 | 451 | -0.85 | 1.07 | 395 | -0.97 | 1.13 | 491 |
| Not Short mothers >=145cm | -0.95 | 1.08 | 1,314 | -1.05 | 1.13 | 1,499 | -0.73 | 1.21 | 1,217 | -0.66 | 1.20 | 1,400 | -0.74 | 1.13 | 1,313 | -0.75 | 1.09 | 1,496 |
| Short mothers <145cm | -1.27 | 1.13 | 261 | -1.62 | 1.13 | 283 | -0.93 | 1.26 | 221 | -0.65 | 1.23 | 242 | -1.00 | 1.20 | 258 | -1.03 | 1.08 | 283 |
| **Unadjusted Coefficients ^#^** | **Length-for-age *z* score (LAZ)** | | | | | | **Weight-for-length z score (WLZ)** | | | | | | **Head Circumference-for-age z score (HCAZ)** | | | | | |
| Child age 0 to 7.9 days | Unadjusted Coeff | *95% CI upper* | *95% CI lower* | *p* | *n* |  | Unadjusted Coeff | *95% CI upper* | *95% CI lower* | *p* | *n* |  | Unadjusted Coeff | *95% CI upper* | *95% CI lower* | *p* | *n* |  |
| All mothers | -0.112 | *-0.178* | *-0.047* | **0.001** | 4,251 |  | 0.121 | *0.047* | *0.196* | **0.001** | 3,898 |  | 0.014 | *-0.050* | *0.078* | 0.678 | 4,243 |  |
| Not underweight mothers >=18.5 kg/m^2^ | -0.138 | *-0.232* | *-0.045* | **0.004** | 2,113 |  | 0.112 | *0.005* | *0.218* | **0.040** | 1,948 |  | 0.000 | *-0.090* | *0.090* | 0.996 | 2,107 |  |
| Underweight mothers <18.5 kg/m^2^ | -0.103 | *-0.251* | *0.045* | 0.172 | 888 |  | 0.041 | *-0.123* | *0.205* | 0.622 | 808 |  | -0.116 | *-0.261* | *0.029* | 0.116 | 888 |  |
| Not Short mothers >=145cm | -0.098 | *-0.178* | *-0.019* | **0.015** | 2,822 |  | 0.080 | *-0.010* | *0.170* | 0.082 | 2,626 |  | -0.003 | *-0.082* | *0.075* | 0.933 | 2,818 |  |
| Short mothers <145cm | -0.331 | *-0.520* | *-0.141* | **0.001** | 547 |  | 0.252 | *0.031* | *0.473* | **0.025** | 465 |  | -0.016 | *-0.198* | *0.166* | 0.864 | 544 |  |
| **Adjusted Coefficients** ^#^ | **Length-for-age *z* score (LAZ)** | | | | | | **Weight-for-length z score (WLZ)** | | | | | | **Head Circumference-for-age z score (HCAZ)** | | | | | |
| Child age 0 to 7.9 days | Adjusted Coeff | *95% CI upper* | *95% CI lower* | *p* | *n* |  | Adjusted Coeff | *95% CI upper* | *95% CI lower* | *p* | *n* |  | Adjusted Coeff | *95% CI upper* | *95% CI lower* | *p* | *n* |  |
| All mothers | -0.126 | *-0.190* | *-0.061* | **0.000** | 4,222 |  | 0.113 | *0.039* | *0.187* | **0.003** | 3,869 |  | 0.005 | *-0.059* | *0.069* | 0.881 | 4,215 |  |
| Not underweight mothers >=18.5 kg/m^2^ | -0.152 | *-0.245* | *-0.058* | **0.001** | 2,104 |  | 0.107 | *0.001* | *0.213* | **0.048** | 1,939 |  | -0.007 | *-0.097* | *0.082* | 0.872 | 2,098 |  |
| Underweight mothers <18.5 kg/m^2^ | -0.111 | *-0.259* | *0.036* | 0.139 | 887 |  | 0.036 | *-0.128* | *0.201* | 0.665 | 807 |  | -0.119 | *-0.263* | *0.025* | 0.106 | 887 |  |
| Not Short mothers >=145cm | -0.113 | *-0.191* | *-0.034* | **0.005** | 2,811 |  | 0.075 | *-0.015* | *0.165* | 0.103 | 2,615 |  | -0.011 | *-0.089* | *0.067* | 0.778 | 2,807 |  |
| Short mothers <145cm | -0.322 | *-0.510* | *-0.135* | **0.001** | 545 |  | 0.253 | *0.036* | *0.471* | **0.023** | 463 |  | -0.018 | *-0.199* | *0.163* | 0.849 | 542 |  |
| **Analyses**^#^ **with interaction terms between maternal BMI and child sex (all mothers)** | **Length-for-age *z* score (LAZ) interaction models** | | | | | | **Weight-for-length z score (WLZ) interaction models** | | | | | | **Head Circumference-for-age z score (HCAZ) interaction models** | | | | | |
| Child age 0 to 7.9 days | Interaction term coeff. | *95% CI upper* | *95% CI lower* | *p* | *n* |  | Interaction term coeff. | *95% CI upper* | *95% CI lower* | *p* | *n* |  | Interaction term coeff. | *95% CI upper* | *95% CI lower* | *p* | *n* |  |
| **Unadjusted Interaction model with male X maternal underweight (BMI <18.5** kg/m^2^**)** |  |  |  |  | 3,001 |  |  |  |  |  | 2,756 |  |  |  |  |  | 2,995 |  |
| Unadjusted coefficient for child sex | -0.136 | *-0.229* | *-0.042* | **0.005** |  |  | 0.111 | *0.006* | *0.216* | **0.039** |  |  | 0.006 | *-0.084* | *0.096* | 0.894 |  |  |
| Unadjusted coefficient for maternal underweight (BMI <18.5 kg/m^2^) | -0.202 | *-0.330* | *-0.074* | **0.002** |  |  | -0.160 | *-0.305* | *-0.015* | **0.030** |  |  | -0.162 | *-0.285* | *-0.038* | **0.010** |  |  |
| Unadjusted coefficient for interaction of male X underweight | 0.035 | *-0.138* | *0.209* | 0.688 |  |  | -0.059 | *-0.254* | *0.137* | 0.556 |  |  | -0.101 | *-0.268* | *0.066* | 0.236 |  |  |
| **Adjusted Interaction model with male X maternal underweight (BMI <18.5** **kg/m^2^)** |  |  |  |  | 3,369 |  |  |  |  |  | 3,091 |  |  |  |  |  | 3,362 |  |
| Adjusted coefficient for child sex (male) | -0.149 | *-0.242* | *-0.056* | **0.002** |  |  | 0.104 | *-0.001* | *0.208* | 0.052 |  |  | -0.002 | *-0.092* | *0.088* | 0.963 |  |  |
| Adjusted coefficient for maternal underweight (BMI <18.5 kg/m^2^) | -0.182 | *-0.309* | *-0.054* | **0.005** |  |  | -0.155 | *-0.299* | *-0.011* | **0.035** |  |  | -0.148 | *-0.271* | *-0.025* | **0.018** |  |  |
| Adjusted coefficient for interaction of male X underweight | 0.038 | *-0.134* | *0.210* | 0.668 |  |  | -0.054 | *-0.249* | *0.141* | 0.587 |  |  | -0.094 | *-0.260* | *0.072* | 0.265 |  |  |
| **Analyses**^#^ **with interaction terms between maternal stature and child sex (all mothers)** | **Length-for-age *z* score (LAZ) interaction models** | | | | | | **Weight-for-length z score (WLZ) interaction models** | | | | | | **Head Circumference-for-age z score (HCAZ) interaction models** | | | | | |
| **Unadjusted Interaction model with male X maternal short stature (<145cm)** |  |  |  |  | 2,991 |  |  |  |  |  | 2,746 |  |  |  |  |  | 2,985 |  |
| Unadjusted coefficient for child sex | -0.097 | *-0.177* | *-0.018* | **0.017** |  |  | 0.082 | *-0.008* | *0.173* | 0.074 |  |  | -0.003 | *-0.082* | *0.075* | 0.936 |  |  |
| Unadjusted coefficient for maternal short stature (<145cm) | -0.331 | *-0.474* | *-0.189* | **<0.001** |  |  | -0.177 | *-0.346* | *-0.008* | **0.040** |  |  | -0.252 | *-0.393* | *-0.111* | **<0.001** |  |  |
| Unadjusted coefficient for interaction of male X maternal short stature (<145cm) | -0.209 | *-0.406* | *-0.012* | **0.038** |  |  | 0.173 | *-0.059* | *0.405* | 0.143 |  |  | -0.021 | *-0.216* | *0.173* | 0.830 |  |  |
| **Adjusted Interaction model with male X maternal short stature (<145cm)** |  |  |  |  | 3,356 |  |  |  |  |  | 3,078 |  |  |  |  |  | 3,349 |  |
| Adjusted coefficient for child sex (male) | -0.111 | *-0.190* | *-0.032* | **0.006** |  |  | 0.076 | *-0.013* | *0.166* | 0.095 |  |  | -0.010 | *-0.088* | *0.068* | 0.804 |  |  |
| Adjusted coefficient for maternal short stature | -0.337 | *-0.479* | *-0.195* | **<0.001** |  |  | -0.169 | *-0.338* | *0.000* | **0.050** |  |  | -0.252 | *-0.394* | *-0.111* | **<0.001** |  |  |
| Adjusted coefficient for interaction of male X short stature | -0.196 | *-0.391* | *0.000* | **0.050** |  |  | 0.175 | *-0.056* | *0.407* | 0.137 |  |  | -0.012 | *-0.205* | *0.182* | 0.907 |  |  |

^†^ regardless of availability of covariates; ^#^ comparing boys with girls
