# Supplementary material for: Differences in maternal and early child nutritional status by offspring sex in lowland Nepal
Source: Am J Hum Biol. 2021 Jul 6;34(3):e23637. doi: 10.1002/ajhb.23637 (PMC12086752; doi:10.1002/ajhb.23637)
Supplement: Supplementary file 6 — Table S6. Absolute means of MUAC, BMI, and weight in mothers of girls and boys in pregnancy and postpartum, and unadjusted and adjusted coefficients, 95% CIs, and p‐values of differences between mothers of boys versus girls for these outcomes. [file AJHB-34-e23637-s013.docx]

**Supplemental Table 6. Absolute means of MUAC, BMI, and weight in mothers of girls and boys in pregnancy and postpartum, and unadjusted and adjusted coefficients, 95% CIs and p values of differences between mothers of boys versus girls for these outcomes**

| **Raw measures†** | **Pregnancy MUAC (cm)** | | | | | | **Pregnancy BMI (kg/m^2)** | | | | | |
| --- | --- | --- | --- | --- | --- | --- | --- | --- | --- | --- | --- | --- |
|  | **Female** | | | **Male** | | | **Female** | | | **Male** | | |
| Gestational age grouping | Mean | *SD* | n | Mean | *SD* | n | Mean | *SD* | n | Mean | *SD* | n |
| 12 to 15.9 weeks | 23.1 | 2.1 | 207 | 23.2 | 2.1 | 232 | 19.8 | 2.6 | 207 | 19.9 | 2.2 | 232 |
| 16 to 19.9 weeks | 23.4 | 2.1 | 312 | 23.3 | 2.1 | 304 | 20.3 | 2.3 | 312 | 20.4 | 2.2 | 304 |
| 20 to 23.9 weeks | 23.3 | 1.8 | 336 | 23.3 | 2.0 | 405 | 20.9 | 2.1 | 336 | 20.8 | 2.4 | 405 |
| 24 to 27.9 weeks | 23.6 | 2.2 | 422 | 23.6 | 2.1 | 476 | 21.5 | 2.6 | 422 | 21.6 | 2.4 | 476 |
| 28 to 31.9 weeks | 23.7 | 2.0 | 323 | 23.7 | 2.1 | 368 | 22.0 | 2.5 | 321 | 22.1 | 2.2 | 367 |
| 32 to 35.9 weeks | 23.6 | 2.1 | 589 | 23.6 | 2.1 | 675 | 22.5 | 2.5 | 581 | 22.5 | 2.3 | 666 |
| 36 to 39.9 weeks | 23.5 | 2.1 | 466 | 23.8 | 2.1 | 435 | 22.5 | 2.5 | 461 | 23.0 | 2.6 | 429 |
| **All cases 12 to 29.9 wks** | **23.5** | **2.1** | **2655** | **23.5** | **2.1** | **2,895** | **21.6** | **2.6** | **2640** | **21.7** | **2.6** | **2,879** |
| **Unadjusted Coefficients^#^** | **Pregnancy MUAC (cm)** | | | | | | **Pregnancy BMI (kg/m^2)** | | | | | |
| Gestational age grouping | Unadjusted Coeff | *Upper 95% CI* | *Lower 95% CI* | *p* | *n* |  | Unadjusted Coeff | *Upper 95% CI* | *Lower 95% CI* | *p* | *n* |  |
| 12 to 15.9 weeks | 0.18 | *-0.22* | *0.57* | 0.375 | 439 |  | 0.16 | *-0.29* | *0.61* | 0.484 | 439 |  |
| 16 to 19.9 weeks | -0.07 | *-0.39* | *0.25* | 0.680 | 616 |  | 0.04 | *-0.32* | *0.39* | 0.840 | 616 |  |
| 20 to 23.9 weeks | -0.01 | *-0.29* | *0.26* | 0.930 | 741 |  | -0.12 | *-0.45* | *0.21* | 0.466 | 741 |  |
| 24 to 27.9 weeks | -0.04 | *-0.32* | *0.24* | 0.801 | 898 |  | 0.13 | *-0.19* | *0.46* | 0.419 | 898 |  |
| 28 to 31.9 weeks | -0.03 | *-0.34* | *0.27* | 0.843 | 691 |  | 0.08 | *-0.27* | *0.43* | 0.659 | 688 |  |
| 32 to 35.9 weeks | -0.02 | *-0.25* | *0.21* | 0.887 | 1264 |  | -0.02 | *-0.29* | *0.24* | 0.874 | 1247 |  |
| 36 to 39.9 weeks | 0.30 | *0.03* | *0.57* | **0.030** | 902 |  | 0.50 | *0.17* | *0.84* | **0.004** | 890 |  |
| **Adjusted Coefficients^#^** | **Pregnancy MUAC (cm)** | | | | | | **Pregnancy BMI (kg/m^2)** | | | | | |
| Gestational age grouping | Adjusted Coeff | *Upper 95% CI* | *Lower 95% CI* | *p* | *n* |  | Adjusted Coeff | *Upper 95% CI* | *Lower 95% CI* | *p* | *n* |  |
| 12 to 15.9 weeks | 0.22 | *-0.17* | *0.60* | 0.277 | 437 |  | 0.18 | *-0.26* | *0.62* | 0.427 | 437 |  |
| 16 to 19.9 weeks | -0.10 | *-0.42* | *0.22* | 0.542 | 616 |  | 0.02 | *-0.33* | *0.38* | 0.899 | 616 |  |
| 20 to 23.9 weeks | 0.00 | *-0.28* | *0.28* | 0.993 | 741 |  | -0.12 | *-0.45* | *0.21* | 0.466 | 741 |  |
| 24 to 27.9 weeks | -0.06 | *-0.34* | *0.22* | 0.661 | 896 |  | 0.10 | *-0.22* | *0.43* | 0.535 | 896 |  |
| 28 to 31.9 weeks | 0.00 | *-0.29* | *0.30* | 0.983 | 691 |  | 0.10 | *-0.24* | *0.44* | 0.563 | 688 |  |
| 32 to 35.9 weeks | -0.04 | *-0.26* | *0.20* | 0.767 | 1258 |  | 0.00 | *-0.27* | *0.26* | 0.980 | 1242 |  |
| 36 to 39.9 weeks | 0.27 | *0.00* | *0.54* | 0.050 | 896 |  | 0.46 | *0.12* | *0.80* | **0.008** | 884 |  |
| **Raw measures†** | **Postpartum MUAC cm** | | | | | | **Postpartum BMI (kg/m^2)** | | | | | |
|  | **Female** | | | **Male** | | | **Female** | | | **Male** | | |
| Time since delivery | Mean | *SD* | n | Mean | *SD* | n | Mean | *SD* | n | Mean | *SD* | n |
| 0 to 1.9 months | 23.70 | 2.22 | 942 | 23.60 | 2.16 | 1013 | 20.74 | 2.45 | 898 | 20.70 | 2.36 | 966 |
| 2 to 3.9 months | 23.70 | 2.26 | 1144 | 23.75 | 2.28 | 1283 | 20.54 | 2.56 | 1082 | 20.48 | 2.53 | 1203 |
| 4 to 5.9 months | 23.78 | 2.20 | 701 | 23.83 | 2.29 | 799 | 20.10 | 2.47 | 674 | 20.14 | 2.54 | 771 |
| 6 to 7.9 months | 23.87 | 2.42 | 755 | 23.83 | 2.43 | 911 | 19.92 | 2.53 | 745 | 20.00 | 2.80 | 899 |
| 8 to 10.9 months | 23.76 | 2.35 | 950 | 23.68 | 2.41 | 1001 | 19.88 | 2.66 | 947 | 19.72 | 2.56 | 993 |
| 10 to 11.9 months | 23.58 | 2.39 | 908 | 23.40 | 2.26 | 1068 | 19.57 | 2.59 | 904 | 19.43 | 2.43 | 1063 |
| 12 to 13.9 months | 23.55 | 2.41 | 725 | 23.42 | 2.28 | 866 | 19.53 | 2.56 | 722 | 19.43 | 2.42 | 860 |
| 14 to 15.9 months | 23.44 | 2.40 | 560 | 23.43 | 2.21 | 682 | 19.48 | 2.66 | 556 | 19.43 | 2.45 | 680 |
| 16 to 17.9 months | 23.30 | 2.28 | 479 | 23.46 | 2.18 | 550 | 19.46 | 2.37 | 476 | 19.50 | 2.50 | 546 |
| 18 to 19.9 months | 23.37 | 2.22 | 168 | 23.54 | 2.39 | 205 | 19.57 | 2.58 | 168 | 19.52 | 2.54 | 203 |
| **All cases 0 to 19.9 months postpartum** | 23.65 | 2.32 | 7,332 | 23.61 | 2.29 | 8,378 | 19.97 | 2.59 | 7,172 | 19.91 | 2.56 | 8,184 |
| **Unadjusted Coefficients^#^** | **Postpartum MUAC** | | | | | | **Postpartum BMI** | | | | | |
| Grouping of time since delivery | Unadjusted Coeff | *Upper 95% CI* | *Lower 95% CI* | *p* | *n* |  | Unadjusted Coeff | *Upper 95% CI* | *Lower 95% CI* | *p* | *n* |  |
| 0 to 1.9 months | -0.12 | *-0.31* | *0.07* | 0.226 | 1955 |  | -0.04 | *-0.26* | *0.18* | 0.734 | 1864 |  |
| 2 to 3.9 months | 0.07 | *-0.11* | *0.25* | 0.433 | 2,427 |  | -0.05 | *-0.25* | *0.16* | 0.658 | 2,285 |  |
| 4 to 5.9 months | 0.05 | *-0.18* | *0.28* | 0.665 | 1,500 |  | 0.03 | *-0.23* | *0.29* | 0.809 | 1,445 |  |
| 6 to 7.9 months | -0.05 | *-0.29* | *0.18* | 0.661 | 1,666 |  | 0.07 | *-0.19* | *0.33* | 0.601 | 1,644 |  |
| 8 to 10.9 months | -0.09 | *-0.30* | *0.12* | 0.396 | 1,951 |  | -0.16 | *-0.39* | *0.07* | 0.180 | 1,940 |  |
| 10 to 11.9 months | -0.18 | *-0.39* | *0.02* | 0.081 | 1,976 |  | -0.13 | *-0.35* | *0.09* | 0.245 | 1,967 |  |
| 12 to 13.9 months | -0.16 | *-0.39* | *0.07* | 0.179 | 1,591 |  | -0.13 | *-0.37* | *0.12* | 0.302 | 1,582 |  |
| 14 to 15.9 months | -0.01 | *-0.27* | *0.24* | 0.919 | 1,242 |  | -0.05 | *-0.33* | *0.23* | 0.726 | 1,236 |  |
| 16 to 17.9 months | 0.15 | *-0.12* | *0.42* | 0.282 | 1,029 |  | 0.04 | *-0.26* | *0.34* | 0.797 | 1,022 |  |
| 18 to 19.9 months | 0.17 | *-0.31* | *0.64* | 0.490 | 373 |  | -0.05 | *-0.57* | *0.47* | 0.859 | 371 |  |
| **Adjusted Coefficients^#^** | **Postpartum MUAC** | | | | | | **Postpartum BMI** | | | | | |
| Grouping of time since delivery | Adjusted Coeff | *Upper 95% CI* | *Lower 95% CI* | *p* | *n* |  | Adjusted Coeff | *Upper 95% CI* | *Lower 95% CI* | *p* | *n* |  |
| 0 to 1.9 months | -0.14 | *-0.33* | *0.05* | 0.161 | 1928 |  | -0.04 | *-0.26* | *0.18* | 0.743 | 1839 |  |
| 2 to 3.9 months | 0.05 | *-0.13* | *0.22* | 0.584 | 2,407 |  | -0.07 | *-0.28* | *0.14* | 0.498 | 2,268 |  |
| 4 to 5.9 months | 0.03 | *-0.20* | *0.25* | 0.812 | 1,487 |  | -0.01 | *-0.27* | *0.24* | 0.915 | 1,434 |  |
| 6 to 7.9 months | -0.11 | *-0.34* | *0.13* | 0.368 | 1,658 |  | 0.01 | *-0.25* | *0.27* | 0.941 | 1,636 |  |
| 8 to 10.9 months | -0.13 | *-0.34* | *0.08* | 0.215 | 1,942 |  | -0.21 | *-0.44* | *0.02* | 0.073 | 1,932 |  |
| 10 to 11.9 months | -0.22 | *-0.42* | *-0.01* | 0.038 | 1,971 |  | -0.17 | *-0.39* | *0.06* | 0.140 | 1,962 |  |
| 12 to 13.9 months | -0.19 | *-0.42* | *0.04* | 0.105 | 1,572 |  | -0.18 | *-0.42* | *0.07* | 0.151 | 1,563 |  |
| 14 to 15.9 months | -0.04 | *-0.30* | *0.21* | 0.742 | 1,198 |  | -0.08 | *-0.36* | *0.20* | 0.586 | 1,192 |  |
| 16 to 17.9 months | 0.15 | *-0.12* | *0.43* | 0.275 | 1,011 |  | 0.02 | *-0.28* | *0.32* | 0.896 | 1,004 |  |
| 18 to 19.9 months | 0.15 | *-0.32* | *0.63* | 0.528 | 358 |  | 0.00 | *-0.53* | *0.52* | 0.990 | 356 |  |

^†^ regardless of availability of covariates; ^#^ comparing mothers of boys with mothers of girls
