# Supplementary material for: Differences in maternal and early child nutritional status by offspring sex in lowland Nepal
Source: Am J Hum Biol. 2021 Jul 6;34(3):e23637. doi: 10.1002/ajhb.23637 (PMC12086752; doi:10.1002/ajhb.23637)
Supplement: Supplementary file 7 — Table S7. Absolute means of MUAC, BMI, and weight in primigravidae mothers of girls and boys in pregnancy and postpartum, and unadjusted and adjusted coefficients, 95% CIs, and p‐values of differences between mothers of boys versus girls for these outcomes. [file AJHB-34-e23637-s011.docx]

**Supplemental Table 7. Absolute means of MUAC, BMI, and weight in primigravidae mothers of girls and boys in pregnancy and postpartum, and unadjusted and adjusted coefficients, 95% CIs and p values of differences between mothers of boys versus girls for these outcomes**

| **Raw measures†** | **Primigravidae Pregnancy MUAC (cm)** | | | | | | **Primigravidae Pregnancy BMI (kg/m^2)** | | | | | |
| --- | --- | --- | --- | --- | --- | --- | --- | --- | --- | --- | --- | --- |
|  | **Female** | | | **Male** | | | **Female** | | | **Male** | | |
| Gestational age | Mean | *SD* | n | Mean | *SD* | n | Mean | *SD* | n | Mean | *SD* | n |
| 12 to 15.9 weeks | 23.2 | 2.0 | 63 | 23.2 | 2.0 | 75 | 19.8 | 2.5 | 63 | 19.9 | 2.2 | 75 |
| 16 to 19.9 weeks | 23.5 | 1.9 | 112 | 23.3 | 1.7 | 116 | 20.3 | 1.9 | 112 | 20.3 | 1.8 | 116 |
| 20 to 23.9 weeks | 23.3 | 1.8 | 120 | 23.6 | 2.0 | 150 | 21.0 | 2.1 | 120 | 20.8 | 2.0 | 150 |
| 24 to 27.9 weeks | 23.5 | 2.0 | 149 | 23.5 | 2.1 | 161 | 21.6 | 2.4 | 149 | 21.6 | 2.3 | 161 |
| 28 to 31.9 weeks | 23.8 | 1.9 | 118 | 23.7 | 2.0 | 133 | 22.1 | 2.1 | 117 | 22.0 | 1.9 | 133 |
| 32 to 35.9 weeks | 23.8 | 2.0 | 203 | 23.6 | 2.1 | 221 | 22.6 | 2.3 | 200 | 22.4 | 2.0 | 216 |
| 36 to 39.9 weeks | 23.5 | 1.9 | 153 | 23.6 | 2.0 | 154 | 22.2 | 2.1 | 151 | 22.9 | 2.3 | 151 |
| **All cases 12 to 29.9 weeks** | **23.6** | **2.0** | **918** | **23.5** | **2.0** | **1,010** | **21.6** | **2.4** | **912** | **21.6** | **2.3** | **1,002** |
| **Unadjusted Coefficients ^#^** | **Primigravidae Pregnancy MUAC (cm)** | | | | | | **Primigravidae Pregnancy BMI (kg/m^2)** | | | | | |
| Gestational age | Unadjusted Coeff | *Upper 95% CI* | *Lower 95% CI* | *p* | *n* |  | Unadjusted Coeff | *Upper 95% CI* | *Lower 95% CI* | *p* | *n* |  |
| 12 to 15.9 weeks | 0.01 | *-0.65* | *0.67* | 0.979 | 138 |  | 0.11 | *-0.67* | *0.89* | 0.787 | 138 |  |
| 16 to 19.9 weeks | -0.18 | *-0.64* | *0.28* | 0.441 | 228 |  | -0.05 | *-0.53* | *0.43* | 0.847 | 228 |  |
| 20 to 23.9 weeks | 0.25 | *-0.21* | *0.70* | 0.288 | 270 |  | -0.25 | *-0.74* | *0.24* | 0.314 | 270 |  |
| 24 to 27.9 weeks | -0.01 | *-0.47* | *0.45* | 0.979 | 311 |  | -0.05 | *-0.57* | *0.46* | 0.844 | 311 |  |
| 28 to 31.9 weeks | -0.14 | *-0.62* | *0.34* | 0.559 | 251 |  | -0.09 | *-0.59* | *0.41* | 0.721 | 250 |  |
| 32 to 35.9 weeks | -0.16 | *-0.54* | *0.23* | 0.418 | 427 |  | -0.19 | *-0.60* | *0.22* | 0.368 | 419 |  |
| 36 to 39.9 weeks | 0.15 | *-0.27* | *0.57* | 0.482 | 313 |  | 0.69 | *0.20* | *1.17* | **0.006** | 308 |  |
| **Adjusted Coefficients^#^** | **Primigravidae Pregnancy MUAC (cm)** | | | | | | **Primigravidae Pregnancy BMI (kg/m^2)** | | | | | |
| Gestational age | Adjusted Coeff | *Upper 95% CI* | *Lower 95% CI* | *p* | *n* |  | Adjusted Coeff | *Upper 95% CI* | *Lower 95% CI* | *p* | *n* |  |
| 12 to 15.9 weeks | -0.08 | *-0.74* | *0.59* | 0.821 | 137 |  | 0.09 | *-0.70* | *0.87* | 0.829 | 137 |  |
| 16 to 19.9 weeks | -0.13 | *-0.57* | *0.32* | 0.569 | 228 |  | 0.01 | *-0.46* | *0.49* | 0.958 | 228 |  |
| 20 to 23.9 weeks | 0.22 | *-0.24* | *0.67* | 0.353 | 270 |  | -0.28 | *-0.76* | *0.21* | 0.264 | 270 |  |
| 24 to 27.9 weeks | 0.05 | *-0.41* | *0.50* | 0.835 | 310 |  | -0.02 | *-0.53* | *0.49* | 0.942 | 310 |  |
| 28 to 31.9 weeks | -0.07 | *-0.54* | *0.41* | 0.789 | 251 |  | -0.04 | *-0.54* | *0.46* | 0.876 | 256 |  |
| 32 to 35.9 weeks | -0.16 | *-0.55* | *0.23* | 0.424 | 421 |  | -0.14 | *-0.56* | *0.27* | 0.493 | 414 |  |
| 36 to 39.9 weeks | 0.19 | *-0.23* | *0.62* | 0.375 | 307 |  | 0.77 | *0.28* | *1.26* | **0.002** | 302 |  |
| **Raw measures†** | **Primigravidae Postpartum MUAC (cm)** | | | | | | **Primigravidae Postpartum BMI (kg/m^2)** | | | | | |
|  | **Female** | | | **Male** | | | **Female** | | | **Male** | | |
| Time since delivery | Mean | *SD* | n | Mean | *SD* | n | Mean | *SD* | n | Mean | *SD* | n |
| 0 to 1.9 months | 23.46 | 2.09 | 253 | 23.54 | 2.08 | 277 | 20.56 | 2.39 | 236 | 20.67 | 2.20 | 261 |
| 2 to 3.9 months | 23.33 | 2.08 | 337 | 23.58 | 2.14 | 379 | 20.32 | 2.37 | 307 | 20.36 | 2.50 | 348 |
| 4 to 5.9 months | 23.46 | 2.34 | 213 | 23.51 | 2.28 | 246 | 19.68 | 2.59 | 203 | 19.76 | 2.43 | 233 |
| 6 to 7.9 months | 23.68 | 2.31 | 267 | 23.47 | 2.07 | 304 | 19.73 | 2.34 | 265 | 19.53 | 2.18 | 300 |
| 8 to 10.9 months | 23.44 | 2.30 | 290 | 23.37 | 2.25 | 303 | 19.57 | 2.62 | 289 | 19.44 | 2.24 | 302 |
| 10 to 11.9 months | 23.34 | 2.21 | 263 | 23.03 | 2.14 | 317 | 19.29 | 2.38 | 262 | 19.23 | 2.37 | 314 |
| 12 to 13.9 months | 23.16 | 2.22 | 207 | 22.79 | 1.94 | 239 | 19.04 | 2.26 | 206 | 18.94 | 2.04 | 238 |
| 14 to 15.9 months | 23.09 | 2.16 | 160 | 23.03 | 1.96 | 183 | 19.21 | 2.47 | 157 | 19.05 | 2.24 | 183 |
| 16 to 17.9 months | 23.17 | 2.11 | 137 | 23.22 | 1.98 | 154 | 19.43 | 2.25 | 137 | 19.30 | 2.11 | 151 |
| 18 to 19.9 months | 22.98 | 1.83 | 53 | 23.53 | 2.38 | 64 | 19.40 | 2.38 | 53 | 19.42 | 2.55 | 63 |
| **All cases 0 to 19.9 months** | **23.36** | **2.20** | **2,180** | **23.32** | **2.13** | **2,466** | **19.69** | **2.46** | **2,115** | **19.63** | **2.35** | **2,393** |
| **Unadjusted Coefficients^#^** | **Primigravidae Postpartum MUAC (cm)** | | | | | | **Primigravidae Postpartum BMI (kg/m^2)** | | | | | |
| Time since delivery | Unadjusted Coeff | *Upper 95% CI* | *Lower 95% CI* | *p* | *n* |  | Unadjusted Coeff | *Upper 95% CI* | *Lower 95% CI* | *p* | *n* |  |
| 0 to 1.9 months | 0.07 | *-0.28* | *0.42* | 0.689 | 543 |  | 0.09 | *-0.31* | *0.49* | 0.661 | 508 |  |
| 2 to 3.9 months | 0.25 | *-0.05* | *0.55* | 0.099 | 726 |  | 0.09 | *-0.27* | *0.45* | 0.632 | 662 |  |
| 4 to 5.9 months | 0.03 | *-0.39* | *0.44* | 0.897 | 469 |  | 0.04 | *-0.43* | *0.51* | 0.872 | 444 |  |
| 6 to 7.9 months | -0.24 | *-0.59* | *0.12* | 0.198 | 572 |  | -0.20 | *-0.57* | *0.17* | 0.288 | 566 |  |
| 8 to 10.9 months | -0.09 | *-0.45* | *0.27* | 0.618 | 598 |  | -0.14 | *-0.53* | *0.25* | 0.471 | 595 |  |
| 10 to 11.9 months | -0.31 | *-0.66* | *0.05* | 0.090 | 581 |  | -0.06 | *-0.45* | *0.33* | 0.771 | 577 |  |
| 12 to 13.9 months | -0.39 | *-0.77* | *-0.01* | 0.046 | 452 |  | -0.15 | *-0.54* | *0.25* | 0.460 | 450 |  |
| 14 to 15.9 months | -0.08 | *-0.50* | *0.34* | 0.708 | 356 |  | -0.21 | *-0.70* | *0.27* | 0.392 | 353 |  |
| 16 to 17.9 months | 0.09 | *-0.37* | *0.56* | 0.691 | 298 |  | -0.04 | *-0.54* | *0.46* | 0.870 | 295 |  |
| 18 to 19.9 months | 0.53 | *-0.23* | *1.29* | 0.172 | 121 |  | 0.02 | *-0.86* | *0.90* | 0.964 | 120 |  |
| **Adjusted Coefficients ^#^** | **Primigravidae Postpartum MUAC (cm)** | | | | | | **Primigravidae Postpartum BMI (kg/m^2)** | | | | | |
| Grouping of time since delivery | Adjusted Coeff | *Upper 95% CI* | *Lower 95% CI* | *p* | *n* |  | Adjusted Coeff | *Upper 95% CI* | *Lower 95% CI* | *p* | *n* |  |
| 0 to 1.9 months | 0.15 | *-0.20* | *0.51* | 0.400 | 530 |  | 0.21 | *-0.20* | *0.61* | 0.324 | 497 |  |
| 2 to 3.9 months | 0.24 | *-0.06* | *0.54* | 0.113 | 716 |  | 0.06 | *-0.30* | *0.42* | 0.749 | 655 |  |
| 4 to 5.9 months | 0.03 | *-0.39* | *0.45* | 0.880 | 459 |  | 0.04 | *-0.42* | *0.51* | 0.854 | 436 |  |
| 6 to 7.9 months | -0.25 | *-0.61* | *0.11* | 0.174 | 571 |  | -0.20 | *-0.57* | *0.18* | 0.300 | 565 |  |
| 8 to 10.9 months | -0.13 | *-0.49* | *0.23* | 0.486 | 593 |  | -0.18 | *-0.57* | *0.21* | 0.355 | 591 |  |
| 10 to 11.9 months | -0.33 | *-0.68* | *0.03* | 0.070 | 580 |  | -0.07 | *-0.46* | *0.33* | 0.746 | 576 |  |
| 12 to 13.9 months | -0.37 | *-0.75* | *0.02* | 0.063 | 446 |  | -0.15 | *-0.55* | *0.24* | 0.450 | 444 |  |
| 14 to 15.9 months | -0.06 | *-0.48* | *0.36* | 0.786 | 343 |  | -0.16 | *-0.65* | *0.33* | 0.513 | 340 |  |
| 16 to 17.9 months | -0.07 | *-0.53* | *0.40* | 0.780 | 291 |  | -0.25 | *-0.75* | *0.26* | 0.339 | 288 |  |
| 18 to 19.9 months | 0.17 | *-0.60* | *0.93* | 0.670 | 117 |  | -0.49 | *-1.38* | *0.39* | 0.273 | 116 |  |

^†^ regardless of availability of covariates; ^#^ comparing mothers of boys with mothers of girls
