# Supplementary material for: Differences in maternal and early child nutritional status by offspring sex in lowland Nepal
Source: Am J Hum Biol. 2021 Jul 6;34(3):e23637. doi: 10.1002/ajhb.23637 (PMC12086752; doi:10.1002/ajhb.23637)
Supplement: Supplementary file 8 — Table S8. Absolute estimates of dietary diversity, adequate DDS, Main meals/d, and snacks in mothers of boys and girls and the coefficients/odds ratios for differences in these outcomes by child sex. [file AJHB-34-e23637-s002.docx]

**Supplemental Table 8. Absolute estimates of dietary diversity, adequate DDS, Main meals/d and snacks in mothers of boys and girls and the coefficients / odds ratios for differences in these outcomes by child sex**

| **Raw measures†** | **Women's Dietary Diversity Score (WDDS)** | | | | | | **Main meals/day** | | | | | | **Snacks/day** | | | | | | **Adequate Dietary Diversity (proportion consuming 5 or more food groups in last 24 hours)** | | | | | | |
| --- | --- | --- | --- | --- | --- | --- | --- | --- | --- | --- | --- | --- | --- | --- | --- | --- | --- | --- | --- | --- | --- | --- | --- | --- | --- |
|  | Mothers of girls | | | Mothers of boys | | | Mothers of girls | | | Mothers of boys | | | Mothers of girls | | | Mothers of boys | | | Mothers of girls | | | | Mothers of boys | | |
| Gestational ages | Mean | *SD* | *n* | Mean | *SD* | *n* | Mean | *SD* | *n* | Mean | *SD* | *n* | Mean | *SD* | *n* | Mean | *SD* | *n* | Mean | *SD* | *n* | Mean | | *SD* | *n* |
| 12 to 15.9 weeks | 4.46 | *1.45* | 194 | 4.34 | *1.55* | 220 | 1.42 | *0.72* | 207 | 1.38 | *0.76* | 232 | 1.87 | *0.39* | 207 | 1.91 | *0.34* | 232 | 0.428 | *0.496* | 194 | 0.414 | | *0.494* | 220 |
| 16 to 19.9 weeks | 4.22 | *1.45* | 295 | 4.37 | *1.58* | 291 | 1.48 | *0.83* | 312 | 1.48 | *0.76* | 304 | 1.91 | *0.41* | 312 | 1.90 | *0.42* | 304 | 0.417 | *0.494* | 295 | 0.436 | | *0.497* | 291 |
| 20 to 23.9 weeks | 4.51 | *1.61* | 322 | 4.46 | *1.45* | 392 | 1.53 | *0.80* | 336 | 1.58 | *0.80* | 404 | 1.93 | *0.38* | 336 | 1.99 | *0.39* | 404 | 0.491 | *0.501* | 322 | 0.477 | | *0.500* | 392 |
| 24 to 27.9 weeks | 4.44 | *1.45* | 394 | 4.50 | *1.56* | 446 | 1.52 | *0.77* | 422 | 1.49 | *0.76* | 474 | 1.94 | *0.42* | 422 | 1.94 | *0.41* | 474 | 0.464 | *0.499* | 394 | 0.491 | | *0.500* | 446 |
| 28 to 31.9 weeks | 4.45 | *1.42* | 302 | 4.39 | *1.52* | 349 | 1.71 | *0.79* | 323 | 1.63 | *0.79* | 366 | 1.76 | *0.65* | 323 | 1.75 | *0.62* | 366 | 0.457 | *0.499* | 302 | 0.461 | | *0.499* | 349 |
| 32 to 35.9 weeks | 4.41 | *1.44* | 543 | 4.47 | *1.44* | 633 | 1.98 | *0.51* | 588 | 1.95 | *0.54* | 670 | 1.42 | *0.71* | 588 | 1.48 | *0.71* | 670 | 0.444 | *0.497* | 543 | 0.461 | | *0.499* | 633 |
| 36 to 39.9 weeks | 4.45 | *1.52* | 438 | 4.42 | *1.38* | 396 | 2.00 | *0.48* | 458 | 1.98 | *0.51* | 419 | 1.47 | *0.69* | 458 | 1.44 | *0.71* | 419 | 0.484 | *0.500* | 438 | 0.477 | | *0.500* | 396 |
| **All cases 12 to 29.9 weeks** | **4.42** | ***1.48*** | **2,488** | **4.43** | ***1.48*** | **2,727** | **1.72** | ***0.72*** | **2,646** | **1.69** | ***0.73*** | **2,869** | **1.71** | ***0.61*** | **2,646** | **1.73** | ***0.60*** | **2,869** | **0.457** | ***0.498*** | **2,488** | **0.464** | | ***0.499*** | **2,727** |
| Unadjusted Coefficients^#^ | **Women's Dietary Diversity Score (WDDS)** | | | | | | **Main meals/d** | | | | | | **Snacks/d** | | | | | | **Adequate Dietary Diversity (proportion consuming 5 or more food groups in last 24 hours)** | | | | | | |
| Gestational ages | Unadjusted Coeff | *Upper 95% CI* | *Lower 95% CI* | *p* | *n* |  | Unadjusted Coeff | *Upper 95% CI* | *Lower 95% CI* | *p* | *n* |  | Unadjusted Coeff | *Upper* | *Lower 95% CI* | *p* | *n* |  | Unadjusted Odds Ratio | *Upper95% CI* | *Lower 95% CI* | *p* | | *n* |  |
| 12 to 15.9 weeks | -0.08 | *-0.35* | *0.20* | 0.572 | *414* |  | -0.04 | *-0.18* | *0.09* | 0.531 | *439* |  | 0.04 | *-0.03* | *0.11* | 0.263 | *439* |  | -0.01 | *-0.44* | *0.41* | 0.954 | | *414* |  |
| 16 to 19.9 weeks | 0.12 | *-0.11* | *0.35* | 0.318 | *586* |  | 0.00 | *-0.12* | *0.12* | 0.982 | *616* |  | -0.02 | *-0.08* | *0.05* | 0.558 | *616* |  | 0.05 | *-0.31* | *0.41* | 0.786 | | *586* |  |
| 20 to 23.9 weeks | -0.09 | *-0.30* | *0.13* | 0.421 | *714* |  | 0.01 | *-0.10* | *0.12* | 0.833 | *740* |  | 0.05 | *-0.01* | *0.10* | 0.100 | *740* |  | -0.10 | *-0.42* | *0.22* | 0.543 | | *714* |  |
| 24 to 27.9 weeks | 0.06 | *-0.13* | *0.24* | 0.559 | *840* |  | -0.03 | *-0.13* | *0.07* | 0.552 | *896* |  | -0.01 | *-0.06* | *0.05* | 0.851 | *896* |  | 0.15 | *-0.16* | *0.45* | 0.345 | | *840* |  |
| 28 to 31.9 weeks | -0.03 | *-0.24* | *0.19* | 0.791 | *651* |  | -0.08 | *-0.19* | *0.03* | 0.142 | *689* |  | 0.01 | *-0.09* | *0.10* | 0.917 | *689* |  | 0.07 | *-0.28* | *0.41* | 0.706 | | *651* |  |
| 32 to 35.9 weeks | 0.03 | *-0.13* | *0.18* | 0.726 | *1,176* |  | -0.04 | *-0.09* | *0.02* | 0.193 | *1,258* |  | 0.06 | *-0.01* | *0.14* | 0.112 | *1,258* |  | 0.06 | *-0.19* | *0.31* | 0.620 | | *1,176* |  |
| 36 to 39.9 weeks | 0.00 | *-0.19* | *0.19* | 0.988 | *834* |  | -0.01 | *-0.08* | *0.05* | 0.667 | *877* |  | -0.03 | *-0.11* | *0.06* | 0.536 | *877* |  | -0.01 | *-0.30* | *0.27* | 0.930 | | *834* |  |
| All mothers | **0.01** | ***-0.06*** | ***0.09*** | 0.759 | **5,215** |  | **-0.03** | ***-0.07*** | ***0.01*** | 0.112 | **5,515** |  | **0.02** | ***-0.01*** | ***0.05*** | 0.234 | **5,515** |  | **0.02** | ***-0.09*** | ***0.14*** | 0.688 | | **5,215** |  |
| Adjusted Coefficients^#^ | **Women's Dietary Diversity Score (WDDS)** | | | | | | **Main meals/day** | | | | | | **Snacks/day** | | | | | | **Adequate Dietary Diversity (proportion consuming 5 or more food groups in last 24 hours)** | | | | | | |
| Gestational ages | Adjusted Coeff | *Upper 95% CI* | *Lower 95% CI* | *p* | *n* |  | Adjusted Coeff | *Upper95% CI* | *Lower 95% CI* | *p* | *n* |  | Adj’ Coeff | *Upper 95% CI* | *Lower 95% CI* | *p* | *n* |  | Adj’ Odds Ratio | *Upper95% CI* | *Lower 95% CI* | *p* | | *n* |  |
| 12 to 15.9 weeks | -0.05 | *-0.32* | *0.23* | 0.743 | *412* |  | -0.06 | *-0.19* | *0.08* | 0.398 | *437* |  | 0.04 | *-0.03* | *0.11* | 0.226 | *437* |  | 0.02 | *-0.41* | *0.45* | 0.936 | | *412* |  |
| 16 to 19.9 weeks | 0.08 | *-0.14* | *0.31* | 0.470 | *586* |  | 0.00 | *-0.12* | *0.12* | 0.969 | *616* |  | -0.02 | *-0.08* | *0.05* | 0.614 | *616* |  | 0.00 | *-0.37* | *0.37* | 0.996 | | *586* |  |
| 20 to 23.9 weeks | -0.09 | *-0.30* | *0.12* | 0.414 | *714* |  | 0.02 | *-0.09* | *0.13* | 0.756 | *740* |  | 0.05 | *0.00* | *0.11* | 0.056 | *740* |  | -0.11 | *-0.44* | *0.22* | 0.500 | | *714* |  |
| 24 to 27.9 weeks | 0.01 | *-0.18* | *0.19* | 0.946 | *839* |  | -0.03 | *-0.13* | *0.07* | 0.556 | *894* |  | -0.02 | *-0.07* | *0.04* | 0.542 | *894* |  | 0.08 | *-0.23* | *0.39* | 0.605 | | *839* |  |
| 28 to 31.9 weeks | -0.07 | *-0.28* | *0.14* | 0.515 | *651* |  | -0.08 | *-0.18* | *0.03* | 0.156 | *689* |  | -0.02 | *-0.11* | *0.07* | 0.673 | *689* |  | 0.00 | *-0.35* | *0.35* | 0.999 | | *651* |  |
| 32 to 35.9 weeks | 0.01 | *-0.14* | *0.16* | 0.868 | *1,170* |  | -0.04 | *-0.09* | *0.02* | 0.188 | *1,252* |  | 0.05 | *-0.02* | *0.13* | 0.155 | *1,252* |  | 0.04 | *-0.22* | *0.29* | 0.783 | | *1,170* |  |
| 36 to 39.9 weeks | -0.07 | *-0.25* | *0.12* | 0.492 | *832* |  | -0.02 | *-0.08* | *0.04* | 0.531 | *875* |  | -0.04 | *-0.12* | *0.05* | 0.407 | *875* |  | -0.09 | *-0.38* | *0.21* | 0.553 | | *832* |  |
| **All mothers** | **-0.01** | ***-0.08*** | ***0.07*** | 0.900 | **5,204** |  | **-0.03** | ***-0.06*** | ***0.01*** | 0.142 | **5,515** |  | **0.01** | ***-0.02*** | ***0.04*** | 0.476 | **5,503** |  | **0.01** | ***-0.11*** | ***0.12*** | 0.931 | | **5,204** |  |

† regardless of availability of covariates; ^#^ comparing mothers of boys with mothers of girls
