# Supplementary material for: Differences in maternal and early child nutritional status by offspring sex in lowland Nepal
Source: Am J Hum Biol. 2021 Jul 6;34(3):e23637. doi: 10.1002/ajhb.23637 (PMC12086752; doi:10.1002/ajhb.23637)
Supplement: Supplementary file 9 — Table S9. Coefficients for boys weight and length at birth <=72 h relative to girls, and third‐trimester energy intakes for mothers of boys relative to girls, from separate multilevel mixed regression models by study arm. [file AJHB-34-e23637-s007.docx]

**Supplemental Table 9. Coefficients for boys weight and length at birth <=72 hours relative to girls, and third trimester energy intakes for mothers of boys relative to girls, from separate multilevel mixed regression models by study arm**

|  | **Length (mm) 0 to 7.9 days** | | | | | | **Weight (g) 0 to 7.9 days** | | | | | | | **Maternal energy (Kcal) intake in pregnancy** | | | | | | |
| --- | --- | --- | --- | --- | --- | --- | --- | --- | --- | --- | --- | --- | --- | --- | --- | --- | --- | --- | --- | --- |
|  | **Girls** | | | **Boys** | | | **Girls** | | | **Boys** | | | | **Mothers of girls** | | | | **Mothers of boys** | | |
| All available cases† | Mean (mm) | *SD* | *n* | Mean (mm) | *SD* | *n* | Mean (g) | *SD* | *n* | | Mean (g) | *SD* | *n* | Mean (kcal) | *SD* | *n* | Mean (kcal) | | *SD* | *n* |
| Control | 474.2 | *23.1* | 437 | 480.3 | *2.2* | 504 | 2706 | *395* | 441 | | 2805 | *405* | 508 | 2155 | *520* | 25 | 2191 | | *582* | 34 |
| PLA only | 474.2 | *22.9* | 438 | 478.8 | *2.1* | 492 | 2737 | *438* | 437 | | 2820 | *437* | 494 | 2131 | *531* | 39 | 2034 | | *483* | 62 |
| PLA+Cash transfer | 476.9 | *21.4* | 526 | 481.3 | *2.3* | 550 | 2773 | *434* | 529 | | 2838 | *456* | 556 | 2175 | *580* | 86 | 2141 | | *530* | 94 |
| PLA+Food Supplement | 476.1 | *20.1* | 635 | 482.2 | *2.1* | 676 | 2753 | *407* | 647 | | 2881 | *465* | 692 | 2126 | *441* | 62 | 2312 | | *617* | 68 |
| **All arms** | **475.5** | ***21.7*** | **2,036** | **480.8** | ***2.2*** | **2,222** | **2745** | ***419*** | **2,054** | | **2840** | ***444*** | **2,250** | **2150** | **523** | **212** | **2167** | | **557** | **258** |
| **Unadjusted coefficients of boys vs. girls in each study arm** | Unadjusted (mm) | *95% CI upper* | *95% CI lower* | *p* | *n* |  | Unadjusted (g) | *95% CI upper* | *95% CI lower* | | *p* | *n* |  | Unadjusted Coeff mothers of boys vs. girls | *95% CI upper* | *95% CI lower* | *p* | | *n* |  |
| Control | 55.3 | *28.0* | *82.6* | **<0.001** | 941 |  | 97.37 | *47.17* | *147.56* | | **<0.001** | 949 |  | 78.5 | *-176.4* | *333.5* | 0.546 | | 59 |  |
| PLA only | 49.4 | *23.4* | *75.4* | **<0.001** | 930 |  | 85.26 | *29.30* | *141.22* | | **0.003** | 931 |  | -108.8 | *-291.5* | *73.9* | 0.243 | | 101 |  |
| PLA+Cash transfer | 45.1 | *19.4* | *70.9* | **0.001** | 1,076 |  | 66.56 | *14.22* | *118.91* | | **0.013** | 1,085 |  | -37.8 | *-198.3* | *122.7* | 0.644 | | 180 |  |
| PLA+Food Supplement | 60.3 | *38.0* | *82.6* | **<0.001** | 1,311 |  | 124.98 | *78.23* | *171.74* | | **<0.001** | 1,339 |  | 123.2 | *-42.7* | *289* | 0.146 | | 130 |  |
| **Adjusted coefficients of boys vs. girls in each study arm** | Adjusted (mm) | *95% CI upper* | *95% CI lower* | *p* | *n* |  | Adjusted (g) | *95% CI upper* | *95% CI lower* | | *p* | *n* |  | Adjusted Coeff mothers of boys vs. girls | *95% CI upper* | *95% CI lower* | *p* | | *n* |  |
| Control | 50.5 | *23.5* | *77.4* | **<0.001** | 933 |  | 82.23 | *32.34* | *132.13* | | **0.001** | 941 |  | 37.0 | *-204.4* | *278.3* | 0.764 | | 58 |  |
| PLA only | 40.0 | *13.9* | *66.1* | **0.003** | 924 |  | 57.79 | *1.51* | *114.07* | | **0.044** | 925 |  | -128.8 | *-308.3* | *50.6* | 0.159 | | 96 |  |
| PLA+Cash transfer | 42.1 | *17.1* | *67.1* | **0.001** | 1,069 |  | 63.94 | *12.26* | *115.62* | | **0.015** | 1,078 |  | -10.1 | *-157.8* | *137.7* | 0.894 | | 176 |  |
| PLA+Food Supplement | 62.0 | *40.1* | *83.9* | **<0.001** | 1,303 |  | 131.89 | *85.85* | *177.93* | | **<0.001** | 1,331 |  | 138.2 | *-37.2* | *313.6* | 0.123 | | 127 |  |
| † regardless of availability of covariates |  |  |  |  |  |  |  |  |  | |  |  |  |  |  |  |  | |  |  |
