# Supplementary material for: Differences in maternal and early child nutritional status by offspring sex in lowland Nepal
Source: Am J Hum Biol. 2021 Jul 6;34(3):e23637. doi: 10.1002/ajhb.23637 (PMC12086752; doi:10.1002/ajhb.23637)

Supplemental Figure 1. Participant flow in the Low Birth Weight South Asia Trial showing how mixed longitudinal cohorts were prepared from available cases at different follow-up timepoints.

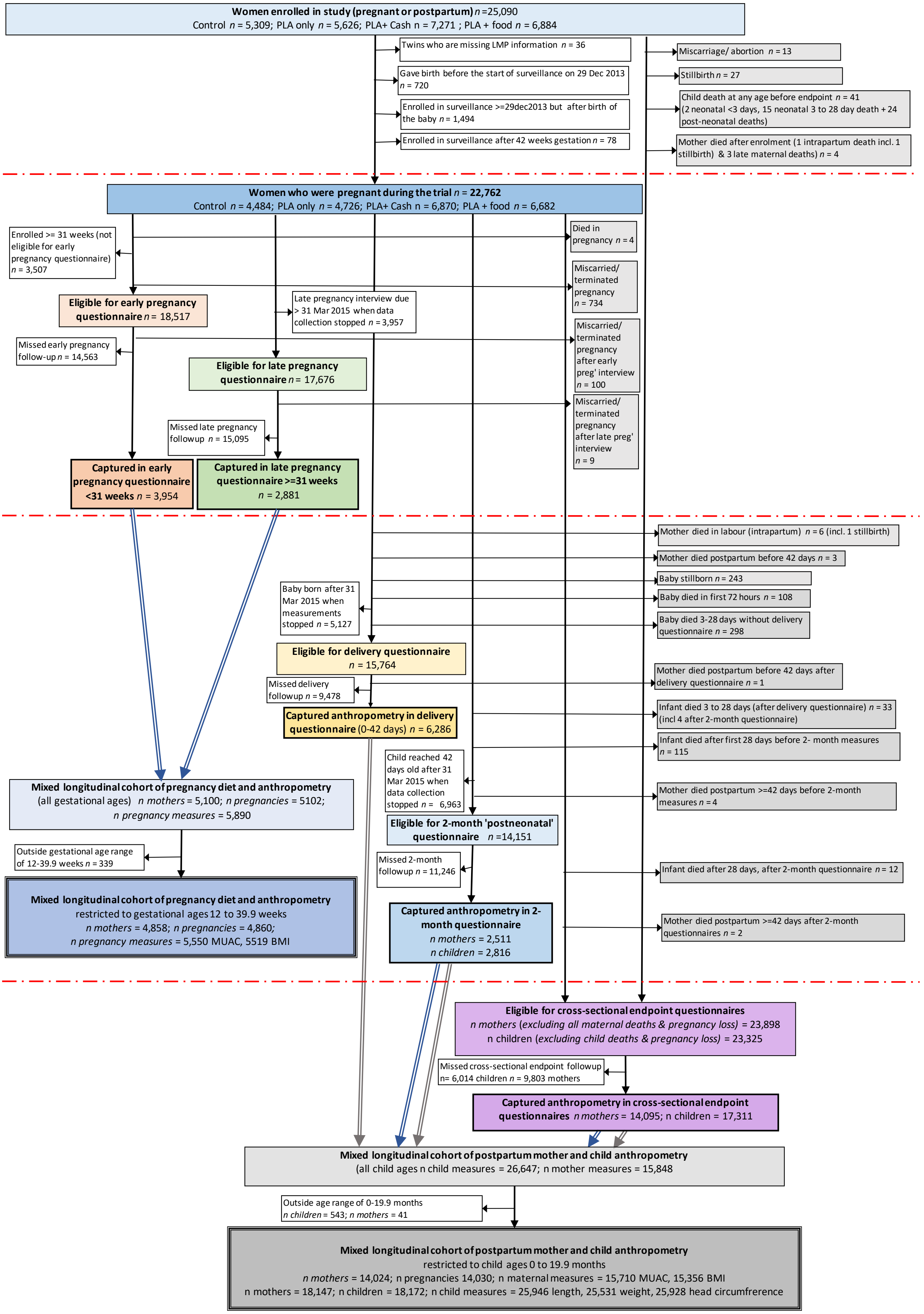

Supplement: Supplementary file 10 — Figure S1. Participant flow in the Low Birth Weight South Asia Trial showing how mixed longitudinal cohorts were prepared from available cases at different follow‐up timepoints. [file AJHB-34-e23637-s005.pdf]
