# Supplementary material for: Differences in maternal and early child nutritional status by offspring sex in lowland Nepal
Source: Am J Hum Biol. 2021 Jul 6;34(3):e23637. doi: 10.1002/ajhb.23637 (PMC12086752; doi:10.1002/ajhb.23637)
Supplement: Supplementary file 11 — Figure S2. Coefficients comparing boy with girl children of primigravidae mothers only in terms of length, weight, head circumference, and z‐scores of length‐for‐age, weight‐for‐length, and head circumference‐for‐age in children 0 to 20 months of age in 2‐monthly age categories. [file AJHB-34-e23637-s003.docx]

Supplemental Figure 2. Coefficients comparing boy with girl children of primigravidae mothers only in terms of length, weight, head circumference, and *z*-scores of length-for-age, weight-for-length and head circumference-for-age in children 0 to 20 months of age in 2-monthly age categories.


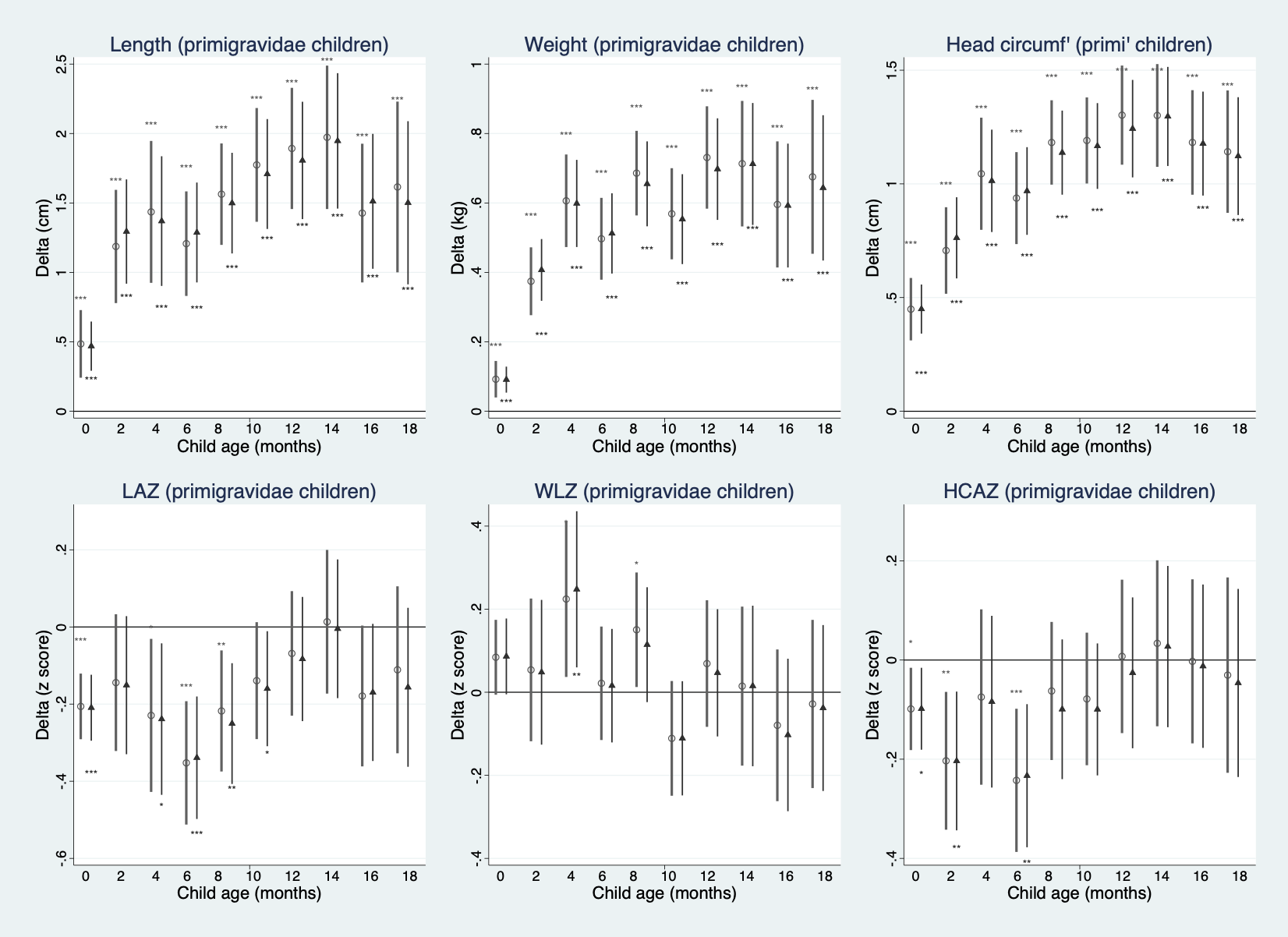


Footnotes: All coefficients come from mixed-effects linear regression models in children of primigravidae women adjusted for study cluster as a random effect. LAZ: Length-for-age *z* score; WLZ: Weight-for-length *z* score; HCAZ: Head circumference-for-age *z* score; Circles represent unadjusted coefficients, triangles represent coefficients adjusted for age of child, mother’s parity, education and asset quintile, study arm of trial and randomisation strata. * indicates p <0.05 for the coefficient plotted above. Age categories 0-1.9, 2-3.9, 4-5.9, 6-7.9, 8-9.9, 10-11.9, 12-13.9, 14-15.9, 16-17.9, 18-19.9 months. A table of regression results including sample sizes (*n*) in each 2-month age category for each pair of adjusted and unadjusted coefficients is provided in Supplemental Table 4.
