# Supplementary material for: Differences in maternal and early child nutritional status by offspring sex in lowland Nepal
Source: Am J Hum Biol. 2021 Jul 6;34(3):e23637. doi: 10.1002/ajhb.23637 (PMC12086752; doi:10.1002/ajhb.23637)
Supplement: Supplementary file 12 — Figure S3. Coefficients comparing primigravidae mothers of boys with primigravidae mothers of girls as the reference category in terms of mid‐upper arm circumference (MUAC) and Body Mass Index (BMI) in 4‐weekly intervals from 12 to 40 weeks' gestation of pregnancy and in 2‐monthly intervals from 0 to 20 months after delivery. [file AJHB-34-e23637-s012.docx]

Supplemental Figure 3. Coefficients comparing primigravidae mothers of boys with primigravidae mothers of girls as the reference category in terms of mid-upper arm circumference (MUAC) and Body Mass Index (BMI) in 4-weekly intervals from 12 to 40 weeks’ gestation of pregnancy and in 2-monthly intervals from 0 to 20 months after delivery.


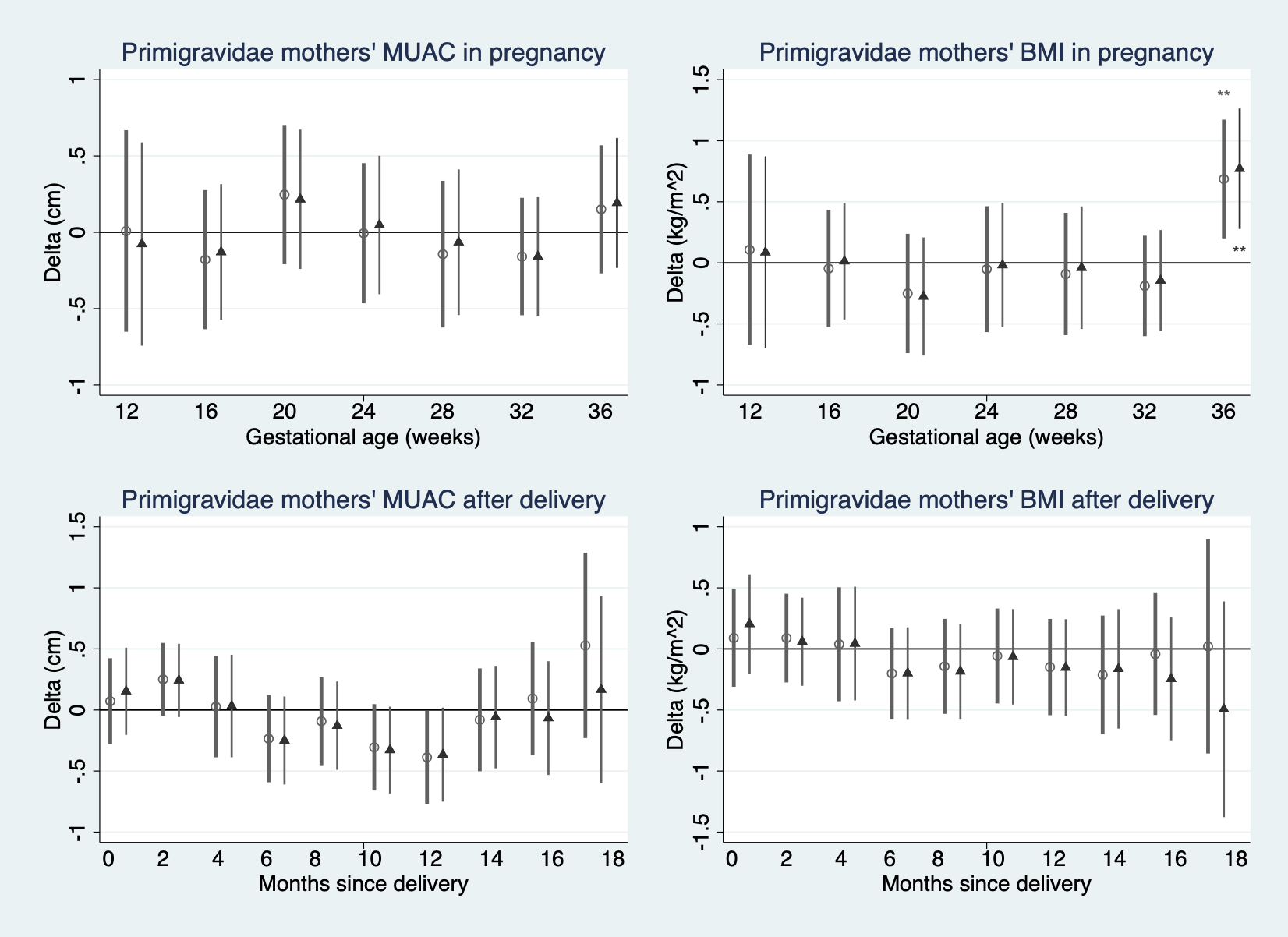


Footnotes: All coefficients come from mixed-effects linear regression models with primigravidae mothers only adjusted for study cluster as a random effect. Circles represent unadjusted coefficients, triangles represent coefficients adjusted for gestational age (in pregnancy models) or age of child (in postpartum models), mother’s parity, education and asset quintile, study arm of trial and randomisation strata in each plot from left to right and top to bottom: Gestational age categories 12-15.9, 16-19.9, 20-23.9, 24-27.9, 28-31.9, 32-35.9, 36-39.9 weeks; Time since delivery categories 0-1.9, 2-3.9, 4-5.9, 6-7.9, 8-9.9, 10-11.9, 12-13.9, 14-15.9, 16-17.9, 18-19.9 months. Asterisks shown above unadjusted and below adjusted coefficient plots indicate: * p<0.05; ** p<0.01; *** p<0.001. A table of regression results including sample sizes (*n*) in each age category is provided in Supplemental Table 7.
