# Supplementary material for: Differences in maternal and early child nutritional status by offspring sex in lowland Nepal
Source: Am J Hum Biol. 2021 Jul 6;34(3):e23637. doi: 10.1002/ajhb.23637 (PMC12086752; doi:10.1002/ajhb.23637)
Supplement: Supplementary file 13 — Figure S4 Different effect of child being male relative to female (blue line) upon length‐for‐age z score in the first 8 days of life by whether the mother was >=145 cm (black line 1–1) or <145 cm (red line 2–2). [file AJHB-34-e23637-s008.docx]

**Supplemental Figure 4. Different effect of child being male relative to female (blue line) upon length-for-age z score in the first 8 days of life by whether the mother was >=145cm (black line 1-1) or <145cm (red line 2-2)**

Footnote: plot of interaction effect between child sex and maternal short stature in a mixed-effects logistic regression model comparing boys’ LAZ with girls, adjusted for child age, mother’s parity, education and asset quintile, study arm of trial and randomisation strata. Sample size (*n* = 3,356). A table of regression results is provided in Supplemental Table 5
